# Iridium-catalyzed α-selective deuteration of alcohols†

**DOI:** 10.1039/d2sc01805e

**Published:** 2022-07-06

**Authors:** Moeko Itoga, Masako Yamanishi, Taro Udagawa, Ayane Kobayashi, Keiko Maekawa, Yoshiji Takemoto, Hiroshi Naka

**Affiliations:** Graduate School of Pharmaceutical Sciences, Kyoto University Kyoto 606-8501 Japan h_naka@pharm.kyoto-u.ac.jp; Department of Chemistry and Biomolecular Science, Faculty of Engineering, Gifu University Yanagido 1-1 Gifu 501-1193 Japan; Faculty of Pharmaceutical Sciences, Doshisha Women's College of Liberal Arts Kodo, Kyotanabe Kyoto 610-0395 Japan

## Abstract

The development of chemoselective C(sp^3^)-H deuteration is of particular interest in synthetic chemistry. We herein report the α-selective, iridium(iii)-bipyridonate-catalyzed hydrogen(H)/deuterium(D) isotope exchange of alcohols using deuterium oxide (D_2_O) as the primary deuterium source. This method enables the direct, chemoselective deuteration of primary and secondary alcohols under basic or neutral conditions without being affected by coordinative functional groups such as imidazole and tetrazole. Successful substrates for deuterium labelling include the pharmaceuticals losartan potassium, rapidosept, guaifenesin, and diprophylline. The deuterated losartan potassium shows higher stability towards the metabolism by CYP2C9 than the protiated analogue. Kinetic and DFT studies indicate that the direct deuteration proceeds through dehydrogenation of alcohol to the carbonyl intermediate, conversion of [Ir^III^–H] to [Ir^III^−D] with D_2_O, and deuteration of the carbonyl intermediate to give the α-deuterated product.

## Abstract 2

An α-selective, iridium(iii)-bipyridonate-catalyzed hydrogen isotope exchange of alcohols using D_2_O has been developed for the direct, chemoselective deuteration of primary and secondary alcohols, thereby providing deuterated bioactive molecules.

## Graphical abstract

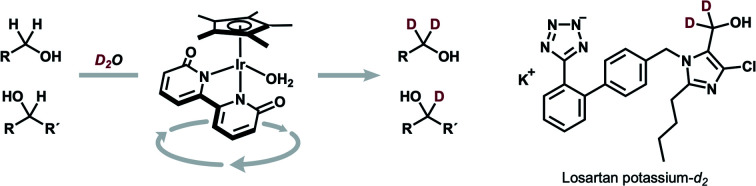

## Introduction

Deuterated organic materials are widely used in various scientific fields such as *in vivo* tracing, proteomics, mechanistic studies, and neutron spectroscopy.^1^ In particular, deuterated drugs are receiving increasing attention, as site-selectively deuterated pharmaceuticals exhibit higher metabolic stability than non-deuterated analogues.^2–4^ Deutetrabenazine, a deuterated analogue of tetrabenazine, is a remarkable example (approved by FDA in 2017, Fig. 1A).^3*a*^ More recently, donafenib, a deuterated sorafenib analogue, received approval in China (2021).^3*b*^ The trend towards the introduction of deuterium (deuterium switch, deut-switch) has increased the need for selective and efficient methods for deuterium incorporation into complex organic molecules.^4^

**Fig. 1:**
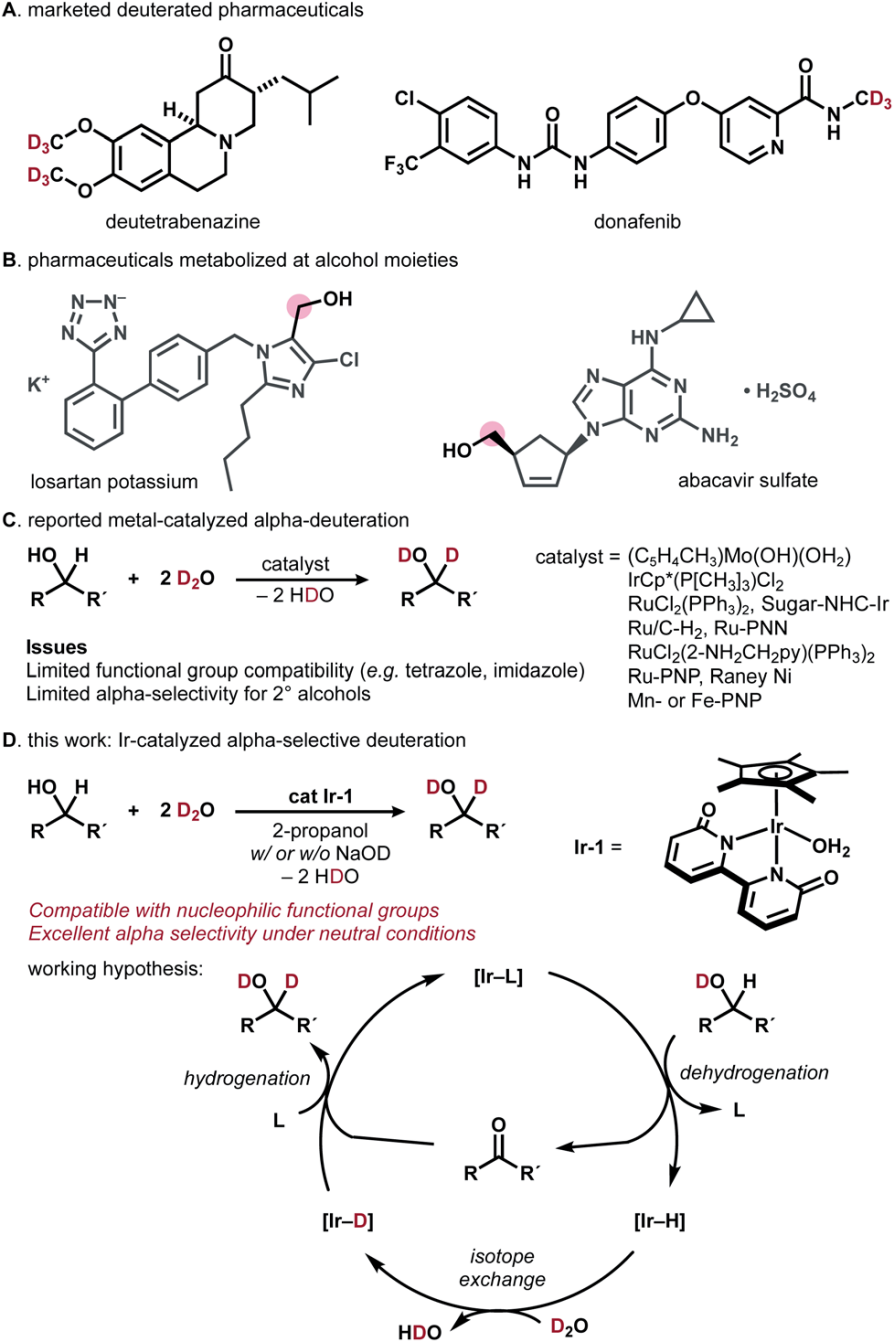
(A) Deuterated pharmaceuticals. (B) Pharmaceuticals metabolized at alcohol moieties. (C) Previous approaches. (D) This work: Ir-catalyzed α-selective deuteration of alcohols.

Chemoselective hydrogen/deuterium isotope exchange (HIE) is an excellent method for chemical deuteration because the product can be quickly accessed from readily available non-deuterated analogues.^5^ C(sp^2^)−H deuteration by homo- or heterogeneous catalysts has been intensively explored and can be used for deuterating pharmaceuticals.^6^ Recently, more challenging chemoselective C(sp^3^)−H deuteration has been of particular interest in the synthetic community.^7–12^ For example, MacMillan and coworkers reported photo-redox-catalyzed direct deuteration of pharmaceuticals,^8*a*^ while Gemmeren reported β-C(sp^3^)−H deuteration of carboxylic acids.^8*b*^

Catalytic deuterium incorporation at the α-position of hydroxyl groups using D_2_O is an attractive strategy to improve the stability of drugs, as the alcohol moiety in pharmaceuticals is often susceptible to metabolism (Fig. 1B).^9^ In 1999, Tyler reported the HIE of alcohols with D_2_O catalyzed by a [(C_5_H_4_CH_3_)Mo(OH)(OH_2_)] complex. The molybdenum-catalyzed system is α-selective for primary alcohols and α,β-selective for secondary alcohols (Fig. 1C).^10^ In 2002, Bergman showed that an [IrCp*(P[CH_3_]_3_)Cl_2_] complex universally deuterates alkyl and aryl C–H bonds including α-carbon of alcohols.^7*a*^ In 2005, Matsubara reported D_2_O-mediated α-selective deuteration of primary alcohols catalyzed by RuCl_2_(PPh_3_)_2_ with or without NaOD.^11^ Nishioka found that a glucopyranoside-incorporated *N-*heterocyclic carbene–iridium complex with AgOTf promotes α-selective deuteration of cyclohexanol.^12^ Sajiki introduced Ru/C-catalyzed, stereo-retentive deuteration of sugar alcohols with D_2_O under an H_2_ atmosphere.^13^ In 2011, Jia reported that a [RuCl_2_(2-NH_2_CH_2_py)(PPh_3_)_2_ (2-NH_2_CH_2_py = 2-aminomethylpyridine)] complex with KOH promotes α- and β-selective alcohol deuteration while [(η^6^-cymene)RuCl_2_]_2_/NH_2_CH_2_CH_2_OH/KOH and [(η^6^-cymene)Ru(NHCHPhCHPhNTs)] deuterate exclusively at β-carbons.^14^ In 2013, Milstein demonstrated that their Ru-PNN pincer complexes catalyze the deuteration of α- and β-carbons of alcohols in the presence of NaOH in D_2_O.^15^ Gunanathan reported a commercially available Ru-PNP pincer complex (Takasago Ru–MACHO complex) effectively catalyzes the α- and β-deuteration of alcohols in the presence of KO^*t*^Bu.^16^ Vermillion reported that RANEY® Ni promotes the HIE of carbohydrates.^17^ Prakash showed the Mn- or Fe-PNP-pincer complex-catalyzed deuteration of alcohols in the presence of NaOH.^18^ These direct α-deuteration of alcohols with D_2_O is often more efficient and economical than the conventional reduction of carbonyl compounds with NaBD_4_, LiAlD_4_, or SiDR_3_/F^−^.^19^ However, α-deuteration of alcohols compatible with coordinative, nucleophilic functional groups is rare.^14–16^ In particular, the deuteration of alcohols bearing imidazole or tetrazole has not been reported to date.^24^

Building on our recent work on photocatalytic N-trideuteromethylation of amines with deuterated methanol leading to deuterated pharmaceuticals,^20^ we envisioned that direct α-deuteration of the hydroxyl group of pharmaceuticals would be achieved by means of hydrogen transfer catalysis using robust metal−ligand bifunctional catalysts.^21^ Herein, we report iridium-catalyzed, α-selective H/D isotope exchange reaction of alcohols using D_2_O as a primary deuterium source (Fig. 1D). The chemoselective deuteration of various alcohols proceeded under basic or neutral conditions without being affected by coordinative functional groups and could be successfully used for deuterium-labelling of pharmaceuticals. We chose a structurally robust iridium–bipyridonate complex (Ir-1, Fujita complex) for the direct deuteration of alcohols based on pioneering works on iridium catalysts^7*a*^ and other metal complexes.^11,12,14–16^Ir-1 was developed by Fujita for the reversible dehydrogenation of alcohols with exceptionally high functional group tolerance.^22^ Thus, we expected that a hydrogen-transfer catalytic cycle should operate if the Ir-1-mediated dehydrogenation of alcohols is followed by H/D exchange of [Ir–H] with D_2_O to afford [Ir−D] and reductive deuteration of carbonyl compounds by [Ir−D] takes place (Fig. 1D).

## Results and discussion

We selected losartan potassium (1-K^+^) as a target substrate for deuteration with the aim of investigating the difference in metabolism between deuterated and non-deuterated losartan. The introduction of deuterium at the α*-*position of the hydroxyl group of 1-K^+^ should control the C–H bond cleavage during the metabolic oxidation and therefore is of particular interest in medicinal chemistry and metabolic studies.^23^ However, to the best of our knowledge, the deuterium KIE for the metabolic process of 1-K^+^-*d*_2_ has not been reported to date. So far, one synthetic method has been reported for α-deuterated losartan potassium, which involves the use of *N,N*-dimethylformamide-*d*_7_ and NaBD_4_ during eight synthetic steps.^23*b*^ The establishment of a more accessible route to selectively deuterated losartan should therefore contribute to the development of deuterated pharmaceuticals.

Table 1 shows the results of catalytic deuteration of 1-K^+^. Whereas the use of previously reported Ru/C/H_2_ or a Ru-PNP-pincer complex (Ru-MACHO) resulted in no incorporation of deuterium,^24^ we were pleased to find that the iridium bipyridonate complex Ir-1 efficiently promotes the α-deuteration of 1-K^+^ (entry 3 *vs.* entries 1 and 2). When a mixture of 1-K^+^ (0.075 mmol, 0.10 M), Ir-1 (5 mol%), NaOD (15 mol%), 2-propanol (50 mol%), and D_2_O (0.75 mL, 41.6 mmol) was heated at 100 °C for 5 h, we obtained 1-K^+^/Na^+^ with 55% deuterium incorporation based on ^1^H NMR analysis (entry 3). Prolonging the reaction time to 36 h increased the deuterium incorporation up to the theoretical value (98% *D*) without any side-reaction or undesired deuteration at other carbons (entries 4 and 5). The perfect α-C(sp^3^)−H selectivity is in stark contrast with previously known Ir-catalyzed deuterations, which generally exhibit C(sp^2^)−H preference.^7,25^ The use of the bifunctional, bidentate bipyridonate ligand in Ir-1 is crucial for the selective deuteration at the hydroxyl group C(sp^3^)-H: Ir complexes such as [IrCp*Cl_2_]_2_ or [IrCp*(2-hpy)Cl_2_] (2-hpy = 2-hydroxypyridine)^25^ resulted in no deuterium incorporation (entries 6 and 7). CF_3_-substituted Ir-2 and dimethylamino-substituted Ir-3 were less effective than Ir-1 (entries 8 and 9).^22*d*^ Cationic Ir-4 and anionic Ir-5 also catalyzed the deuteration of 1-K^+^, but less efficiently (entries 10 and 11) probably due to unpreferable ratios of neutral and anionic catalytic intermediates.^22,26^ No deuteration proceeded without Ir-1 or NaOD (entries 12 and 13). The addition of 2-propanol (50 mol%) is critical for keeping Ir-1 active: without 2-propanol, the reaction stops before the deuterated ratio reaches the theoretical H/D value of 98% *D* (entries 14 and 15). The role of 2-propanol is unclear, but we think that dehydrogenation of 2-propanol increases the concentration of active Ir–H complexes, thereby lowering the concentration of an aldehyde intermediate that potentially causes catalyst deactivation.^27^ In fact, we observed a decrease in the ^1^H NMR signal for α-hydrogen of 2-propanol, indicating that 2-propanol is oxidized during the reaction. The deuteration was faster at higher concentrations (0.5 M, entry 16 *vs.* 4). Limited deuterium incorporation (96% *D*) reflecting the higher concentration of the substrate could be further improved using deuterated 2-propanol (98% *D*, entry 17). A scale-up experiment (2 mmol scale) was also successful (entry 18).

**Table 1:** Deuteration of Losartan Potassium (1-K^+^)^a^

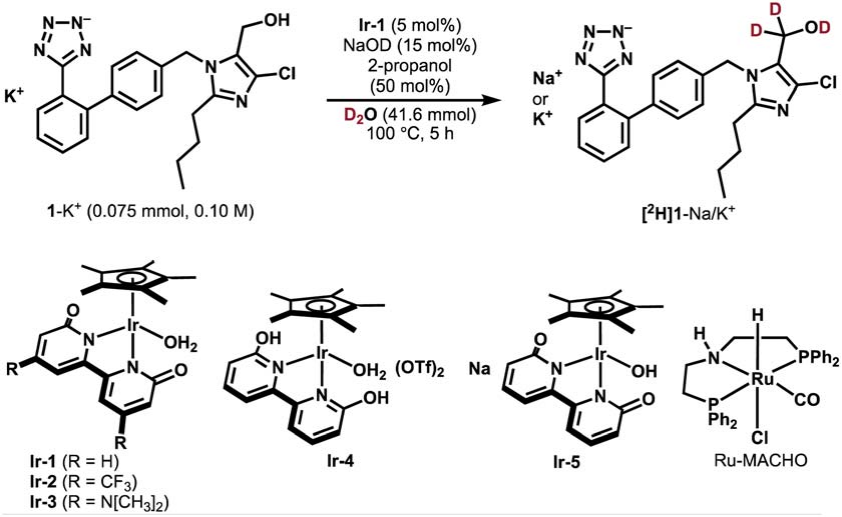		
Entry	Changes from the above scheme	% *D*
1	Ru/C (5 mol% Ru) and H_2_ instead of Ir-1	<3% *D*
2	Ru-MACHO instead of Ir-1	<3% *D*
3	None	55% *D*
4	24 h	94% *D*
5	36 h	98% *D*
6	[IrCp*Cl_2_]_2_ (5 mol% Ir) instead of Ir-1	<3% *D*
7	[IrCp*(2-hpy)Cl_2_] instead of Ir-1	<3% *D*
8	Ir-2 instead of Ir-1	13% *D*
9	Ir-3 instead of Ir-1	30% *D*
10	Ir-4 instead of Ir-1	18% *D*
11	Ir-5 instead of Ir-1	43% *D*
12	Without Ir-1	<3% *D*
13	Without NaOD	<3% *D*
14	Without 2-propanol, 36 h	83% *D*
15	Without 2-propanol, 41 h	85% *D*
16	1-K^+^ (0.50 M), 24 h	96% *D*
17	With 2-propanol-*d*_8_, 1-K^+^ (0.50 M), 24 h	98% *D*
18	1-K^+^ (2.0 mmol, 0.50 M), 24 h	96% *D*

a Conditions: 1-K^+^ (0075 mmol), Ir-1 (5 mol%), NaOD (15 mol%), 2-propanol (50 mol%), D_2_O (0.75 mL, 41.6 mmol), 100 °C. 2-hpy = 2-hydroxypyridine.

Deuterated losartan was easily separated from the Ir catalyst by pH adjustment and extraction. Stepwise neutralization of the reaction mixture with aq H_2_SO_4_ allowed us to extract Ir-1 and the protonated form of deuterated losartan ([^2^H]1) successively into organic layers. The reaction of [^2^H]1 with KOH and reprecipitation in dry acetone^28^ gave the desired losartan potassium-*d*_2_ in 69% overall yield (96% *D*, Fig. S1 in the ESI†).

**Fig. 2:**
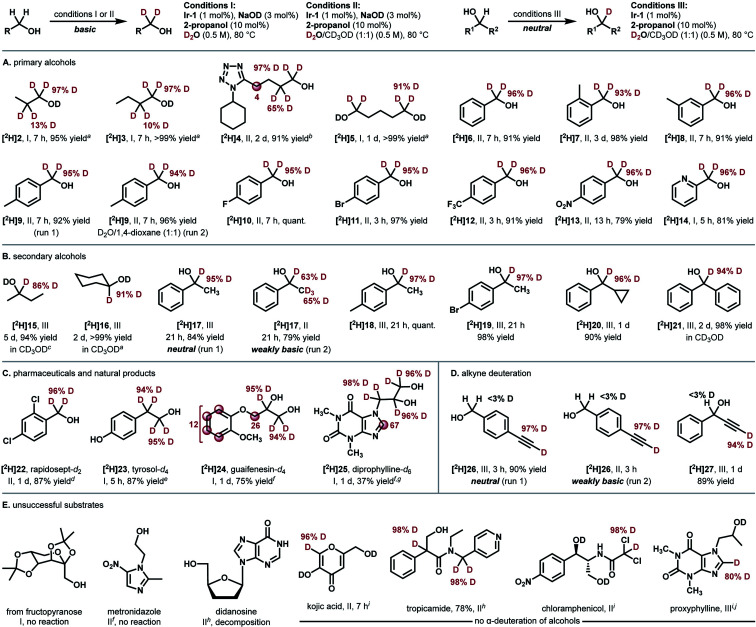
Scope of the reaction. Isolated yields are shown unless otherwise noted. ^*a*^NMR yield using 1,4-dioxane (0.15 mmol) as an internal standard. ^*b*^Ir-1 (5 mol%), NaOD (15 mol%), 0.1 M, 100 °C. ^*c*^NMR yield using maleic acid (0.15 mmol) as an internal standard. ^*d*^Ir-1 (2 mol%), NaOD (6 mol%). ^*e*^NaOD (103 mol%). ^*f*^Ir-1 (5 mol%), NaOD (15 mol%), 100 °C. ^*g*^Low isolated yield due to high hydrophilicity. ^*h*^Ir-1 (5 mol%), NaOD (15 mol%), 0.1 M, 100 °C, 24 h. ^*i*^NMR analysis of the crude mixture. ^*j*^Ir-1 (5 mol%), D_2_O/CD_3_OD (5 : 1), 0.1 M, 100 °C, 24 h.

**Fig. 3:**
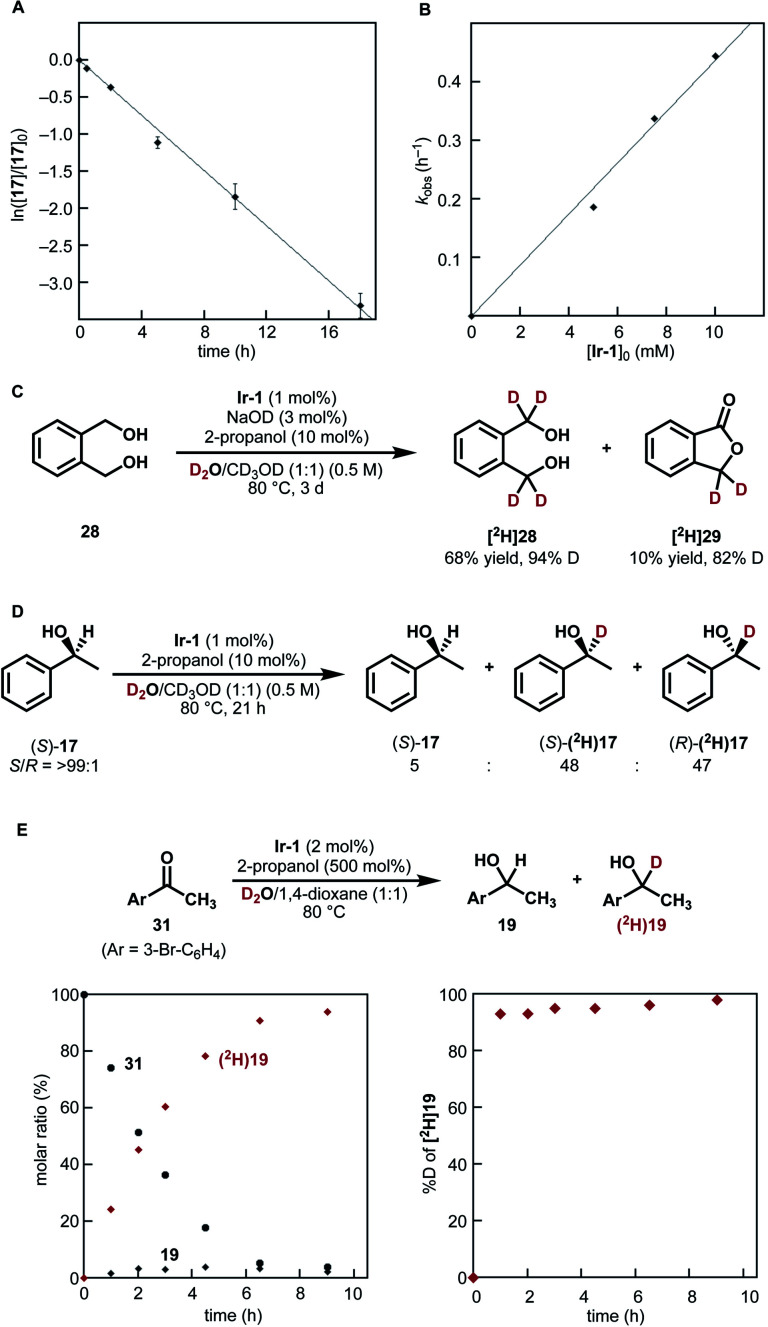
Mechanistic studies. (A) Pseudo-first-order plot of HIE of 1-phenylethanol (17) under the conditions III shown in Fig. 2. Average of three separate runs with standard deviations. (B) The observed rate constants (*k*_obs_, h^−1^) for deuteration of 17 plotted against [Ir-1]_0_ (mM, 1−2 mol% Ir). (C) Lactone formation from diol 28. (D) Racemization in the deuteration of (*S*)-1-phenylethanol (17). (E) Reaction time course of reductive carbonyl deuteration with 2-propanol and D_2_O: (left) molar ratio of 19 and 31; (right) % *D* at the α carbon of [^2^H]19.

We next investigated the substrate scope of the Ir-catalyzed HIE reaction (Fig. 2). 1-Propanol (2) was smoothly deuterated under the optimized conditions shown in Table 1, entry 16 (97% *D* for alpha, >99% yield, *t* = 1.5 h). A comparable result was obtained with a less amount of Ir-1 (1 mol%) under milder conditions (conditions I, 97% *D* for alpha, 95% yield, *t* = 7 h, Fig. 3A). We then adopted these conditions to simpler substrates. The current catalytic system proved to be effective for deuterating various primary alcohols (Fig. 2A). Deuteration of simple primary alcohols proceeded with 1 mol% of Ir-1 at 80 °C. Deuterium was incorporated selectively at the α-positions (97% *D*) with minor incorporation at the β-positions (10–13% *D*). Aliphatic alcohol bearing a tetrazole unit (4) was applicable at 100 °C. Diol 5 was also deuterated selectively at the α-positions. Benzyl alcohols bearing electron-withdrawing or electron-donating substituents underwent the α-deuteration smoothly. For hydrophobic substrates, methanol-*d*_4_ was used as a co-solvent to allow monitoring of the progress of the reaction by ^1^H NMR spectroscopy (conditions II, *e.g.,* 95% *D* for [^2^H]9 (run 1)). In these cases, D_2_O remains the primary deuterium source, as comparable results were obtained with 1,4-dioxane co-solvent under otherwise identical conditions (94% *D* for [^2^H]9 (run 2)).

To our surprise, secondary alcohols underwent the α-deuteration better under neutral conditions (conditions III, Fig. 2B). For example, the α-position of 1-phenylethanol (17) was smoothly deuterated in the absence of NaOD ([^2^H]17 (run 1), 95% *D*). Because the system is under neutral conditions, deuterium was not incorporated at the β-position (<3% *D*). This high α/β-selectivity was not achieved under basic conditions (conditions II, [^2^H]17 (run 2), α: 63% *D*, β: 65% *D*) as in the case of previously reported catalysts. The presence of a cyclopropyl group was tolerated, indicating that radical generation is not involved.^29^ Poorly soluble substrates could also be deuterated by prolonging the reaction time.

Next, this method was extended to synthesize several pharmaceutically relevant deuterated alcohols (Fig. 2C). 2,4-Dichlorobenzyl alcohol (rapidosept, 22, a preservative) was deuterated selectively at the α-position. Tyrosol ([^2^H]23) underwent deuteration at both α- and β-carbons. Guaifenesin (24) and diprophylline (25) were deuterated at the α-position of the hydroxyl groups together with partial deuteration at other moieties. The results shown in Fig. 2A−C demonstrate excellent functional-group tolerance in the deuteration of alcohols without the involvement of side reactions such as dehalogenation (10, 11, 19, and 22), reduction (13), or catalyst deactivation (4, 14, and 25).

In the case of alcohols bearing a terminal alkyne moiety, deuterium was selectively introduced at the alkyne C(sp) carbon prior to the deuteration of the α-carbons of hydroxyl groups (Fig. 2D). The deuteration of alkynes 26 and 27 proceeded even under neutral conditions. High reactivity for alkyne C(sp)-deuteration under neutral conditions would be favourable for the selective deuteration of alkyne-tagged probes for Raman imaging.^30,31^

Examples of unsuccessful substrates are also shown in Fig. 2E. Sterically hindered alcohol (protected fructopyranose) and β-amino alcohols were poorly reactive. An *N*,*O-*aminal structure in didanosine was not compatible under the reaction conditions (Fig. 2E, left). While no α-deuteration of alcohols occurred with kojic acid, tropicamide, chloramphenicol, and proxyphylline, acidic carbons in these molecules were selectively deuterated under these conditions (Fig. 2E, right). In fact, these substrates in Fig. 2D and E underwent the deuteration at the acidic carbons spontaneously under basic conditions in the absence of Ir-1.

To better understand the catalytic profile, several mechanistic experiments were conducted. ^1^H NMR analysis of a mixture of 1-phenylethanol (17) and substoichiometric Ir-1 (20 mol%) in CD_3_OD after heating at 60 °C for 2 h indicated the presence of Ir−bipyridonate complexes and [^2^H]17 (15% *D*), together with a trace amount of acetophenone (30) (Fig. S2† in the ESI). The chemical shifts for the observed Ir−bipyridonate complexes are very similar to those for Ir-1 and different from those of dihydroxypyridine complex Ir-4, indicating that Ir−bipyridonate complexes are a resting state in the catalytic cycle.

As shown in Fig. 3A and B, kinetic monitoring of the HIE of 1-phenylethanol (17) by Ir-1 (1 mol%) at 80 °C under neutral conditions showed a first-order dependence on both [17]_0_ and [Ir-1]_0_ (details are shown in Fig. S3 in the ESI†). When 1,2-benzenedimethanol (28) was reacted under these conditions, deuterated lactone [^2^H]29 was obtained in 10% yield (82% *D*) in addition to the desired deuterated diol (68% yield, 94% *D*, Fig. 3C). When optically pure (*S*)-1-phenylethanol (*S*/*R* = >99 : 1) was deuterated under the neutral conditions, the deuterated product was completely racemized (product *S*/*R* = 50 : 50; remaining alcohol *S*/*R* = >99 : 1, Fig. 3D). These results suggest the formation of a carbonyl intermediate (aldehyde or ketone). The lactonization of 29 is the result of the formation of a mono-aldehyde intermediate that undergoes intramolecular cyclization to give a hemiacetal.^32^ The racemization of (*S*)-17 is due to the reduction of acetophenone from both faces of the carbonyl group.

To clarify the reactivity of Ir-1 for the reduction of the carbonyl intermediate as well as to probe the rate of H/D isotope exchange of the iridium hydride intermediate, we next tested the reduction of 4-bromoacetophenone (31) with 2-propanol in the presence of Ir-1 in a D_2_O−1,4-dioxane mixture (Fig. 3E).^33^ Reaction monitoring indicated that the 2-propanol-mediated reductive deuteration of 31 produced [^2^H]19 with a high deuterium content (>93% *D*) at the α-carbon throughout the reaction progress. This result indicates that (1) 2-propanol acts as a reducing agent toward carbonyl compounds in the presence of Ir-1; (2) the conversion of an iridium hydride intermediate [Ir–H] into iridium deuteride [Ir–D] with D_2_O proceeds much faster than the reduction of 4-bromoacetophenone (31) by [Ir–H]. Here again, deuterium incorporation at the β-carbon was less than 5% during this reductive process.

**Fig. 4:**
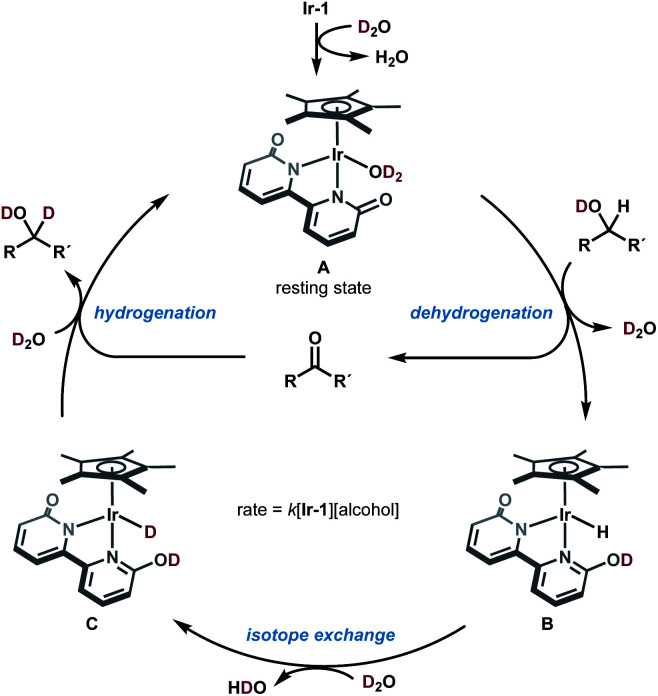
Proposed mechanism for catalytic deuteration of secondary alcohols under neutral conditions.

Based on these mechanistic data, a plausible catalytic cycle of the Ir-catalyzed HIE of alcohols under neutral conditions is shown in Fig. 4. The proposed catalytic cycle involves the generation of D_2_O-coordinated iridium(III) complex A from Ir-1; bipyridonate-ligand-assisted dehydrogenation resulting in the generation of iridium-hydride species B and carbonyls (aldehydes or ketones); Ir–H/D exchange reaction with D_2_O to give iridium deuteride C; reduction of carbonyls by the Ir–D species C to give deuterated alcohol and to regenerate iridium species A. The results of our NMR and kinetic studies indicate that (1) the catalytic cycle is mediated by monomeric iridium species, and (2) the resting state is observable complex A. β-Deuteration of alcohols under basic conditions occurs *via* base-catalyzed enolization of the carbonyl intermediates, but the enolization of ketones can be suppressed under our neutral conditions.

**Fig. 5:**
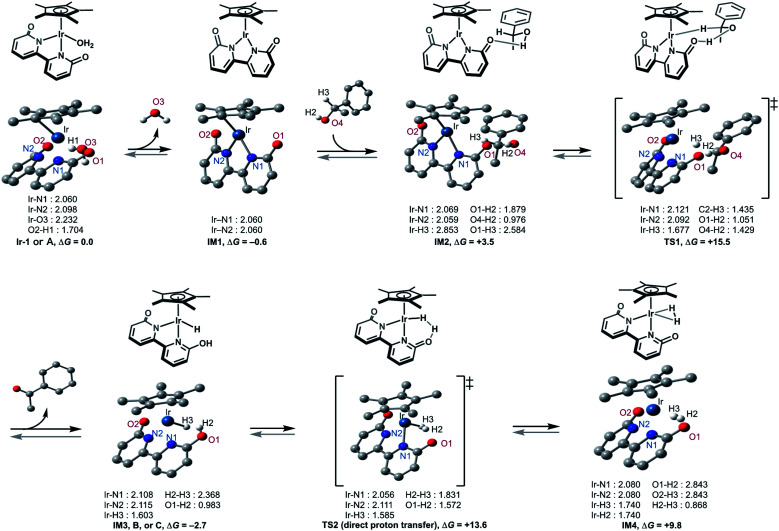
Geometry and energy changes of the proposed pathway at the CAM-B3LYP + GD3 level of theory using SDD and 6–31++G** basis sets for Ir and other atoms, respectively. Solvent effect (H_2_O) by IEFPCM method; Gibbs free energies in kcal mol^−1^ at *T* = 353.15 K.

This catalytic cycle in Fig. 4 is broadly in line with the experimental results of Fujita *et al.*^22*c*^ and the results of DFT calculations on Ir-1-promoted dehydrogenation of alcohols.^34,35^ To further validate our proposed catalytic cycle, we performed the DFT calculations on the Ir-1-promoted deuteration of 17 to (^2^H)17 under neutral conditions (Fig. 5, S4, and S5†). Intermediate 1 (IM1) and IM3 are comparably stable. The first step is the Ir-1-promoted dehydrogenation of alcohol 17 (transition state 1, TS1). In TS1, the O1⋯H2 and Ir⋯H3 distances are 1.051 Å and 1.677 Å, respectively, meaning that the H_2_ migration is almost done at the transition state, whereas the Ir–H3 bond is being formed. The second step is the proton transfer leading to an η^2^-dihydrogen complex IM4 directly (Fig. 5) or mediated by a 1-phenylethanol or water hydrogen-bonding bridge (Fig. S4†). All these routes seem energetically feasible, while the direct pathway (TS2) is slightly preferred over routes with a hydrogen-bonding bridge (TS3 or TS4). TS1 is similar in free energies to the three TSs 2−4 (Fig. S5†). While a detailed comparison of energy changes with experiments is avoided here due to the limited accuracy of computations in energies, the overall free energy changes are mostly consistent with the experimental observations.

**Fig. 6:**
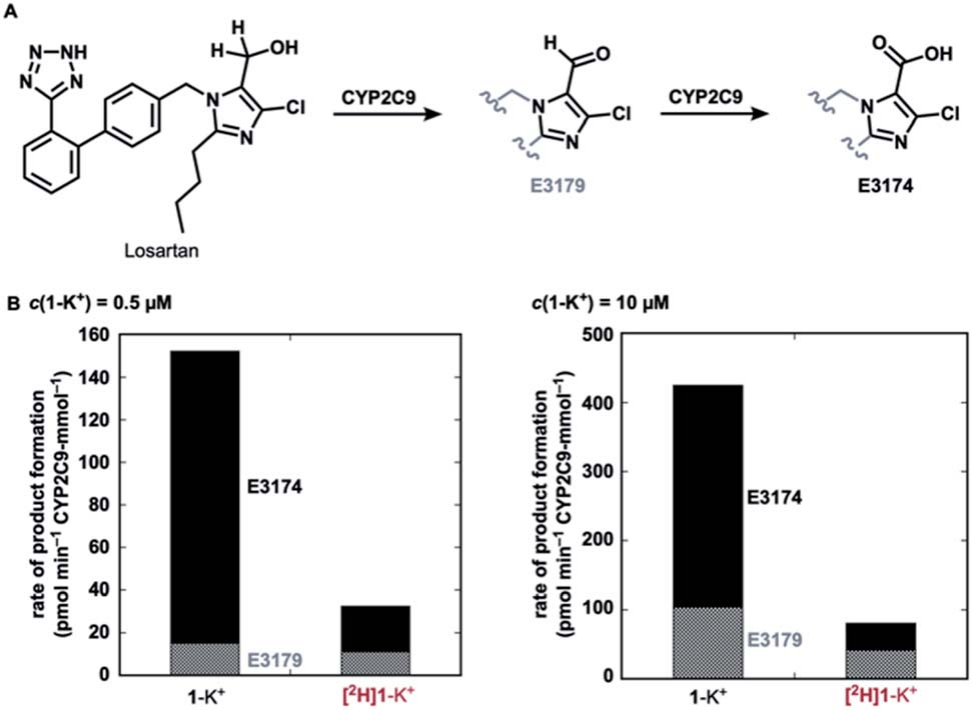
(A) Metabolic pathway of losartan and (B) kinetic data on the formation of metabolites E3179(-*d*_1_) and E3174 from 1-K^+^ or [^2^H]1-K^+^ (96% *D*) by CYP2C9 under non-competitive intermolecular conditions.

Finally, with a practical preparative method of deuterated losartan [^2^H]1-K^+^ in hand, we conducted a preliminary metabolic study using CYP2C9 (Fig. 6). Under non-competitive intermolecular conditions, the deuterated losartan potassium ([^2^H]1-K^+^) was metabolized to metabolites E3179(-*d*_1_) (aldehyde) and then E3174 (carboxylic acid) with a much lower rate than the protiated analogue 1-K^+^. This result clearly demonstrates the significant impact of the deuteration of α-hydrogens in losartan on its metabolic profile and the practical utility of the current synthetic method.

## Conclusions

In summary, we have discovered the iridium−bipyridonate-catalyzed H/D exchange reaction of alcohols. The direct α-deuteration protocol is applicable to several functionalized alcohols including pharmaceuticals. Furthermore, α-selective deuteration of secondary alcohols was achieved under neutral conditions. In addition, deuterated losartan potassium was found to be significantly more stable towards the metabolism by cytochrome P450 (CYP2C9) than the protiated analogue, demonstrating the importance of α-deuteration. The present iridium-catalyzed H/D exchange reaction opens up a new approach for the synthesis of deuterated organic materials and pharmaceuticals.

## Data availability

The data that support the findings of this study are available in the ESI† or on request from the corresponding author.

## Author contributions

M. Y. found the activity of Ir-1 for the deuteration of 1-K^+^. M. I. conducted all the other experiments and wrote the manuscript. T. U. performed the DFT calculations. A. K. conducted the metabolic study. K. M. guided the metabolic study. Y. T. guided the synthetic study. H. N. designed the project, directed the study, and wrote the manuscript.

## Conflicts of interest

There are no conflicts to declare.

## Supplementary Material

SC-013-D2SC01805E-s001

SC-013-D2SC01805E-s002
