# Supplementary material for: Iridium-catalyzed α-selective deuteration of alcohols
Source: Chem Sci. 2022 Jul 6;13(30):8744–51. doi: 10.1039/d2sc01805e (PMC9350590; doi:10.1039/d2sc01805e)
Supplement: SC-013-D2SC01805E-s001 [file SC-013-D2SC01805E-s001.pdf]

Electronic Supplementary Material (ESI) for Chemical Science.
This journal is © The Royal Society of Chemistry 2022

Electronic Supplementary Information 1

Iridium-Catalyzed α-Selective Deuteration of Alcohols

Moeko Itoga,[a] Masako Yamanishi,[a] Taro Udagawa,[b] Ayane Kobayashi,[c] Keiko Maekawa,[c] Yoshiji Takemoto,[a] and Hiroshi Naka*[a]

a. Graduate School of Pharmaceutical Sciences, Kyoto University, Kyoto 606-8501, Japan. Email: h_naka@pharm.kyoto-u.ac.jp

b. Department of Chemistry and Biomolecular Science, Faculty of Engineering, Gifu University, Yanagido 1-1, Gifu 501-1193, Japan.

c. Faculty of Pharmaceutical Sciences, Doshisha Women's College of Liberal Arts, Kodo, Kyotanabe, Kyoto 610-0395, Japan.

Table of Contents

1. General Comments …S02
2. Materials …S02
3. Deuteration of Losartan Potassium (Table S1 and Fig. S1) …S03
4. Catalytic H/D Exchange Reaction …S05
5. Mechanistic Studies …S13
6. NMR Studies (Fig. S2) …S15
7. Kinetic Experiments (Fig. S3) …S16
8. Effect of 2-propanol …S17
9. Computational Details (Fig. S4 and S5) …S17
10. Cartesian Coordinates (Tables S2−S20) …S19
11. Metabolic Studies …S37
12. Reference …S37

1. General Comments

All the reactions were conducted under air under otherwise noted. Silica gel column chromatography was performed using Chromatorex BW-300 (Fuji Silysia, Aichi, Japan). Preparative thin layer chromatography (PTLC) was performed using PLC Silica gel 60 F_{254}, 0.5 mm (Fuji Silysia, Aichi, Japan). Proton nuclear magnetic resonance (^{1}H NMR) spectra were recorded on a JNM-ECA 500 (JEOL, Tokyo, Japan) at 500 MHz or a JNM-ECZ 600 (JEOL) at 600 MHz. Chemical shifts were reported relative to Me_4Si (δ 0.00) in $CDCl_3$ or the residual solvent peak in D_2O (δ 4.79), CD_3OD (δ 3.31), or DMSO-d_6 (δ 2.50). Multiplicity was indicated by one or more of the following: s (singlet); d (doublet); t (triplet); q (quartet); quint (quintet); m (multiplet); br (broad). Proton-decoupled carbon nuclear magnetic resonance ($^{13}C\{^1H\}$ NMR) spectra were recorded on a JNM-ECZ 600 at 151 MHz. Chemical shifts were reported relative to $CDCl_3$ (δ 77.0), CD_3OD (δ 49.0), or DMSO-d_6 (δ 39.5). Signals of the aliphatic C−H bonds were referred to determine the deuteration ratio of substrates. The deuteration ratio of the other substrates was determined with calibration using the starting materials as references. Low- and high-resolution mass spectra were recorded on an LCMS-IT-TOF (Shimadzu, Kyoto, Japan; ESI-MS).

2. Materials

D_2O was purchased from Cambridge Isotope Laboratories. Losartan potassium, rapidosept, tyrosol, guaifenesin, and diprophylline were purchased from TCI. **Ir-1** and **Ir-5** were prepared according to the literature method.[1] **Ir-2** and **Ir-3** were provided by Professor Ken-ichi Fujita (Kyoto University). **Ir-4** was purchased from Kanto Chemicals. NaOD (in D_2O, 0.20 M) was prepared by dissolving NaOH in D_2O and repeated evaporation−addition of D_2O. Other reagents were purchased from Sigma-Aldrich, TCI, Kanto Chemicals, Nacalai, or Wako Chemicals and used as received unless otherwise noted. **4** was synthesized as follows:

4

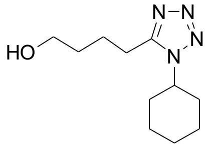

To a mixture of 1-cyclohexyl-5-(4-chlorobutyl)-1*H*-tetrazole (242.2 mg, 1.0 mmol), HCO_2Na (275.5 mg, 4.0 mmol) and tetrabutylammonium bromide (17.0 mg, 0.05 mmol) were added ^{t}BuOH (3 mL). The mixture was heated at 100 °C in a pre-heated oil bath under Ar atmosphere for 2 d. To the reaction mixture was added aq. NaOH (1.1 M, 1.0 mL) dropwise and the mixture was stirred at rt for 10 h. The organic layers were combined and dried over Na_2SO_4. After filtration, the filtrate was concentrated under reduced pressure. The residue was purified by silica gel column chromatography (hexane/EtOAc

= 1:1 to $CHCl_3/CH_3OH$ = 20:1) to afford **4**. A pale orange liquid (186.2 mg, 83%). ^1H NMR (600 MHz, $CDCl_3$) δ: 4.16−4.10 (1H, m), 3.71 (2H, s), 2.89 (2H, t, J = 7.6 Hz), 2.06−1.95 (8H, m), 1.78 (1H, d, J = 13.1 Hz), 1.72−1.68 (2H, m), 1.43 (2H, q, J = 12.2 Hz), 1.35 (1H, t, J = 13.4 Hz); $^{13}C\{^1H\}$ NMR (151 MHz, $CDCl_3$) δ: 153.7, 61.9, 57.5, 32.8, 31.7, 25.3, 24.8, 23.5, 23.0.

3. Deuteration of Losartan Potassium

Standard Conditions (Table 1, Entry 3, NMR analysis)

To a mixture of losartan potassium (34.6 mg, 0.075 mmol, **1**-K^+) and **Ir-1** (2.0 mg, 0.0038 mmol) in a test tube equipped with a screw cap were added 2-propanol (3.0 μL, 0.038 mmol), D_2O (693 μL), and NaOD (in D_2O, 0.20 M, 57.0 μL). After stirring at 100 °C for 5 h in a closed system, the reaction mixture was directly analyzed by ^1H NMR.

Table S1. Further Optimization of Deuteration of Losartan Potassium (1-K^+)

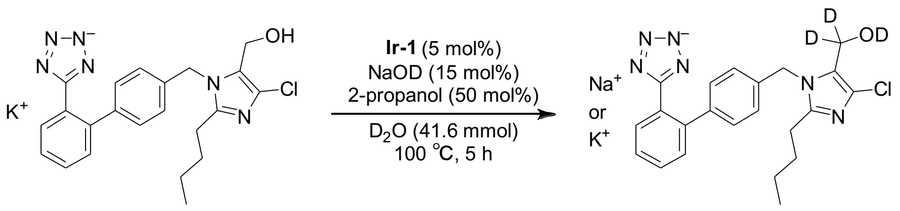

entry	Changes from the above scheme	results
1	NaOD (5 mol%)	18% D
2	NaOD (10 mol%)	37% D
3	NaOD (30 mol%)	60% D
4	NaOH in D_2O instead of NaOD	43% D
5	NaOH in D_2O instead of NaOD, 24 h	89% D
6	KO^{t}Bu instead of NaOD	47% D
7	CH_3OH instead of 2-propanol	37% D
8	1-phenylethanol instead of 2-propanol	34% D
9	acetone instead of 2-propanol	45% D
10	2-propanol (25 mol%)	45% D
11	**Ir-1** (2.5 mol%)	24% D
12	**1**-K^+ (0.2 M)	58% D
13	**1**-K^+ (0.05 M)	28% D
14	**1**-K^+ (0.2 M), 80 °C	27% D
15	without 2-propanol, 130 °C, 24 h	96% D
16	**1**-K^+(0.75 mmol), without 2-propanol, 130 °C	Complex mixture

1g-Scale (Table 1, Entry 18, Isolation)

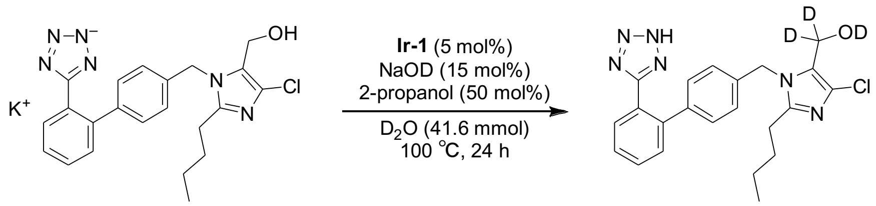

1-K+ [2H]1

Losartan-*d₂*: To a mixture of losartan potassium (1.00 g, 2.17 mmol, **1**-K$^+$) and **Ir-1** (58 mg, 0.015 mmol) in a test tube equipped with a screw cap and a stirring bar were added 2-propanol (82.5 μL, 1.09 mmol), D_2O (2.7 mL), and NaOD (in D_2O, 0.20 M, 1.63 mL). After stirring at 100 °C for 24 h in a closed system, the reaction mixture was neutralized with aq H_2SO_4 (0.1 M, *ca.* 10 mL) to pH 7.0 (until the mixture turned into a suspension). The iridium catalyst was separated by extraction with CH_2Cl_2 (15 mL × 5). The aqueous layer was acidified with aq H_2SO_4 (0.1 M, *ca.* 10 mL) to pH 3.0−4.0, put for 15 min, and extracted with EtOAc (50 mL × 3) and dried over Na_2SO_4. After filtration, the filtrate was concentrated under reduced pressure to afford losartan-d_2 (888 mg, 2.09 mmol, 96% yield, 96% D). White solid. ^{1}H NMR (600 MHz, CD_3OD) δ: 7.67 (2H, t, J = 7.6 Hz), 7.56 (2H, dd, J = 15.1, 6.9 Hz), 7.12 (2H, d, J = 8.3 Hz), 7.03 (2H, d, J = 9.0 Hz), 5.31 (2H, s), 4.45 (0.07H, s), 2.57 (2H, t, J = 7.2 Hz), 1.55−1.50 (2H, m), 1.30 (2H, td, J = 14.5, 8.0 Hz), 0.87 (3H, t, J = 7.2 Hz); ^{13}C{^{1}H} NMR (151 MHz, DMSO-d_6) *δ*: 147.4, 141.0, 138.4, 136.2, 131.1, 130.6, 129.1, 127.9, 126.3, 125.6, 125.3, 123.5, 51.0–50.5 (m), 29.0, 25.8, 21.7, 13.6; HRMS (ESI) *m/z*: [M−H]$^-$ calcd for $C_{22}H_{20}D_2ClN_6O^-$ 423.1675, found 423.1482.

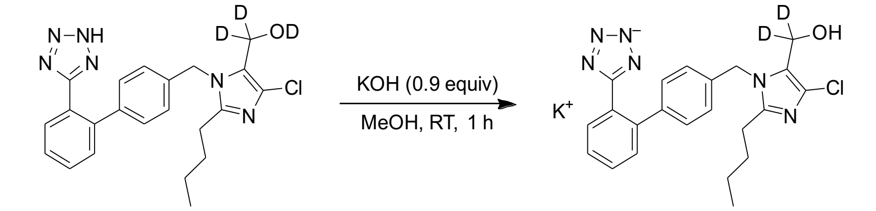

[2H]1 [2H]1-K+

Losartan Potassium-*d₂*: To a solution of losartan-d_2 (**[^{2}H]1**, 863 mg, 2.03 mmol) in CH_3OH (14 mL) was added KOH (1.83 mmol, 0.5 M in 3.66 mL CH_3OH). After stirring for 1 h, the reaction mixture was evaporated under reduced pressure. Reprecipitation in dry acetone gave pure losartan potassium-d_2 (**[^{2}H]1**-K$^+$, 682.7 mg, 1.47 mmol, 72% yield, 96% D). White solid. ^{1}H NMR (600 MHz, DMSO-d_6) δ: 7.54 (1H, t, J = 4.5 Hz), 7.36 (2H, tt, J = 10.3, 3.4 Hz), 7.28 (1H, dd, J = 7.2, 1.7 Hz), 7.10 (2H, d, J = 8.3 Hz), 6.91 (2H, d, J = 8.3 Hz), 5.26 (1H, s), 5.21 (2H, s), 4.31 (0.07H, s), 1.51−1.46 (2H, m), 1.26 (2H, td, J = 14.8, 7.3 Hz), 0.81 (3H, t, J = 7.2 Hz). ^{13}C{^{1}H} NMR (151 MHz, DMSO-d_6) *δ*: 160.7, 147.4, 141.2, 139.8, 134.6, 132.6, 130.5, 130.0, 129.4, 127.3, 126.7, 125.6, 125.3, 125.3, 50.9–50.6 (m), 46.5, 29.1, 25.8, 21.7, 13.7; HRMS (ESI) *m/z*: [M+H]$^+$ calcd for $C_{22}H_{21}D_2ClKN_6O^+$ 463.1379, found 463.1316.

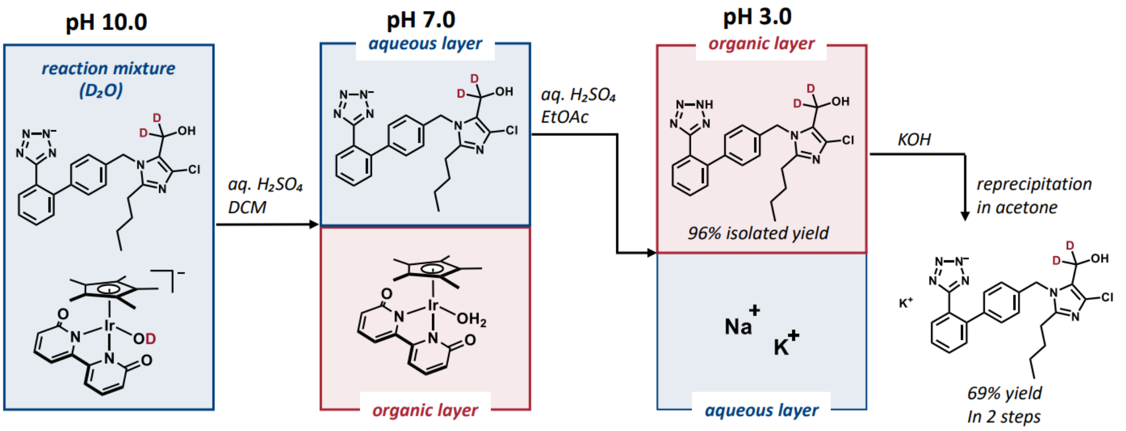

Fig. S1. Purification of losartan potassium-d_2.

4. Catalytic H/D Exchange Reaction

General Procedures

Conditions I: To a mixture of alcohol (0.30 mmol) and **Ir-1** (1.6 mg, 0.003 mmol) in a screw cap NMR tube were added 2-propanol (2.3 μL, 0.03 mmol), D_2O (555 μL), NaOD (in D_2O, 0.2 M, 45 μL), and 1,4-dioxane (12.8 μL, 0.15 mmol). The mixture was heated at 80 °C in a pre-heated oil bath in a closed system. The reaction progress was monitored by ^{1}H NMR until the H/D ratio reached the saturation point.

Conditions II: To a mixture of alcohol (0.30 mmol) and **Ir-1** (1.6 mg, 0.003 mmol) in a screw cap NMR tube were added 2-propanol (2.3 μL, 0.03 mmol), CD_3OD (300 μL), D_2O (255 μL) and NaOD (in D_2O, 0.2 M, 45 μL). The mixture was heated at 80 °C in a pre-heated oil bath in a closed system. The reaction progress was monitored by ^{1}H NMR until reaching the saturation point. The reaction mixture was acidified with aq. H_2SO_4 (0.1 M, a few drops) to pH 5.0, extracted with Et_2O (8 mL × 3), then dried over Na_2SO_4. After filtration, the filtrate was concentrated under reduced pressure. The residue was purified to afford deuterated alcohol.

Conditions III: To a mixture of alcohol (0.30 mmol) and **Ir-1** (1.6 mg, 0.003 mmol) in a screw cap NMR tube were added 2-propanol (2.3 μL, 0.03 mmol), CD_3OD (300 μL) and D_2O (300 μL). The mixture was heated at 80 °C in a pre-heated oil bath in a closed system. The reaction progress was monitored by ^{1}H NMR until reaching the saturation point. The reaction mixture was acidified with aq. H_2SO_4 (0.1 M, a few drops) to pH 5.0, extracted with Et_2O (8 mL × 3), then dried over Na_2SO_4. After filtration, the filtrate was concentrated under reduced pressure. The residue was purified to afford deuterated alcohol.

1-propanol-d_5 ([^{2}H]2)

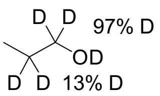

Conditions I. Substrate : 1-propanol (18.14 mg, 0.302 mmol, **2**). 1,4-dioxane (13.19 mg, 0.150 mmol) as an internal standard (94% NMR yield, α: 97% D, β: 13% D). ^{1}H NMR (600 MHz, D_2O) δ: 3.48 (0.05H, t, J = 6.2 Hz), 1.50−1.45 (1.73H, m), 0.84 (3H, t, J = 6.9 Hz); ^{13}C{^{1}H} NMR (151 MHz, D_2O) δ: 64.0–63.2 (m), 25.1–24.5 (m), 10.2.

1-butanol-d_5 ([^{2}H]3)

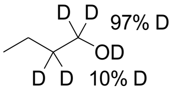

Conditions I. Substrate: 1-butanol (22.14 mg, 0.299 mmol, **3**). 1,4-dioxane (13.51 mg, 0.153 mmol) as an internal standard (>99% NMR yield, α: 97% D, β: 10% D). ^{1}H NMR (600 MHz, D_2O) δ: 3.71 (4H, s), 3.53 (0.05H, t, J = 6.5 Hz), 1.46 (1.80H, t, J = 7.6 Hz), 1.29 (2H, td, J = 14.8, 7.6 Hz), 0.85 (3H, t, J = 7.2 Hz); ^{13}C{^{1}H} NMR (151 MHz, D_2O) δ: 61.2−61.9 (m), 34.0–33.3 (m), 19.0, 13.7.

4-d_4 ([^{2}H]4)

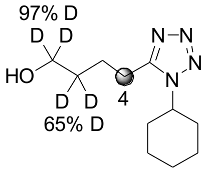

Conditions II. Substrate: **4** (13.0 mg, 0.058 mmol). **Ir-1**: 1.6 mg, 5 mol%. NaOD (in D_2O, 0.2 M, 15 mol%), 0.1 M, 100 °C. After the H/D ratio reached saturation point, the reaction mixture was acidified with aq H_2SO_4 (0.1 M, 2 drops) to pH 5.0 and extracted with $CHCl_3$ (8 mL × 3). The organic layers were combined and dried over Na_2SO_4. After filtration, the filtrate was concentrated under reduced pressure. The residue was purified by silica gel column chromatography ($CHCl_3$/CH_3OH = 20:1) to afford the deuterated alcohol **[^{2}H]4**. A colorless liquid (12.0 mg, 91%). ^{1}H NMR (600 MHz, $CDCl_3$) δ: 4.17−4.12 (1H, m), 3.69 (0.05H, s), 2.89 (1.91H, t, J = 7.6 Hz), 2.04−1.90 (8H, m), 1.80−1.75 (1H, m), 1.69−1.65 (0.69H, m), 1.50−1.40 (2H ,m), 1.40−1.30 (1H, m); ^{13}C{^{1}H} NMR (151 MHz, $CDCl_3$) δ: 153.8, 61.1−60.8 (m), 57.5, 32.8, 31.5−30.6 (m), 25.2, 24.8, 23.4−22.9 (m, 2C); HRMS (ESI) m/z: [M+H]$^+$ calcd for $C_{11}H_{17}D_4N_4O_4$ 229.1961, found 229.1853.

1,5-pentanediol-d_6 ([^{2}H]5)

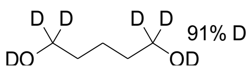

Conditions I. Substrate: 1,5-pentanediol (31.79 mg, 0.305 mmol, **5**). 1,4-dioxane (13.41 mg, 0.152 mmol) as an internal standard (>99% NMR yield, α: 91% D, β: <3% D). ^{1}H NMR (600 MHz, D_2O) δ: 3.59–3.52 (0.34H, m), 1.54–1.48 (4H, m), 1.35–1.29 (2H, m); ^{13}C{^{1}H} NMR (151 MHz, D_2O) δ: 62.3–61.3 (m), 31.7, 22.0.

benzyl alcohol-d_2 ([^{2}H]6)

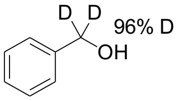

Conditions II. Substrate: benzyl alcohol (31.0 µL, 0.30 mmol, **6**). Purified by PTLC (SiO_2, hexane/EtOAc = 3:2). A colorless liquid (30.2 mg, 91%, 96% D): ^{1}H NMR (600 MHz, $CDCl_3$) δ: 7.40–7.32 (4H, m), 7.32–7.28 (1H, m), 4.67 (0.08H, s), 1.76 (1H, brs); ^{13}C{^{1}H} NMR (151 MHz, $CDCl_3$) δ:140.7, 128.6, 127.7, 127.1, 65.0–64.4 (m).

2-methylbenzyl alcohol-d_2 ([^{2}H]7)

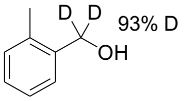

Conditions II. Substrate: 2-methylbenzyl alcohol (36.7 mg, 0.30 mmol, **7**). Purified by column chromatography (SiO_2, hexane/Et_2O = 1:1). A white solid (36.6 mg, 98%, 93% D): ^{1}H NMR (600 MHz, $CDCl_3$) δ: 7.36–7.34 (1H, m), 7.23–7.18 (3H, m), 4.68 (0.14H, s), 2.36 (3H, s), 1.56 (1H, brs); ^{13}C{^{1}H} NMR (151 MHz, $CDCl_3$) δ: 138.5, 136.2, 130.4, 127.9, 127.6, 126.1, 63.4–62.8 (m), 18.7.

3-methylbenzyl alcohol-d_2 ([^{2}H]8)

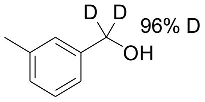

Conditions II. Substrate: 3-methylbenzyl alcohol (36.0 µL, 0.30 mmol, **8**). Purified by column chromatography (SiO_2, hexane/Et_2O = 1:1). A yellow liquid (33.8 mg, 91%, 96% D) ^{1}H NMR (600 MHz, CD_3OD) δ: 7.20 (1H, t, J = 7.6 Hz), 7.16 (1H, s), 7.12 (1H, d, J = 7.6 Hz), 7.07 (1H, d, J = 7.6 Hz), 4.54 (0.07H, s), 2.33 (3H, s); ^{3}C{^{1}H} NMR (151 MHz, CD_3OD) δ: 142.5, 139.0, 129.3, 129.0, 128.7, 125.2, 65.1–64.3 (m), 21.4.

4-methylbenzyl alcohol-d_2 ([^{2}H]9)

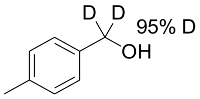

Conditions II. Substrate: 4-methylbenzyl alcohol (36.7 mg, 0.30 mmol, **9**). Purified by column chromatography (SiO_2, hexane/Et_2O = 1:1). A white solid (34.4 mg, 92%, 95% D): ^{1}H NMR (600 MHz, CD_3OD) δ: 7.22 (2H, d, J = 9.0 Hz), 7.14 (2H, d, J = 8.3 Hz), 4.52 (0.10H, s), 2.31 (3H, s); ^{13}C{^{1}H} NMR (151 MHz, CD_3OD) δ:139.5, 138.0, 130.0, 128.2, 64.9–64.3 (m), 21.2.

4-fluorobenzyl alcohol-d_2 ([^{2}H]10)

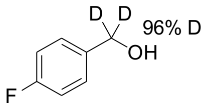

Conditions II. Substrate: 4-fluorobenzyl alcohol (32.7 µL, 0.30 mmol, **10**). Purified by column chromatography (SiO_2, hexane/Et_2O = 2:1). A colorless liquid (43.0 mg, quant. 96% D). ^{1}H NMR (600 MHz, CD_3OD) δ: 7.36 (2H, t, J = 6.5 Hz), 7.07–7.04 (2H, m), 4.56 (0.09H, s); ^{13}C{^{1}H} NMR (151 MHz, CD_3OD) δ:163.6 (d, J_{CF} = 244.6 Hz), 138.7, 130.0 (J_{CF} = 7.6 Hz), 116.0 (dd, J_{CF} = 21.1, 4.5 Hz) 63.8 (quint, J_{CD} = 21.1 Hz, CD$_2$) and 64.1 (t, J_{CD} = 21.9 Hz, CHD).

4-bromobenzyl alcohol-d_2 ([^{2}H]11)

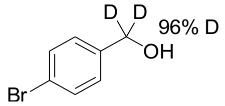

Conditions II. Substrate: 4-bromobenzyl alcohol (56.1 mg, 0.30 mmol, **11**). Purified by column chromatography (SiO_2, hexane/Et_2O = 1:1). A white solid (54.9 mg, 97%, 96% D). ^{1}H NMR (600 MHz, CD_3OD) δ: 7.48 (2H, dd, J = 6.5, 1.7 Hz), 7.27 (2H, dd, J = 6.5, 1.7 Hz), 4.54 (0.09H, s); ^{13}C{^{1}H} NMR (151 MHz, CD_3OD) δ: 142.0, 132.4, 129.9, 121.9, 63.7 (quint, J_{CD} = 21.5 Hz, CD$_2$) and 64.1 (t, J_{CD} = 21.9 Hz, CHD).

4-trifluoromethylbenzyl alcohol-d_2 ([^{2}H]12)

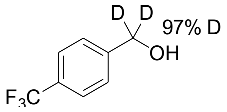

Conditions II. Substrate: 4-trifluoromethylbenzyl alcohol (41.0 µL, 0.30 mmol, **12**). Purified by column chromatography (SiO_2, hexane/Et_2O = 1:1). A colorless liquid (48.5 mg, 91%, 97% D). ^{1}H NMR (600 MHz, $CDCl_3$) δ: 7.60 (2H, d, J = 8.3 Hz), 7.45 (2H, d, J = 8.3 Hz), 4.72 (0.07H, s), 2.18 (1H, s); ^{13}C{^{1}H} NMR (151 MHz, $CDCl_3$) δ:144.6, 129.8 (q, J_{CF} = 32.2 Hz), 126.9, 125.5 (q, J_{CF} = 4.5 Hz), 124.2(q, J_{CF} = 272.3 Hz), 63.8 (quint, J_{CD} = 21.9 Hz, CD$_2$) and 64.1 (t, 21.9 Hz, CHD).

4-nitrobenzyl alcohol-d_2 ([^{2}H]13)

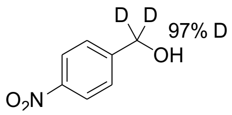

Conditions II. Substrate: 4-nitrobenzyl alcohol (45.9 mg, 0.30 mmol, **13**). Purified by PTLC (SiO_2, hexane/EtOAc = 3:2). A pale-yellow solid (36.8 mg, 79%, 97% D). ^{1}H NMR (600 MHz, $CDCl_3$) δ: 8.24 (2H, d, J = 8.3 Hz), 7.55 (2H, d, J = 8.3 Hz), 4.83 (0.07H, s), 1.93 (1H, s); ^{13}C{^{1}H} NMR (151 MHz, $CDCl_3$) δ:148.0, 147.3, 127.1, 123.8, 63.5–62.2 (m).

4-pyridinemethanol-*d*$_2$ ([^{2}H]14)

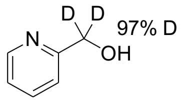

Conditions I. Substrate: 4-pyridinemethanol (28.9 μL, 0.30 mmol, **14**). The reaction was conducted w/o 1,4-dioxane. After the H/D ratio reached saturation point, the reaction mixture was acidified with aq H_2SO_4 (0.1 M, 2 drops) to pH 5.0 and extracted with Et_2O (8 mL × 3). The organic layers were combined and dried over Na_2SO_4. After filtration, the filtrate was concentrated under reduced pressure. The residue was purified by silica gel column chromatography (hexane/EtOAc = 2:1 to 2:3) to afford the deuterated alcohol **[^{2}H]12**. A colorless liquid (27.0 mg, 81%, 97% D). ^{1}H NMR (600 MHz, CD_3OD) *δ*: 8.46 (1H, d, J = 4.8 Hz), 7.86 (1H, td, J = 7.7, 1.8 Hz), 7.57 (1H, d, J = 7.6 Hz), 7.32−7.30 (1H, m), 4.67 (0.07H, s); ^{13}C{^{1}H} NMR (151 MHz, CD_3OD) δ: 162.2, 149.3, 138.9, 123.8, 122.2, 65.1−64.6 (m).

2-butanol-*d*$_2$ ([^{2}H]15)

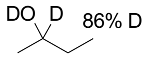

Conditions III. Substrate: 2-butanol (22.11 mg, 0.298 mmol, **15**). **Ir-1**: 3.2 mg, 2.0 mol%. Solvent: CD_3OD (600 μL). After reaching saturation point, maleic acid (17.39 mg, 0.150 mmol) was added to the reaction mixture as an internal standard (94% NMR yield, α: 86%D, β: <3%D). ^{1}H NMR (600 MHz, CD_3OD) δ: 3.67–3.61 (0.14H, m), 1.49–1.38 (2H, m), 1.15–1.12 (4H, m), 0.91 (3H, t, J = 7.6 Hz); ^{13}C{^{1}H} NMR (151 MHz, CD_3OD) δ: 70.0 (*C*H) and 69.5 (t, J_{CD} = 21.1 Hz, *C*D), 32.7, 25.1, 22.8, 10.4.

cyclohexanol-*d*$_2$ ([^{2}H]16)

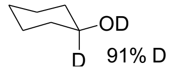

Conditions III. Substrate: cyclohexanol (30.44 mg, 0.304 mmol, **16**). **Ir-1**: 3.2 mg, 2.0 mol%. Solvent: CD_3OD (600 μL). 1,4-dioxane (13.50 mg, 0.153 mmol) as an internal standard (>99% NMR yield, α: 91%D, β: <3%D). ^{1}H NMR (600 MHz, CD_3OD) δ: 3.55–3.50 (0.09H, m), 1.90–1.81 (2H, m), 1.79–1.71 (2H, m), 1.58–1.52 (1H, m), 1.35–1.12 (5 H, m); ^{13}C{^{1}H} NMR (151 MHz, CD_3OD) δ: 70.9 (*C*H) and 70.4 (t, J_{CD} = 21.1 Hz, *C*D), 36.2, 26.7, 25.3.

1-phenylethanol-*d*$_1$ ([^{2}H]17)

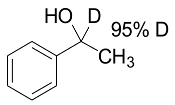

Conditions III. Substrate: 1-phenylethanol (36.3 μL, 0.30 mmol, **17**). Purified by column chromatography (SiO_2, hexane/Et_2O = 5:1) A colorless liquid (31.0 mg, 84%, 95% D). ^{1}H NMR (600 MHz, DMSO-*d*$_6$) δ: 7.34–7.29 (4H, m), 7.22–7.18 (1H, m), 5.11 (1H, s),

4.71 (0.05H, s), 1.30 (3H, s); $^{13}C\{^1H\}$ NMR (151 MHz, DMSO-d_6) δ: 147.4, 128.0, 126.5, 125.3, 68.1 (*C*H) and 67.6 (t, J_{CD} = 21.1 Hz, *C*D), 25.9.

1-(4-methylphenyl)ethanol-d_1 ([^{2}H]18)

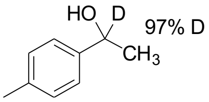

Conditions III. Substrate: 1-(4-methylphenyl)ethanol (41.3 μL, 0.30 mmol, **18**). Purified by column chromatography (SiO_2, hexane/Et_2O = 1:1). A colorless liquid (41.7 mg, quant. 97% D). ^{1}H NMR (500 MHz, DMSO-d_6) δ: 7.21 (2H, d, J = 8.0 Hz), 7.10 (2H, d, J = 8.6 Hz), 5.04 (1H, s), 4.67 (0.03H, s), 2.27 (3H, s), 1.28 (3H, s); $^{13}C\{^1H\}$ NMR (151 MHz, DMSO-d_6) δ: 144.4, 135.4, 128.5, 125.3, 67.9 (*C*H) and 67.5 (t, J_{CD} = 23.4 Hz, *C*D), 25.9, 20.7.

1-(4-bromophenyl)ethanol-d_1 ([^{2}H]19)

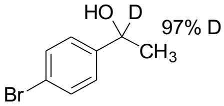

Conditions III. Substrate: 1-(4-bromophenyl)ethanol (41.3 μL, 0.30 mmol, **19**). Purified by PTLC (SiO_2, hexane/Et_2O = 1:1). A colorless liquid (59.6 mg, 98%, 97% D). ^{1}H NMR (600 MHz, DMSO-d_6) δ: 7.49 (2H, d, J = 8.3 Hz), 7.32–7.30 (2H, m), 5.24 (1H, s), 4.68 (0.03H, s), 1.29 (3H, s); $^{13}C\{^1H\}$ NMR (151 MHz, DMSO-d_6) δ: 146.8, 130.9, 127.6, 119.4, 67.5 (*C*H) and 67.1 (t, J_{CD} = 21.9 Hz, *C*D), 25.7.

α-cyclopropylbenzyl alcohol-d_1 ([^{2}H]20)

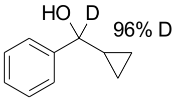

Conditions III. Substrate: α-cyclopropylbenzyl alcohol (42.8 μL, 0.30 mmol, **20**). Washed with brine and purified by PTLC (SiO_2, hexane/Et_2O = 1:1). A colorless liquid (42.2 mg, 90%, 96% D). ^{1}H NMR (600 MHz, $CDCl_3$) δ: 7.42 (2H, d, J = 6.9 Hz), 7.35 (2H, td, J = 7.6, 3.4 Hz), 7.31–7.25 (1H, m), 4.00 (0.04, d, J = 8.3 Hz), 2.10–2.05 (1H, m), 1.23–1.19 (1H, m), 0.65–0.61 (1H, m), 0.56–0.52 (1H, m), 0.48–0.44 (1H, m), 0.39–0.34 (1H, m); $^{13}C\{^1H\}$ NMR (151 MHz, $CDCl_3$) δ: 143.7, 128.4, 127.6, 126.0, 78.6 (*C*H) and 78.1(t, J_{CD} = 22.7 Hz, *C*D), 19.1, 3.6, 2.8.

benzhydrol-d_1 ([^{2}H]21)

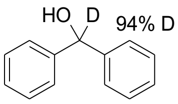

Conditions III. Substrate: benzhydrol (55.3 mg, 0.30 mmol, **21**). Solvent: CD_3OD (600 μL). Purified by column chromatography (SiO_2, hexane/Et_2O = 4:1 to 2:1). A white solid (54.5 mg, 98%, 94% D). ^{1}H NMR (600 MHz, CD_3OD) δ: 7.36 (4H, d, J = 7.6 Hz), 7.35–

7.25 (4H, m), 7.22 (2H, t, $J = 6.9$ Hz), 5.77 (0.06H, s); $^{13}C\{^1H\}$ NMR (151 MHz, CD_3OD) δ: 145.9, 129.3, 129.3, 128.2, 127.7, 76.9 (*C*H) and 76.5 (t, $J_{CD} = 21.1$ Hz, *C*D).

2,4-dichlorobenzyl alcohol (rapidosept)-*d*₂ ([^{2}H]22)

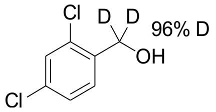

Conditions II. Substrate: 2,4-dichlorobenzyl alcohol (rapidosept) (53.1 mg, 0.30 mmol, **22**). **Ir-1** (3.2 mg, 2 mol%). NaOD (in D_2O, 0.2 M, 6 mol%). Purified by column chromatography (SiO_2, hexane/Et_2O = 3:1). A white solid (46.5 mg, 87%, 96% D): ^1H NMR (600 MHz, CD_3OD) δ: 7.53–7.51 (1H, m), 7.42–7.40 (1H, m), 7.36–7.33 (1H, m), 4.64 (0.08H, s); $^{13}C\{^1H\}$ NMR (151 MHz, $CDCl_3$) δ:136.7, 133.9, 133.3, 129.5, 129.2, 127.3, 62.1–61.6 (m).

tyrosol-*d*₄ ([^{2}H]23)

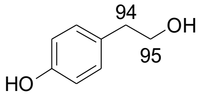

Conditions I. Substrate: 2-(4-hydroxyphenyl)ethanol (tyrosol) (41.5 mg, 0.30 mmol, **23**). NaOD (in D_2O, 1.0 M, 103 mol%). Purified by PLC (SiO_2, hexane/EtOAc = 1:2). A white solid (36.9 mg, 87%): ^1H NMR (600 MHz, D_2O) δ: 7.13−7.11 (2H, m), 6.81−6.79 (2H, m), 3.69−3.72 (0.09H, m), 2.73−2.68 (0.11H, m). $^{13}C\{^1H\}$ NMR (151 MHz, D_2O) δ: 154.3, 131.4, 130.8, 115.9, 63.0−62.3 (m), 37.0−36.3 (m).

guaiacol glycerol ether-*d*₄ ([^{2}H]24)

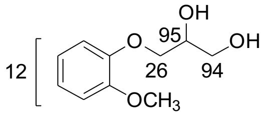

Conditions I. Substrate: guaiacol glycerol ether (59.5 mg, 0.30 mmol, **24**). **Ir-1** (8.0 mg, 0.015 mmol), NaOD (in D_2O, 0.2 M, 225 μL), 2-propanol (2.3 μL, 0.03 mmol), D_2O (375 μL), 100 °C. After reaching the saturation point, the reaction mixture was extracted with $CHCl_3$ then dried over Na_2SO_4. After filtration, the filtrate was concentrated under reduced pressure. The residue was purified by silica gel column chromatography (hexane/EtOAc = 1:5) to afford **[^{2}H]24** as a white solid (49.4 mg, 75%). ^1H NMR (600 MHz, $CDCl_3$) δ: 6.99–6.89 (3.51H, m), 4.16−4.12 (0.74H, m), 4.08−4.03 (0.79H, m), 3.86 (3H, s), 3.80 (0.05H, s), 3.76 (0.04H, s); $^{13}C\{^1H\}$ NMR (151 MHz, $CDCl_3$) δ: 149.6, 147.9, 122.2, 121,1, 114.6, 111.7, 72.2, 69.2–69.8 (m), 62.9–63.2 (m), 55.8; HRMS (ESI) *m/z*: $[M+Na]^+$ calcd for $C_{10}H_{10}D_4NaO_4$ 225.1035, found 225.0845.

diprophylline-d_6 ([^{2}H]25)

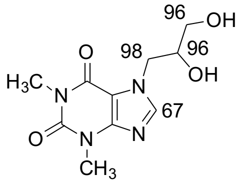

Conditions I. Substrate : diprophylline (152.8 mg, 0.60 mmol, **25**). **Ir-1** (16.0 mg, 0.03 mmol), NaOD (in D_2O, 0.2 M, 450 µL), 2-propanol (4.6 µL, 0.06 mmol), D_2O (750 µL), 100 °C. After reaching the saturation point, the reaction mixture was washed with CH_2Cl_2 then evaporated. The residue was reprecipitated and washed with CH_3OH to afford **[^{2}H]25** as a white solid (56.2 mg, 37%). ^{1}H NMR (600 MHz, D_2O) δ: 7.93 (0.33H, s), 4.42 (0.04H, s), 4.17 (0.04H, s), 3.99 (0.04H, s), 3.63 (0.04H, s), 3.45 (3H, s), 3.27 (3H, s); ^{13}C{^{1}H} NMR (151 MHz, D_2O) δ: 156.1, 152.7, 148.7, 143.9, 107.2, 69.8 (t, J_{CD} = 19.6 Hz), 62.1, 49.0, 30.0, 28.3.; HRMS (ESI) *m/z*: [M+Na]$^+$ calcd for $C_{10}H_8D_6N_4NaO_4$ 283.1289, found 283.1061.

(4-ethynyl)phenylmethanol-d_1 ([^{2}H]26)

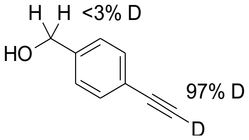

Conditions III. Substrate: (4-ethynyl)phenylmethanol (39.7 mg, 0.30 mmol, **26**). Purified by column chromatography (SiO_2, hexane/Et_2O = 1:1). A white solid (35.8 mg, 90%, 97% D). ^{1}H NMR (600 MHz, $CDCl_3$) δ: 7.49 (2H, d, J = 8.3 Hz), 7.33 (2H, d, J = 6.9 Hz), 4.71 (2H, d, J = 5.5 Hz), 3.07 (0.03H, s), 1.69 (1H, brs); ^{13}C{^{1}H} NMR (151 MHz, $CDCl_3$) δ: 141.5, 132.3, 126.7, 121.3, 83.0 (t, J_{CD} = 7.6 Hz), 76.7, 64.9.

1-phenylpropyn-1-ol-d_1 ([^{2}H]27)

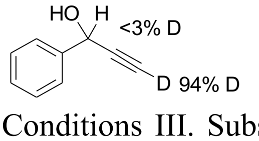

Conditions III. Substrate: 1-phenylpropyn-1-ol (36.5 µL, 0.30 mmol, **27**). Purified by column chromatography (SiO_2, hexane/Et_2O = 2:1 to 3:2). A white solid (35.4 mg, 89%, 94% D). ^{1}H NMR (600 MHz, $CDCl_3$) δ: 7.57–7.53 (2H, m), 7.39 (2H, td, J = 7.6, 1.4 Hz), 7.35–7.31 (1H, m), 5.46 (1H, s), 2.67 (0.03H, s), 2.36 (1H, brs); ^{13}C{^{1}H} NMR (151 MHz, $CDCl_3$) δ: 140.0, 128.7, 128.6, 126.6, 83.4 (C≡CH) and 83.0 (t, J_{CD} = 7.6 Hz, C≡CD), 74.6 (t, J_{CD} = 38.5 Hz), 64.4.

5. Mechanistic Studies

Lactone formation from 1,2-benzenedimethanol

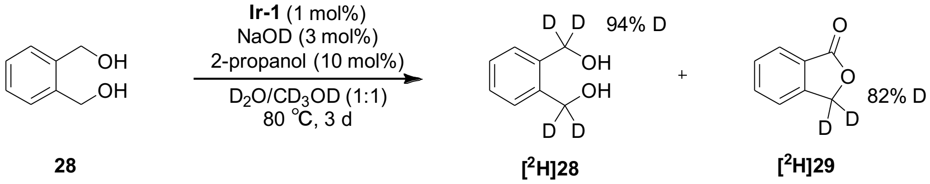

To a mixture of 1,2-benzenedimethanol (41.5 mg, 0.30 mmol, **28**) and **Ir-1** (1.6 mg, 0.003 mmol) in a screw cap NMR tube were added 2-propanol (2.3 μL, 0.03 mmol), CD_3OD (300 μL), D_2O (255 μL) and NaOD (in D_2O, 0.2 M, 45 μL). The tube was heated at 80 °C in a pre-heated oil bath in a closed system for 3 days. The reaction mixture was extracted with Et_2O (8 mL × 3), washed with brine, and dried over Na_2SO_4. After filtration, the filtrate was concentrated under reduced pressure. The residue was purified by PTLC (hexane/Et_2O = 1:1) to afford **[^{2}H]28** (29.0 mg, 0.204 mmol, 68%, 94% D) and **[^{2}H]29** (4.1 mg, 0.031 mmol, 10%, 82% D).

[^{2}H]28, colorless oil. ^{1}H NMR (500 MHz, $CDCl_3$) δ: 7.38–7.31 (4H, m), 4.73 (0.23H, s), 2.85 (2H).

[^{2}H]29, colorless oil. ^{1}H NMR (500 MHz, $CDCl_3$) δ: 7.94 (1H, d, *J* = 7.7 Hz), 7.70 (1H, td, *J* = 7.5, 1.1 Hz), 7.57–7.50 (2H, m), 5.33 (0.36H).

Deuteration of (*S*)-1-phenylethanol

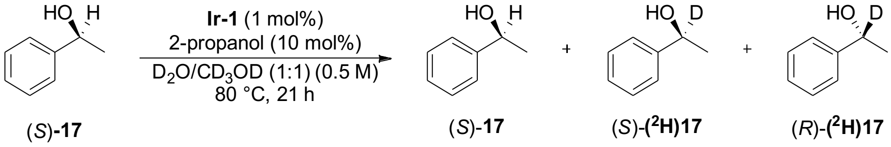

To a mixture of (*S*)-(-)-1-phenylethanol (36.3 μL, 0.30 mmol, *S/R* = 99.2:0.8, **(*S*)-17**) and **Ir-1** (1.6 mg, 0.003 mmol) in a screw cap NMR tube were added 2-propanol (0.03mmol, 2.3 μL), CD_3OD (300 μL) and D_2O (300 μL). The mixture was heated at 80 °C in a pre-heated oil bath in a closed system for 21 h, extracted with Et_2O (8 mL × 3), washed with brine, and dried over Na_2SO_4. After filtration, the filtrate was concentrated under reduced pressure. The residue was purified by silica gel column chromatography (hexane/Et_2O = 5:1 to 3:1) to afford **[^{2}H]17** (31.3 mg, 0.254 mmol, 85%, 95% D, *S/R* = 53.0:47.0). The enantiomeric ratio was determined by UPC analysis: Trefoil CEL1, 1.0 mL/min, 3% CH_3OH/CO_2, λ = 210−400 nm, t_R((*R*)-**17**) = 2.67 min, t_R((*S*)-**17**) = 3.25 min; t_R((*R*)-**[^{2}H]17**) = 2.81 min, t_R((*S*)- **[^{2}H]17**) = 3.37 min. Slight differences in the retention time for deuterated and non-deuterated materials were organized in a literature.[1]

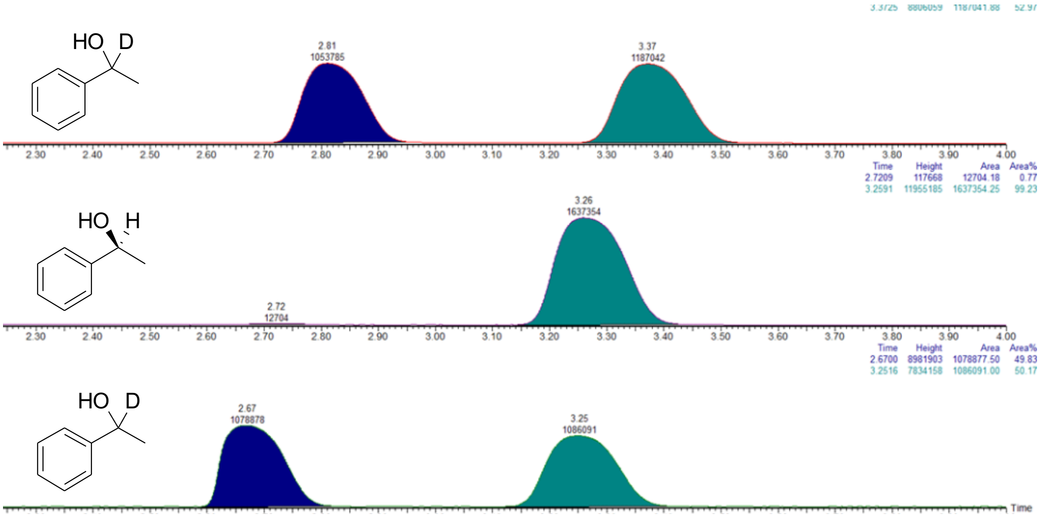

Synthesis of deuterated alcohols from 4-bromoacetophenone with 2-propanol

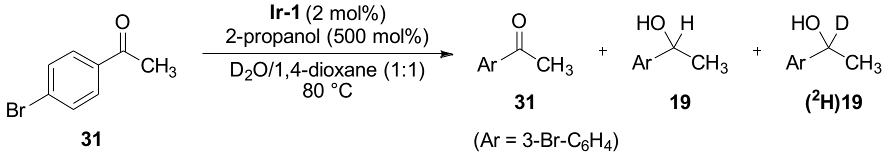

To a mixture of **31** (11.9 mg, 0.06 mmol), 2-propanol (23.1 µL, 0.30 mmol) and **Ir-1** (3.2 mg, 0.006 mmol) in a screw cap NMR tube were added 1,4-dioxane (300 µL) and D_2O (300 µL). The mixture was heated at 80 °C in a closed system in a pre-heated oil bath, acidified with aq H_2SO_4 (pH 5.0), extracted with Et_2O (8 mL × 3), and dried over Na_2SO_4. After filtration, the filtrate was concentrated under reduced pressure. The residue was analyzed by ^{1}H NMR (in acetone-d_6) without further purification.

6. NMR Studies

(a)

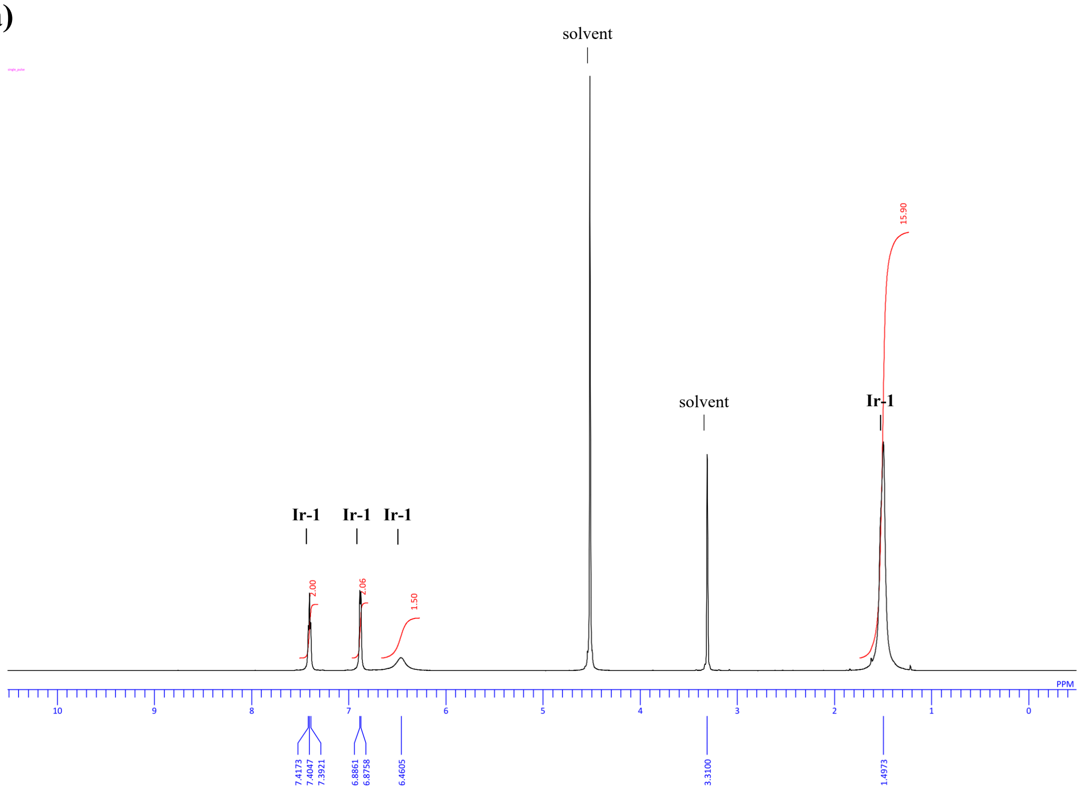

(b)

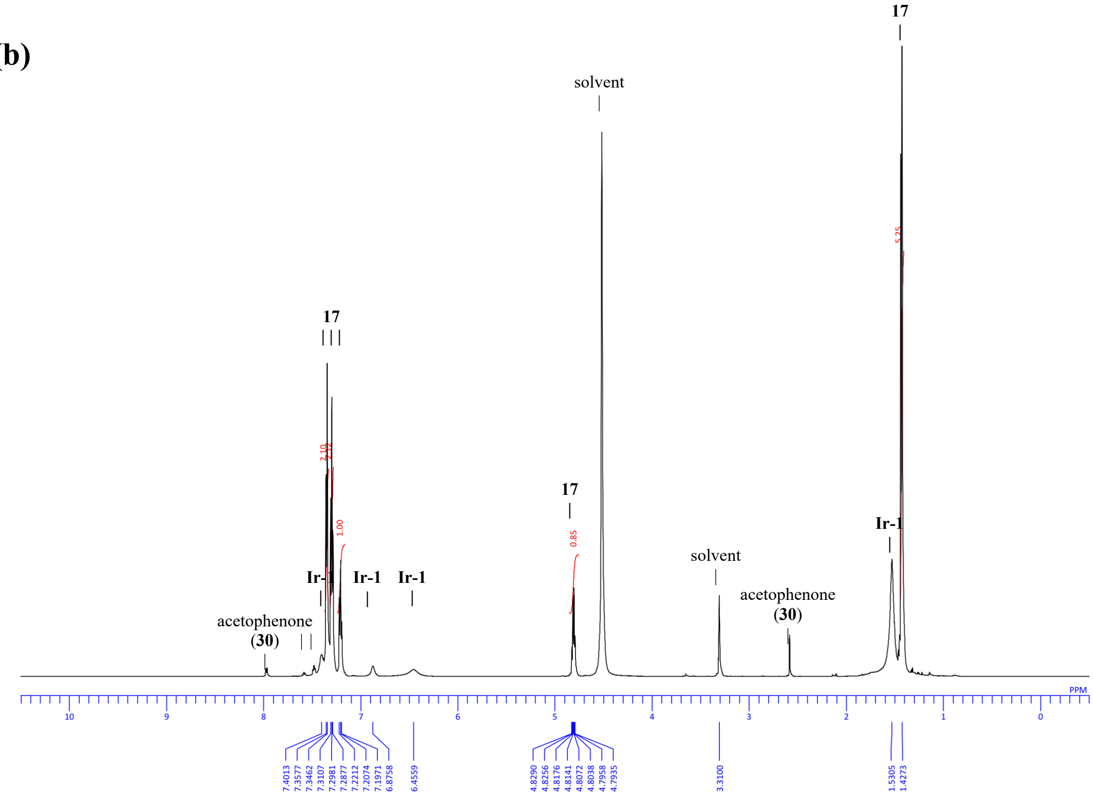

Fig. S2. ^{1}H NMR spectra at 60 °C of (a) **Ir-1** in CD_3OD and (b) a mixture of **17** and **Ir-1** (20 mol%) in CD_3OD after heating at 60 °C for 2 h.

7. Kinetic Experiments

To a screw cap NMR tube, **17** (36.3 µL, 0.3 mmol), **Ir-1** (1.6 mg, 0.015 mmol), D_2O (300 µL), and CD_3OD (300 µL) were added. The mixture was heated at 80 ºC in a closed system for a certain amount of time, extracted with Et_2O (8 mL × 1), and dried over Na_2SO_4. After filtration, the filtrate was condensed under reduced pressure. ^{1}H NMR (in $CDCl_3$) analysis of the reaction mixture indicated the deuteration of **17**.

A [**Ir-1**]$_0$ = 5.0 mM (1.0 mol%)

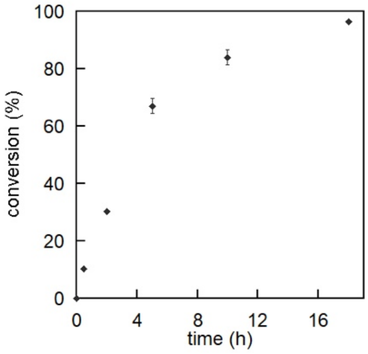

B [**Ir-1**]$_0$ = 7.5 mM (1.5 mol%)

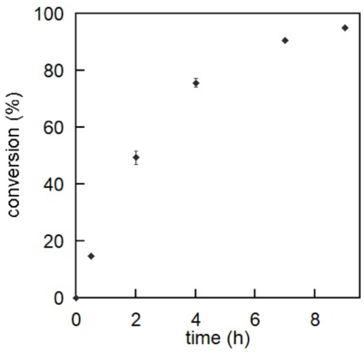

C [**Ir-1**]$_0$ = 10 mM (2.0 mol%)

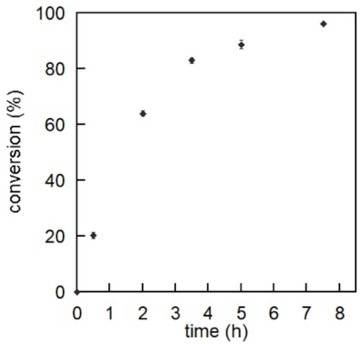

D [**Ir-1**]$_0$ = 5.0 mM (1.0 mol%)

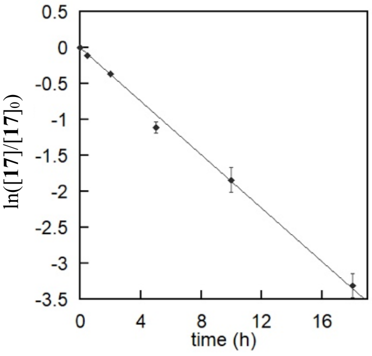

E [**Ir-1**]$_0$ = 7.5 mM (1.5 mol%)

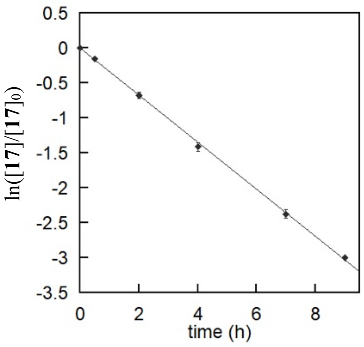

F [**Ir-1**]$_0$ = 10 mM (2.0 mol%)

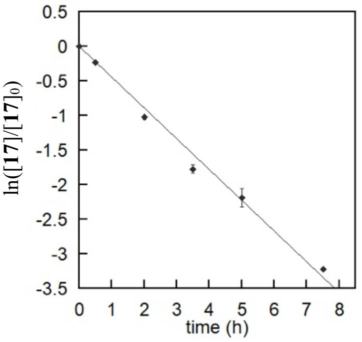

Fig. S3. Results of individual kinetic studies on the deuteration of **17**. Time conversion curve using (A) 1 mol%, (B) 1.5 mol%, (C) 2 mol% catalyst. Log plot using (D) 1 mol%, (E) 1.5 mol%, (F) 2 mol% catalyst. Average of 3 runs, with standard deviation.

8. The Effect of 2-Propanol

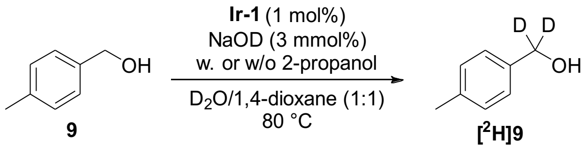

To a mixture of **9** (36.7 mg, 0.3 mmol), **Ir-1** (1.6 mg, 0.003 mmol) in a screw cap NMR tube were added 2-propanol, NaOD (in D_2O, 0.2 M, 45 μL), 1,4-dioxane (300 μL) and D_2O (255 μL). The mixture was heated at 80 °C in a closed system in a pre-heated oil bath, acidified with aq H_2SO_4 (pH 5.0), extracted with Et_2O (8 mL × 3), and dried over Na_2SO_4. After filtration, the filtrate was concentrated under reduced pressure. The residue was analyzed by ^{1}H NMR (in $CDCl_3$) without further purification.

	3 h	5 h	7 h
w/o 2-propanol	68% D	89% D	93% D
10 mol% 2-propanol	40% D	63% D	96% D
30 mol% 2-propanol	22% D	38% D	66% D

9. Computational Details

All the calculations were conducted with CAM-B3LYP+GD3 level of density functional theory calculation, in which Grimme's D3 version of empirical dispersion correction was included. The SDD effective core potential and the 6-31++G** electronic basis set were adopted for Ir and other atoms, respectively. The solvent effects (water) were included by the IEFPCM method. The normal mode analysis was performed to characterize the optimized structures and the Gibbs free energies were calculated at T = 353.15 K. We showed the most favorable structural isomers among we have tested for each pathway. To minimize errors in free energies, the energy difference in liberating (or incorporating) a molecule was treated as the formation of (or liberation from) a hydrate.

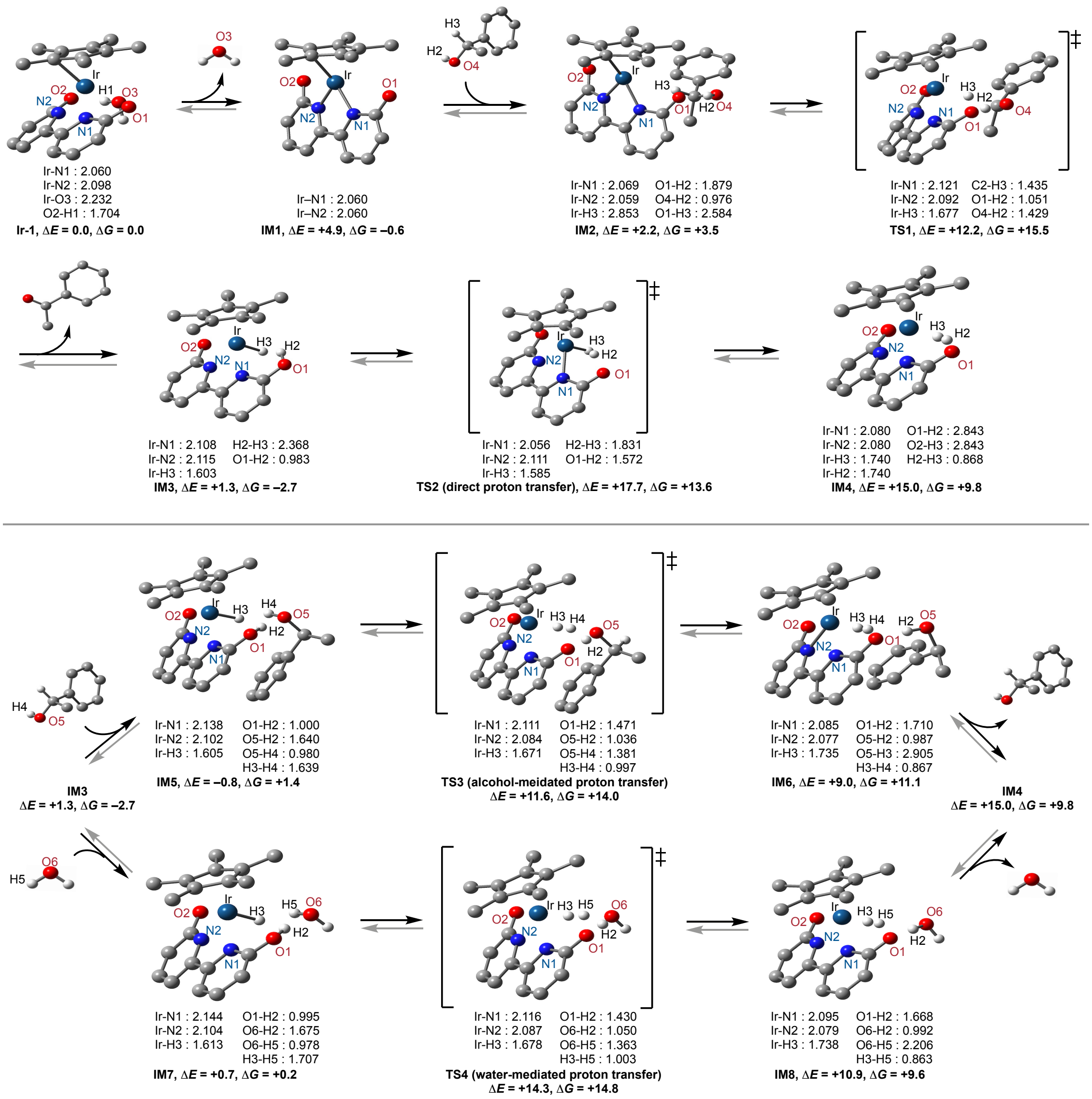

Fig. S4 DFT calculations on the proposed pathway at the CAM-B3LYP+GD3 level of theory using SDD and 6-31++G** basis sets for Ir and other atoms, respectively. Solvent effects (water) were included by IEFPCM method. Gibbs free energies in kcal mol^{-1} were calculated at $T = 353.15$ K.

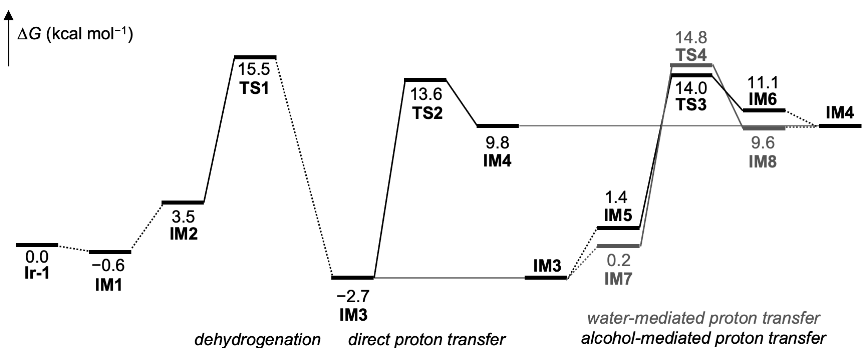

Fig. S5 Energy diagram at the CAM-B3LYP+GD3 level of theory using SDD and 6-31++G** basis sets for Ir and other atoms, respectively. Solvent effect (H_2O) by IEFPCM method; Gibbs free energies in kcal mol^{-1} at $T = 353.15$ K.

10. Cartesian Coordinates

Table S2. Cartesian coordinates (Å) of **Ir-1**

Atom	x	y	z
Ir	-0.471463	-0.020486	-0.210372
C	-1.684649	1.190996	1.152121
C	-1.171129	0.005426	1.801863
C	-1.769096	-1.146689	1.140372
C	-2.550052	-0.668664	0.055221
C	-2.492525	0.788852	0.050034
C	-1.411782	2.593033	1.582649
C	-0.347978	-0.031211	3.049227
C	-1.580642	-2.571779	1.545244
C	-3.317705	-1.498107	-0.920800
C	-3.231662	1.697080	-0.878608
H	-0.418323	2.684991	2.024774
H	-2.148117	2.882713	2.339630
H	-0.995743	-0.055137	3.931796
H	0.293881	0.848053	3.121892
H	-1.612442	-3.231029	0.678211
H	-2.938939	-2.520177	-0.951829
H	-2.651231	2.602979	-1.058250
H	-4.202611	1.972825	-0.453348
H	-3.248958	-1.079111	-1.926609
H	-2.368268	-2.863636	2.247340
H	-0.617794	-2.714823	2.038429
H	0.290431	-0.916090	3.072216
H	-1.472266	3.277929	0.738738
H	-3.410927	1.212970	-1.840423
H	-4.374726	-1.527421	-0.637274
O	0.134471	-2.984860	-1.215220
O	-0.077211	3.048137	-1.235027
N	1.212648	-1.253053	-0.210211
N	1.144447	1.305420	-0.389346
C	1.148393	-2.572635	-0.567305
C	2.261536	-3.390136	-0.203009
C	3.393524	-2.813285	0.318377
C	3.485784	-1.416436	0.474480
C	2.372607	-0.662315	0.175178
C	2.326993	0.816700	0.092529
C	3.399748	1.631418	0.364645
C	3.263764	3.011228	0.104542
C	2.106325	3.501616	-0.432627
C	0.990519	2.636665	-0.721238
H	2.195751	-4.454839	-0.394607
H	4.247331	-3.434713	0.571013
H	4.412045	-0.953121	0.786709
H	4.326769	1.225373	0.746581
H	4.091457	3.681013	0.318674
H	1.983243	4.554521	-0.659991

O	-0.411732	-0.643782	-2.353449
H	-0.271388	-1.617106	-2.147405
H	0.377744	-0.331817	-2.818506

Table S3. Cartesian coordinates (Å) of **IM1**

Atom	x	y	z
Ir	-0.365318	0.000098	-0.052775
C	-2.119244	1.175811	0.598317
C	-1.928302	-0.000210	1.413636
C	-2.119992	-1.175296	0.597291
C	-2.304744	-0.732177	-0.736962
C	-2.304413	0.734049	-0.736304
C	-2.194053	2.568380	1.130613
C	-1.712127	-0.000989	2.888280
C	-2.196186	-2.568255	1.128407
C	-2.546792	-1.594758	-1.928259
C	-2.546413	1.597471	-1.927000
H	-1.359368	2.782648	1.800631
H	-3.124745	2.681262	1.697247
H	-2.683208	0.000101	3.396081
H	-1.161547	0.884991	3.207514
H	-2.173376	-3.299212	0.324638
H	-2.028175	-2.545739	-1.812878
H	-2.029880	2.549353	-1.810036
H	-3.621352	1.775963	-2.040214
H	-2.189826	-1.114344	-2.840960
H	-3.125664	-2.679782	1.697295
H	-1.360169	-2.784982	1.795943
H	-1.163828	-0.888606	3.206883
H	-2.167587	3.300084	0.327646
H	-2.187045	1.118721	-2.839647
H	-3.621521	-1.775418	-2.040013
O	0.001913	-3.165219	-0.716255
O	0.003375	3.165290	-0.716941
N	1.225585	-1.303009	-0.173658
N	1.226120	1.302507	-0.174173
C	1.092615	-2.673954	-0.369603
C	2.272579	-3.479019	-0.157687
C	3.474023	-2.905141	0.128469
C	3.587233	-1.498088	0.218328
C	2.457770	-0.739887	0.053855
C	2.458027	0.738931	0.053887
C	3.587666	1.496712	0.219026
C	3.475021	2.903819	0.129216
C	2.273929	3.478149	-0.157472
C	1.093753	2.673541	-0.369962
H	2.155025	-4.551807	-0.257933
H	4.355683	-3.522478	0.272546
H	4.547992	-1.038338	0.402703

H	4.548139	1.036605	0.404008
H	4.356831	3.520820	0.273812
H	2.156780	4.550989	-0.257643

Table S4. Cartesian coordinates (Å) of **IM2**

Atom	x	y	z
Ir	-0.704633	0.269667	0.842395
C	-1.342724	0.220692	2.887342
C	-0.443518	1.346015	2.743957
C	0.807697	0.832614	2.319632
C	0.701788	-0.626787	2.251209
C	-0.609308	-0.997940	2.647714
C	-2.762012	0.299868	3.333605
C	-0.789607	2.759083	3.075699
C	2.055305	1.609842	2.075929
C	1.821979	-1.549502	1.913008
C	-1.146615	-2.372982	2.868018
H	-3.225627	1.234585	3.015764
H	-2.799064	0.256014	4.427846
H	-0.784566	2.883691	4.163874
H	-1.785504	3.017682	2.710368
H	2.671076	1.136271	1.310019
H	2.501261	-1.098085	1.188289
H	-2.155603	-2.474185	2.463712
H	-1.190266	-2.570233	3.944456
H	1.438356	-2.476126	1.489633
H	2.639713	1.656182	3.001506
H	1.815443	2.618794	1.745061
H	-0.080936	3.450366	2.626677
H	-3.346774	-0.532923	2.940708
H	-0.521542	-3.120895	2.387133
H	2.394356	-1.774749	2.819446
O	0.105617	3.368956	0.261995
O	-0.484196	-2.892949	0.004828
N	-1.147874	1.659558	-0.611159
N	-1.443573	-0.926696	-0.675404
C	-0.752936	2.991926	-0.557498
C	-1.380579	3.894566	-1.494169
C	-2.257520	3.439138	-2.430920
C	-2.564544	2.059792	-2.508878
C	-1.990702	1.211474	-1.599846
C	-2.169308	-0.256329	-1.626114
C	-2.939998	-0.915630	-2.550310
C	-2.957955	-2.326330	-2.526097
C	-2.173221	-3.007806	-1.640961
C	-1.326133	-2.305262	-0.717745
H	-1.107345	4.940790	-1.420218
H	-2.714774	4.128565	-3.134251
H	-3.228540	1.690067	-3.277633

H	-3.512808	-0.373086	-3.289224
H	-3.577978	-2.866085	-3.235285
H	-2.128741	-4.090392	-1.622827
H	1.096503	-1.069586	-0.918610
H	1.152005	-3.134889	-0.885926
C	1.765221	-1.485365	-1.674896
O	1.948320	-2.868209	-1.383798
C	3.071972	-0.732507	-1.581206
C	3.037910	0.655653	-1.416988
C	4.307060	-1.372098	-1.671917
C	4.215886	1.392836	-1.349679
H	2.077948	1.158715	-1.324557
C	5.489871	-0.635462	-1.605633
H	4.337581	-2.450598	-1.782277
C	5.448923	0.747840	-1.445922
H	4.172110	2.469117	-1.212521
H	6.446105	-1.145362	-1.675623
H	6.370009	1.319693	-1.390447
C	1.111369	-1.310397	-3.044829
H	0.149937	-1.830956	-3.071614
H	0.936710	-0.252387	-3.261457
H	1.757123	-1.725389	-3.824675

Table S5. Cartesian coordinates (Å) of **TS1**

Atom	x	y	z
Ir	-0.261595	-0.028194	0.480169
C	-1.282601	0.043463	2.442388
C	-0.385167	1.158490	2.340761
C	0.947357	0.663567	2.156684
C	0.883446	-0.790187	2.195545
C	-0.479469	-1.162310	2.367987
C	-2.750978	0.100112	2.718386
C	-0.771503	2.597882	2.428563
C	2.188334	1.493640	2.095980
C	2.057103	-1.711051	2.124170
C	-1.010971	-2.548819	2.533514
H	-3.188653	1.024990	2.338877
H	-2.942177	0.052811	3.796251
H	-0.735664	2.920272	3.474619
H	-1.785873	2.759748	2.059385
H	3.005809	0.942002	1.630218
H	2.818079	-1.323363	1.445004
H	-2.005072	-2.647853	2.093518
H	-1.091333	-2.780947	3.600738
H	1.762429	-2.696381	1.761417
H	2.500903	1.786316	3.104429
H	2.010602	2.388927	1.498795
H	-0.091391	3.213555	1.841130
H	-3.273276	-0.735852	2.248890

H	-0.361994	-3.284121	2.060877
H	2.507254	-1.825616	3.116038
O	0.587971	2.968820	-0.466391
O	-0.547114	-3.202327	-0.512649
N	-1.055027	1.408344	-0.816774
N	-1.550313	-1.144525	-0.781054
C	-0.524494	2.675065	-0.964674
C	-1.317402	3.621034	-1.707682
C	-2.506263	3.261765	-2.277452
C	-2.975455	1.938132	-2.157889
C	-2.218093	1.050076	-1.429480
C	-2.539410	-0.390668	-1.334058
C	-3.696559	-0.973128	-1.815426
C	-3.816374	-2.364462	-1.764539
C	-2.762067	-3.129727	-1.311359
C	-1.599352	-2.486507	-0.861422
H	-0.920664	4.625927	-1.798005
H	-3.086143	3.988671	-2.838359
H	-3.888657	1.628407	-2.646929
H	-4.492410	-0.367106	-2.226534
H	-4.722472	-2.842930	-2.120579
H	-2.783608	-4.212272	-1.319492
H	0.958409	-0.299584	-0.638593
H	0.363698	-2.775341	-0.818166
C	1.735778	-1.065017	-1.571522
O	1.636146	-2.312191	-1.275826
C	3.059395	-0.397079	-1.271718
C	3.183253	0.994058	-1.217454
C	4.184595	-1.195540	-1.064793
C	4.424188	1.574685	-0.980995
H	2.297854	1.620448	-1.288324
C	5.428114	-0.612028	-0.829121
H	4.072611	-2.273751	-1.091693
C	5.551717	0.774811	-0.791341
H	4.509298	2.655726	-0.925162
H	6.298820	-1.241477	-0.672979
H	6.518672	1.231411	-0.603699
C	1.028448	-0.577328	-2.827099
H	0.026520	-1.004049	-2.877728
H	0.959837	0.510338	-2.868381
H	1.606977	-0.929698	-3.688061

Table S6. Cartesian coordinates (Å) of **IM3**

Atom	x	y	z
Ir	0.458664	-0.045285	-0.379102
C	1.369554	-0.271136	1.719737
C	1.611305	1.041019	1.253894
C	2.359753	0.971390	0.012929
C	2.628114	-0.421565	-0.246590

C	1.983348	-1.187146	0.775200
C	0.664505	-0.678009	2.975256
C	1.185484	2.310889	1.915872
C	2.991958	2.135904	-0.681867
C	3.534491	-0.949706	-1.314355
C	2.114510	-2.660711	1.006080
H	0.009055	0.115724	3.337732
H	1.385563	-0.908012	3.767510
H	1.983587	2.666085	2.576662
H	0.286782	2.168192	2.518655
H	3.260911	1.880279	-1.708785
H	3.476522	-0.341922	-2.218268
H	1.183147	-3.097117	1.370311
H	2.886025	-2.836078	1.763537
H	3.287964	-1.977945	-1.583744
H	3.906237	2.439473	-0.158752
H	2.299470	2.976216	-0.716840
H	0.985704	3.081630	1.171287
H	0.053803	-1.569732	2.814442
H	2.405768	-3.194945	0.101296
H	4.571238	-0.933513	-0.960789
O	0.017663	3.136585	-1.103256
O	-0.102678	-3.120651	-1.057884
N	-1.168301	1.306010	-0.382415
N	-1.246427	-1.282249	-0.313616
C	-1.036954	2.661637	-0.619561
C	-2.159609	3.494783	-0.268910
C	-3.318897	2.964459	0.221239
C	-3.440260	1.569981	0.372831
C	-2.353539	0.788573	0.051726
C	-2.406245	-0.686332	0.059971
C	-3.537377	-1.440842	0.324146
C	-3.478684	-2.825053	0.161391
C	-2.320235	-3.419000	-0.302414
C	-1.221278	-2.597873	-0.552591
H	-2.037443	4.561592	-0.418334
H	-4.152646	3.610267	0.479876
H	-4.365157	1.129906	0.718936
H	-4.458963	-0.965743	0.630623
H	-4.352837	-3.431819	0.370403
H	-2.240940	-4.482379	-0.488351
H	0.359473	-0.132454	-1.976725
H	0.513119	-2.372139	-1.222392

Table S7. Cartesian coordinates (Å) of **TS2**

Atom	x	y	z
Ir	-0.436274	0.190352	-0.459730
C	-1.246949	0.132674	1.724280
C	-1.600678	-1.093373	1.100296

C	-2.396316	-0.818498	-0.073593
C	-2.569351	0.604544	-0.146214
C	-1.831784	1.194430	0.951114
C	-0.452564	0.299279	2.980075
C	-1.233341	-2.451814	1.594277
C	-3.112451	-1.839851	-0.898229
C	-3.481086	1.335348	-1.079744
C	-1.827148	2.638372	1.333784
H	0.221446	-0.544419	3.136825
H	-1.118108	0.365279	3.847187
H	-1.933969	-2.742174	2.384418
H	-0.227395	-2.464778	2.018064
H	-3.408019	-1.425712	-1.863495
H	-3.543434	0.832288	-2.045160
H	-0.881206	2.920991	1.797916
H	-2.629955	2.819464	2.056100
H	-3.140265	2.357304	-1.249768
H	-4.018458	-2.169395	-0.378264
H	-2.467666	-2.698389	-1.080913
H	-1.282112	-3.186238	0.792151
H	0.148152	1.210329	2.949211
H	-1.977725	3.281591	0.468675
H	-4.487263	1.377108	-0.650053
O	-0.186567	-2.937936	-1.415178
O	0.176607	3.104168	-0.988519
N	1.097042	-1.259867	-0.521268
N	1.298395	1.277641	-0.268367
C	0.887013	-2.577772	-0.883706
C	1.956559	-3.500876	-0.593656
C	3.133694	-3.080659	-0.045227
C	3.336518	-1.711676	0.227744
C	2.308470	-0.841444	-0.036834
C	2.436675	0.624361	0.063686
C	3.591727	1.324571	0.340556
C	3.558188	2.724589	0.221641
C	2.424612	3.373311	-0.209177
C	1.254261	2.624652	-0.509561
H	1.777863	-4.541470	-0.839076
H	3.925027	-3.793934	0.164530
H	4.281901	-1.353613	0.612436
H	4.505887	0.811430	0.606048
H	4.452703	3.298383	0.442805
H	2.396166	4.446691	-0.350625
H	-0.365244	0.147897	-2.042637
H	-0.551744	1.716695	-1.116924

Table S8. Cartesian coordinates (Å) of **IM-4**

Atom	x	y	z
Ir	0.472910	-0.000007	-0.376827

C	1.110641	-0.000033	1.689236
C	1.704885	1.167375	1.069187
C	2.539118	0.729880	0.005924
C	2.539128	-0.729879	0.005903
C	1.704898	-1.167418	1.069150
C	0.233877	-0.000057	2.900759
C	1.489607	2.574350	1.513047
C	3.359876	1.611416	-0.879633
C	3.359902	-1.611373	-0.879680
C	1.489641	-2.574408	1.512971
H	-0.407704	0.882460	2.920255
H	0.841211	-0.000075	3.811667
H	2.189137	2.796376	2.325997
H	0.476471	2.719670	1.890996
H	3.565269	1.130433	-1.836957
H	3.565256	-1.130373	-1.837003
H	0.476511	-2.719750	1.890930
H	2.189185	-2.796451	2.325905
H	2.838025	-2.549400	-1.073140
H	4.318218	1.835147	-0.399876
H	2.837960	2.549421	-1.073093
H	1.648776	3.275210	0.696427
H	-0.407704	-0.882576	2.920220
H	1.648804	-3.275243	0.696329
H	4.318263	-1.835069	-0.399945
O	0.025043	3.085311	-1.149180
O	0.024970	-3.085316	-1.149197
N	-1.157779	1.291800	-0.358828
N	-1.157806	-1.291781	-0.358827
C	-1.032033	2.637570	-0.647388
C	-2.169611	3.459092	-0.326509
C	-3.326622	2.909506	0.152520
C	-3.438282	1.514739	0.325053
C	-2.337197	0.739725	0.045207
C	-2.337212	-0.739683	0.045209
C	-3.438312	-1.514673	0.325058
C	-3.326681	-2.909443	0.152529
C	-2.169684	-3.459053	-0.326504
C	-1.032091	-2.637554	-0.647392
H	-2.069406	4.525193	-0.495188
H	-4.175982	3.546126	0.382131
H	-4.369652	1.068170	0.645037
H	-4.369672	-1.068084	0.645044
H	-4.176053	-3.546045	0.382146
H	-2.069500	-4.525156	-0.495182
H	0.492228	0.433846	-2.062133
H	0.492218	-0.433860	-2.062133

Table S9. Cartesian coordinates (Å) of **IM5**

Atom	x	y	z
Ir	0.673427	-0.521003	-0.168727
C	2.190103	-1.565409	1.184031
C	2.813736	-1.210898	-0.038534
C	2.108902	-1.850850	-1.129638
C	1.062246	-2.661697	-0.546564
C	1.090506	-2.456792	0.863864
C	2.604525	-1.169267	2.566577
C	4.005809	-0.324981	-0.203184
C	2.569340	-1.889966	-2.552531
C	0.195620	-3.637327	-1.279745
C	0.280151	-3.195794	1.879976
H	3.198179	-0.253328	2.555706
H	3.207397	-1.955651	3.034590
H	4.916832	-0.932589	-0.231338
H	4.096661	0.381674	0.623768
H	1.751820	-2.166835	-3.221516
H	0.683151	-4.617851	-1.315899
H	0.041885	-2.569265	2.740073
H	0.856282	-4.057086	2.236571
H	0.016427	-3.312730	-2.306123
H	3.370149	-2.628742	-2.674386
H	2.932501	-0.909097	-2.857290
H	3.934104	0.238057	-1.134152
H	1.734889	-0.995313	3.204985
H	-0.658686	-3.558533	1.463443
H	-0.773606	-3.754779	-0.793402
O	2.161857	1.263102	-2.475723
O	-2.056742	-1.309726	1.683950
N	1.097282	1.518139	-0.451904
N	-0.596317	0.446522	1.253825
C	1.813567	2.009336	-1.529513
C	2.146311	3.410082	-1.503749
C	1.736193	4.223868	-0.485756
C	0.966201	3.693913	0.564952
C	0.670120	2.348052	0.537505
C	-0.203786	1.716443	1.547303
C	-0.633123	2.361633	2.694094
C	-1.519028	1.707004	3.547379
C	-1.993796	0.460784	3.199780
C	-1.536722	-0.132704	2.017814
H	2.729016	3.786021	-2.337100
H	1.990278	5.279739	-0.489549
H	0.595707	4.334676	1.352468
H	-0.283945	3.357409	2.928421
H	-1.856752	2.188616	4.458552
H	-2.728299	-0.070811	3.791479
H	-0.424152	-0.294281	-1.317024
H	-2.134039	-1.454511	0.697771

O	-2.519890	-1.714462	-0.874911
H	-1.667743	-1.354602	-1.198201
C	-2.474870	2.669528	-1.844783
C	-2.767961	1.333112	-2.104353
C	-3.397698	0.537421	-1.145357
C	-3.745680	1.112465	0.078929
C	-3.455547	2.447919	0.343334
C	-2.815035	3.229236	-0.615939
H	-1.973078	3.269464	-2.597557
H	-2.489377	0.897664	-3.060204
H	-4.231007	0.515837	0.843908
H	-3.718703	2.874756	1.305814
H	-2.577823	4.267008	-0.404225
C	-3.606744	-0.938179	-1.430532
H	-3.589314	-1.081638	-2.516955
C	-4.887427	-1.529519	-0.874303
H	-5.754742	-0.984247	-1.254156
H	-4.970898	-2.576477	-1.174863
H	-4.899650	-1.487100	0.217737

Table S10. Cartesian coordinates (Å) of **TS3**

Atom	x	y	z
Ir	0.687683	-0.650322	-0.216615
C	2.303718	-1.441217	1.062551
C	2.839106	-1.238230	-0.255065
C	2.097871	-2.036965	-1.181090
C	1.129812	-2.808669	-0.415543
C	1.270164	-2.450399	0.955354
C	2.826702	-0.855693	2.336065
C	3.985512	-0.343881	-0.590839
C	2.381935	-2.166283	-2.643761
C	0.229010	-3.869136	-0.963003
C	0.552432	-3.062466	2.112326
H	3.285554	0.119066	2.161443
H	3.582586	-1.510788	2.782386
H	4.922084	-0.892227	-0.443913
H	4.004273	0.535024	0.056191
H	1.511969	-2.549929	-3.179596
H	0.754393	-4.829783	-0.985662
H	0.399064	-2.336263	2.911996
H	1.155642	-3.884996	2.511638
H	-0.089698	-3.631479	-1.978734
H	3.215236	-2.856954	-2.811864
H	2.635479	-1.191043	-3.061378
H	3.924257	-0.005006	-1.623451
H	2.024790	-0.721578	3.064758
H	-0.424201	-3.442368	1.819470
H	-0.665757	-3.979039	-0.349661
O	2.009588	1.190189	-2.514517

O	-2.041106	-1.559669	1.449657
N	1.043485	1.390931	-0.442688
N	-0.605835	0.221323	1.205296
C	1.714996	1.912940	-1.532471
C	2.057085	3.308701	-1.459837
C	1.683956	4.076884	-0.391540
C	0.936338	3.514060	0.659765
C	0.626860	2.174217	0.588458
C	-0.254835	1.490342	1.559245
C	-0.750011	2.091994	2.695498
C	-1.665616	1.378092	3.482993
C	-2.090098	0.138146	3.082388
C	-1.588526	-0.447254	1.883833
H	2.613761	3.720838	-2.293834
H	1.946826	5.130237	-0.359063
H	0.587298	4.126552	1.479272
H	-0.450487	3.094128	2.969201
H	-2.057639	1.822713	4.392485
H	-2.828994	-0.426738	3.638334
H	-0.316075	-0.390993	-1.527142
H	-2.241657	-1.629049	-0.005500
O	-2.242283	-1.610067	-1.041247
H	-1.011898	-0.991465	-1.141180
C	-2.184066	2.788243	-1.827866
C	-2.451536	1.457004	-2.135278
C	-3.148278	0.640165	-1.242350
C	-3.588814	1.188848	-0.036404
C	-3.327686	2.520384	0.273809
C	-2.621808	3.323097	-0.619248
H	-1.627526	3.403677	-2.527775
H	-2.099766	1.041972	-3.076068
H	-4.122592	0.574218	0.680239
H	-3.663255	2.926468	1.222531
H	-2.405990	4.357169	-0.370118
C	-3.334092	-0.829082	-1.577028
H	-3.249030	-0.942764	-2.661365
C	-4.648828	-1.435931	-1.116654
H	-5.492250	-0.875951	-1.529142
H	-4.714595	-2.472204	-1.456064
H	-4.728221	-1.428976	-0.026526

Table S11. Cartesian coordinates (Å) of **IM6**

Atom	x	y	z
Ir	0.625812	-0.393672	-0.038833
C	2.269106	-1.286070	1.062303
C	2.772853	-0.961040	-0.253496
C	2.002416	-1.671258	-1.215134
C	1.063917	-2.525443	-0.492993
C	1.265025	-2.318856	0.897320

C	2.845579	-0.826571	2.363391
C	3.924795	-0.054051	-0.523786
C	2.203148	-1.628130	-2.696155
C	0.128827	-3.518054	-1.105539
C	0.622485	-3.064331	2.017315
H	3.249244	0.183715	2.278745
H	3.656116	-1.491941	2.678428
H	4.853454	-0.616853	-0.380672
H	3.933387	0.790796	0.166944
H	1.294146	-1.916962	-3.225906
H	0.615252	-4.496419	-1.174821
H	0.424056	-2.410252	2.867579
H	1.308123	-3.851958	2.348389
H	-0.166801	-3.214301	-2.110559
H	3.001050	-2.318126	-2.989158
H	2.474172	-0.618465	-3.007637
H	3.889900	0.337980	-1.537550
H	2.086674	-0.821676	3.147572
H	-0.320467	-3.510155	1.710482
H	-0.775166	-3.618537	-0.503734
O	1.954356	1.766084	-1.983190
O	-1.959292	-1.689382	1.361146
N	0.959036	1.653242	0.076113
N	-0.685071	0.210257	1.465681
C	1.647026	2.334720	-0.910827
C	1.979348	3.703793	-0.616145
C	1.559197	4.301745	0.539402
C	0.769701	3.589973	1.465150
C	0.478045	2.274653	1.188268
C	-0.461133	1.451798	1.979678
C	-1.146936	1.911607	3.081591
C	-2.119562	1.074032	3.656076
C	-2.394162	-0.147121	3.100270
C	-1.688795	-0.601355	1.939828
H	2.560783	4.238290	-1.358671
H	1.813331	5.338541	0.738985
H	0.382792	4.074559	2.350811
H	-0.962629	2.899519	3.480302
H	-2.669415	1.410671	4.529665
H	-3.159668	-0.801966	3.499607
H	-0.273658	0.126995	-1.428242
H	-2.720274	-1.869338	-0.159165
O	-3.230398	-2.005344	-0.992849
H	-0.830344	-0.368341	-0.984270
C	-1.955607	1.529205	-3.159082
C	-2.778998	0.463166	-2.811982
C	-3.307876	0.349752	-1.521688
C	-3.009769	1.346246	-0.590962
C	-2.189052	2.419334	-0.934908
C	-1.653827	2.511218	-2.215318

H	-1.544441	1.592932	-4.161821
H	-3.002582	-0.306902	-3.545598
H	-3.400973	1.286591	0.418838
H	-1.951923	3.174386	-0.192288
H	-0.999251	3.336818	-2.474998
C	-4.110815	-0.895700	-1.175494
H	-4.709176	-1.154209	-2.055723
C	-5.046515	-0.738173	0.016614
H	-5.731394	0.102371	-0.128326
H	-5.634538	-1.651102	0.137874
H	-4.485076	-0.575691	0.940752

Table S12. Cartesian coordinates (Å) of **IM7**

Atom	x	y	z
Ir	-0.402072	-0.201715	-0.269522
C	-1.172159	-0.171369	1.874078
C	-0.945701	-1.511658	1.484280
C	-1.763934	-1.804999	0.321589
C	-2.553748	-0.629181	0.046825
C	-2.162366	0.387269	0.965324
C	-0.562785	0.557806	3.029850
C	-0.017455	-2.488095	2.130630
C	-1.989581	-3.164810	-0.258696
C	-3.679474	-0.547918	-0.935230
C	-2.828722	1.713878	1.146251
H	0.362458	0.080817	3.357597
H	-1.251808	0.579887	3.881278
H	-0.565993	-3.096301	2.857806
H	0.795376	-1.983471	2.655824
H	-2.380378	-3.096872	-1.276005
H	-3.489868	-1.174260	-1.808474
H	-2.131127	2.472149	1.501627
H	-3.630340	1.613592	1.886723
H	-3.837371	0.473321	-1.281417
H	-2.717965	-3.716226	0.347533
H	-1.054908	-3.721411	-0.300587
H	0.413679	-3.154813	1.382681
H	-0.330691	1.592529	2.765641
H	-3.267857	2.074846	0.216091
H	-4.603047	-0.899921	-0.462393
O	0.980185	-3.043891	-1.118793
O	-0.894352	3.135988	-0.681045
N	1.565847	-0.924965	-0.448521
N	0.785861	1.582756	-0.257822
C	1.871167	-2.237823	-0.761606
C	3.251568	-2.631004	-0.643578
C	4.220086	-1.737272	-0.284935
C	3.874496	-0.397759	-0.029061
C	2.546817	-0.039780	-0.124471

C	2.093643	1.355496	0.054088
C	2.928013	2.376543	0.473041
C	2.427189	3.675651	0.544511
C	1.131454	3.921375	0.148134
C	0.337713	2.848589	-0.277850
H	3.481290	-3.667910	-0.861457
H	5.256830	-2.051043	-0.207331
H	4.637070	0.327670	0.215971
H	3.953497	2.172290	0.746094
H	3.060860	4.487728	0.883778
H	0.701670	4.914948	0.137172
H	-0.414481	-0.186185	-1.882108
H	-1.291447	2.488402	-1.323051
O	-2.065127	1.734777	-2.602910
H	-1.783339	2.056158	-3.469720
H	-1.617520	0.877002	-2.462739

Table S13. Cartesian coordinates (Å) of **TS4**

Atom	x	y	z
Ir	-0.441086	-0.143941	-0.258558
C	-1.039728	-0.250373	1.861917
C	-1.230618	-1.549849	1.282995
C	-2.158534	-1.439377	0.199296
C	-2.613813	-0.058056	0.154428
C	-1.936995	0.660945	1.179621
C	-0.206910	0.076539	3.060861
C	-0.571502	-2.804947	1.748552
C	-2.685875	-2.576805	-0.615973
C	-3.688364	0.476059	-0.737300
C	-2.171314	2.081071	1.575152
H	0.642373	-0.603421	3.147523
H	-0.800889	-0.004673	3.977496
H	-1.153771	-3.227026	2.574507
H	0.438609	-2.610408	2.113522
H	-3.109348	-2.221583	-1.557005
H	-3.681847	-0.022793	-1.707163
H	-1.255419	2.548086	1.939848
H	-2.910692	2.106607	2.383031
H	-3.562487	1.545039	-0.910331
H	-3.473128	-3.105919	-0.068289
H	-1.879560	-3.273703	-0.847601
H	-0.508884	-3.534396	0.943080
H	0.183864	1.094294	3.002298
H	-2.534668	2.674746	0.738893
H	-4.667902	0.312307	-0.275809
O	0.576019	-3.069989	-1.180640
O	-0.578463	3.135424	-0.808897
N	1.418602	-1.079163	-0.411010
N	0.915868	1.479281	-0.239386

C	1.556344	-2.409488	-0.762988
C	2.870192	-2.976434	-0.608271
C	3.927427	-2.216074	-0.192352
C	3.751251	-0.846025	0.082039
C	2.486478	-0.317175	-0.047648
C	2.187162	1.124013	0.108322
C	3.118535	2.056804	0.509781
C	2.742419	3.408721	0.533646
C	1.493219	3.778358	0.107970
C	0.560493	2.797043	-0.336027
H	2.980034	-4.028272	-0.846458
H	4.912326	-2.661574	-0.087574
H	4.591826	-0.227140	0.362961
H	4.116873	1.757785	0.797468
H	3.451281	4.160350	0.866578
H	1.177266	4.814199	0.074909
H	-0.430605	-0.312591	-1.927898
H	-1.043910	2.372074	-1.924915
O	-1.318289	1.702963	-2.686025
H	-0.789783	1.900903	-3.472151
H	-0.842579	0.592832	-2.054814

Table S14. Cartesian coordinates (Å) of **IM8**

Atom	x	y	z
Ir	-0.397588	-0.220596	-0.240163
C	-0.838524	-0.566852	1.852378
C	-0.932230	-1.846358	1.185106
C	-1.937780	-1.755649	0.182763
C	-2.549800	-0.434448	0.282681
C	-1.903449	0.270201	1.332153
C	0.033078	-0.247182	3.024867
C	-0.116278	-3.048513	1.519068
C	-2.369064	-2.864244	-0.722809
C	-3.725003	0.045797	-0.505872
C	-2.275374	1.610184	1.870103
H	0.979017	-0.788454	2.969835
H	-0.465420	-0.526502	3.958755
H	-0.597387	-3.579217	2.347441
H	0.889456	-2.769233	1.837132
H	-2.844821	-2.475121	-1.624104
H	-3.744563	-0.401923	-1.499831
H	-1.396744	2.159771	2.210442
H	-2.940026	1.468238	2.729133
H	-3.696716	1.128877	-0.626559
H	-3.089524	-3.509543	-0.209795
H	-1.507513	-3.462617	-1.022035
H	-0.030263	-3.715455	0.663814
H	0.258164	0.819785	3.066516
H	-2.778944	2.215170	1.119570

H	-4.651976	-0.225225	0.010025
O	1.070940	-2.843317	-1.344504
O	-1.171495	2.920051	-0.638924
N	1.588145	-0.801738	-0.447838
N	0.642242	1.596928	-0.168265
C	1.937181	-2.068826	-0.875790
C	3.325367	-2.421180	-0.735664
C	4.242905	-1.514465	-0.281974
C	3.848997	-0.200873	0.044590
C	2.515655	0.119069	-0.063566
C	1.971142	1.481518	0.122218
C	2.732198	2.571648	0.476421
C	2.110850	3.833354	0.510152
C	0.796786	3.963713	0.150102
C	0.025748	2.823077	-0.245066
H	3.607467	-3.428703	-1.019250
H	5.287436	-1.797294	-0.190859
H	4.579114	0.537092	0.346785
H	3.782615	2.465501	0.709857
H	2.685074	4.707093	0.802713
H	0.296891	4.925095	0.136759
H	-0.373481	-0.518412	-1.952098
H	-1.697812	2.215911	-2.056144
O	-1.972677	1.787371	-2.907943
H	-1.563541	2.308221	-3.608559
H	-0.753545	0.253856	-1.894456

Table S15. Cartesian coordinates (Å) of 1-phenylethyl alcohol-water complex

Atom	x	y	z
C	-2.178029	0.055927	-1.065980
C	-0.797315	-0.014297	-1.255100
C	0.049558	-0.357147	-0.201322
C	-0.507579	-0.621834	1.053146
C	-1.882801	-0.550911	1.246666
C	-2.723635	-0.212485	0.185661
H	-2.823327	0.326331	-1.895859
H	-0.374789	0.203367	-2.232410
H	0.145955	-0.872632	1.882801
H	-2.301834	-0.757424	2.226593
H	-3.796744	-0.154769	0.337363
C	1.551478	-0.431913	-0.405928
H	1.776575	-0.050143	-1.411727
O	2.245279	0.353525	0.563172
H	1.884206	1.258937	0.515489
C	2.080594	-1.852885	-0.294069
H	1.606327	-2.498719	-1.037462
H	3.161385	-1.863758	-0.457806
H	1.873555	-2.257632	0.700759
O	0.831841	2.736173	0.081061

H	0.739349	3.468763	0.703507
H	-0.061247	2.393364	-0.063559

Table S16. Cartesian coordinates (Å) of acetophenone-water complex

Atom	x	y	z
C	-2.720673	0.979197	-0.000596
C	-1.342964	1.170696	-0.000111
C	-0.479359	0.069945	0.000357
C	-1.015052	-1.223522	0.000300
C	-2.389955	-1.413341	-0.000208
C	-3.244792	-0.311007	-0.000654
H	-3.384157	1.837343	-0.000936
H	-0.950656	2.180869	-0.000084
H	-0.339011	-2.070893	0.000649
H	-2.798263	-2.418441	-0.000264
H	-4.319900	-0.458826	-0.001049
C	0.999850	0.234846	0.000885
O	1.728354	-0.756141	0.001616
C	1.593057	1.616735	0.000404
H	1.263070	2.172998	-0.881457
H	1.262932	2.173603	0.881836
H	2.680317	1.550204	0.000545
O	4.490954	-0.462992	-0.001603
H	3.524594	-0.621666	-0.000301
H	4.905929	-1.333424	-0.001319

Table S17. Cartesian coordinates (Å) of water dimer

Atom	x	y	z
O	1.469514	-0.005479	0.118237
H	0.495519	-0.002258	0.031897
H	1.814477	0.037063	-0.781253
O	-1.315836	0.003245	-0.092581
H	-1.768099	0.761913	0.298806
H	-1.771325	-0.778844	0.245300

Table S18. Cartesian coordinates (Å) of water

Atom	x	y	z
O	0.000000	0.000000	0.117079
H	0.000000	0.767638	-0.468314
H	0.000000	-0.767638	-0.468314

Table S19. Cartesian coordinates (Å) of 1-phenylethyl alcohol

Atom	x	y	z
C	-2.094655	-1.074773	-0.159135
C	-0.716907	-1.247179	-0.280591

C	0.150754	-0.166227	-0.126071
C	-0.381665	1.096635	0.144164
C	-1.757049	1.273711	0.260172
C	-2.618110	0.186900	0.111367
H	-2.758165	-1.924951	-0.282777
H	-0.313263	-2.232162	-0.499598
H	0.289511	1.942337	0.256166
H	-2.158845	2.260594	0.467946
H	-3.690914	0.324738	0.201572
C	1.650796	-0.366003	-0.211945
H	1.846272	-1.321919	-0.715547
O	2.286593	0.689410	-0.939816
H	1.898873	0.737748	-1.823257
C	2.301389	-0.389653	1.162375
H	1.882798	-1.197192	1.767870
H	3.379310	-0.544027	1.066828
H	2.124366	0.559086	1.677309

Table S20. Cartesian coordinates (Å) of acetophenone

Atom	x	y	z
C	1.812184	1.275747	-0.000027
C	0.423350	1.188647	-0.000025
C	-0.204620	-0.061178	0.000008
C	0.578799	-1.220761	0.000029
C	1.964439	-1.133735	0.000033
C	2.583413	0.116330	-0.000009
H	2.291270	2.249103	-0.000052
H	-0.162055	2.100776	-0.000021
H	0.084106	-2.185470	0.000060
H	2.564278	-2.037869	0.000066
H	3.666490	0.185559	-0.000011
C	-1.692744	-0.198174	-0.000009
O	-2.213562	-1.306765	-0.000069
C	-2.543972	1.044828	0.000048
H	-2.332793	1.656856	0.881651
H	-2.332941	1.656824	-0.881606
H	-3.594957	0.758119	0.000166

11. Metabolic Studies

Kinetic analysis for losartan oxidation was performed under non-competitive intermolecular conditions with low (0.5 μM) and high (10 μM) concentrations of d_0-, or d_2-losartan potassium as substrates. Insect cell microsomes from a baculovirus-based system containing human P450 2C9 co-expressed with NADPH-P450 reductase were purchased from BD Gentest (Woburn, MA, USA), and purified cytochrome b5 were from and Oxford Biomedical Research (Oxford, UK). Insect cell microsomes (10 pmol P450) and purified cytochrome b5 (10 pmol) were incubated with deuterated or protiated losartan in the presence of an NADPH regenerating system at 37 °C for 10 min in 100 mM Tris-HCl buffer (pH 7.5) in a final volume of 500 μL. Reactions were terminated by the addition of 100 μL of 94% acetonitrile/6% acetic acid, followed by centrifugation at 3,000 g for 10 min at 4 °C. The supernatants were filtered through polytetra-fluoroethylene membrane filters of 0.2 μm pore size (Millipore, Bedford, MA), and the aliquots were analyzed by LC-MS as described below.

Thermo Scientific Q Exactive bench-top LC-MS instrument was used to analyze metabolic formation. Chromatographic separations were performed using a Shim-pack CLC-ODS (M) (250 x 4.6 mm i.d., Shimadzu). Mobile phase A was composed of 0.1% acetic acid and mobile phase B was composed of CH_3CN:CH_3OH at a 4:1 ratio (v/v). Elution was completed with following conditions: 0–8 min, 25–70% B; 8–8.10 min, 70–85% B; 8.1–11 min, 85% B; 11–11.10 min, 85–25% B; 11.10–15 min, 25% B gradient program operating with a 0.3 mL/min flow rate with a 5 μL injection volume. The retention times of losartan, E-3174 and E-3179 were 5.7, 6.2 and 7.6 min, respectively. Detection was performed by the single ion-monitoring (SIM) mode via negative heated electrospray ionization (HESI) for losartan ($[C_{22}H_{23}ClN_6O-H]^-$, *m*/*z* 421.1549), losartan-*d*$_2$ ($[C_{22}H_{21}D_2ClN_6O-H]^-$, *m*/*z* 423.1675), E3179 ($[C_{22}H_{21}ClN_6O-H]^-$, *m*/z 419.1393), E3179-*d*$_1$ ($[C_{22}H_{20}D_1ClN_6O-H]^-$, *m*/*z* 420.1455) and E3174 ($[C_{22}H_{21}ClN_6O_2-H]^-$, *m*/*z* 435.1342). The spray voltage was set to 2.5 kV, the sheath gas was set to 50 arb unit; the aux gas was set to 10 arb unit; and the capillary temperature was set to 250 °C.

12. Reference

(1) Thakur, N.; Aslani, S.; Armstrong, D. W. Evaluation of gas chromatography for the separation of a broad range of isotopic compounds. *Anal. Chim. Acta* **2021**, *1165*, 338490.
