# Supplementary material for: Iridium-catalyzed α-selective deuteration of alcohols
Source: Chem Sci. 2022 Jul 6;13(30):8744–51. doi: 10.1039/d2sc01805e (PMC9350590; doi:10.1039/d2sc01805e)
Supplement: SC-013-D2SC01805E-s002 [file SC-013-D2SC01805E-s002.pdf]

Electronic Supplementary Material (ESI) for Chemical Science.
This journal is © The Royal Society of Chemistry 2022

Electronic Supplementary Information 2 (NMR Charts)

Iridium-Catalyzed α-Selective Deuteration of Alcohols

Moeko Itoga,[a] Masako Yamanishi,[a] Taro Udagawa,[b] Ayane Kobayashi,[c] Keiko Maekawa,[c] Yoshiji Takemoto,[a] and Hiroshi Naka*[a]

a. Graduate School of Pharmaceutical Sciences, Kyoto University, Kyoto 606-8501, Japan. Email: h_naka@pharm.kyoto-u.ac.jp

b. Department of Chemistry and Biomolecular Science, Faculty of Engineering, Gifu University, Yanagido 1-1, Gifu 501-1193, Japan.

c. Faculty of Pharmaceutical Sciences, Doshisha Women's College of Liberal Arts, Kodo, Kyotanabe, Kyoto 610-0395, Japan.

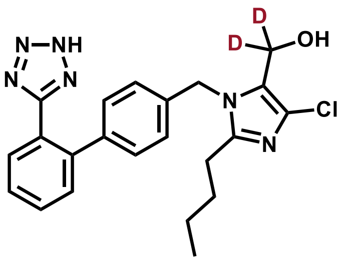
N—NH
N
N
D
D
OH
N
Cl
N

[^{2}H]1

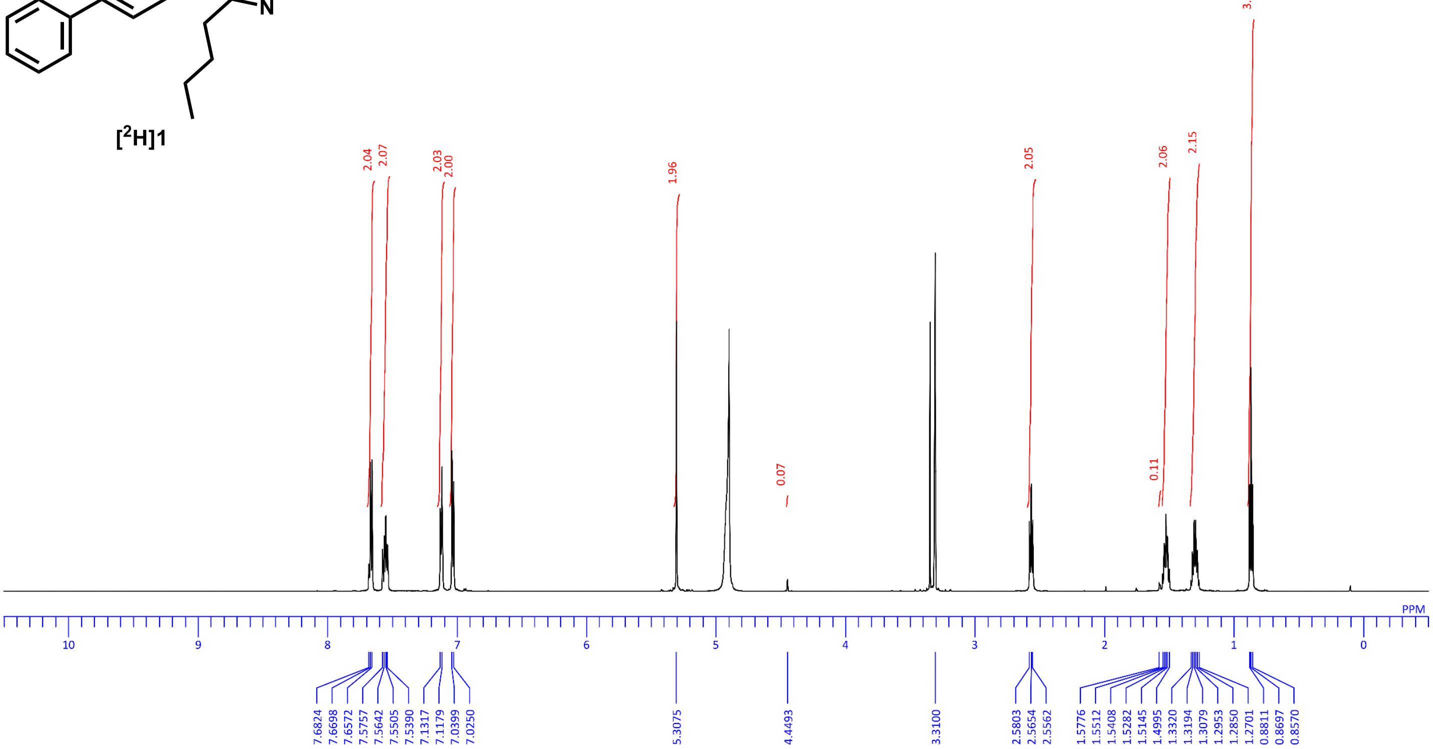
2.04
2.07
2.03
2.00
1.96
0.07
2.05
0.11
2.06
2.15
3.05
PPM
10
9
8
7
6
5
4
3
2
1
0
7.6824
7.6698
7.6572
7.5757
7.5642
7.5505
7.5390
7.1317
7.1179
7.0399
7.0250
5.3075
4.4493
3.3100
2.5803
2.5654
2.5562
1.5776
1.5512
1.5408
1.5282
1.5145
1.4995
1.3320
1.3194
1.3079
1.2953
1.2850
1.2701
0.8811
0.8697
0.8570

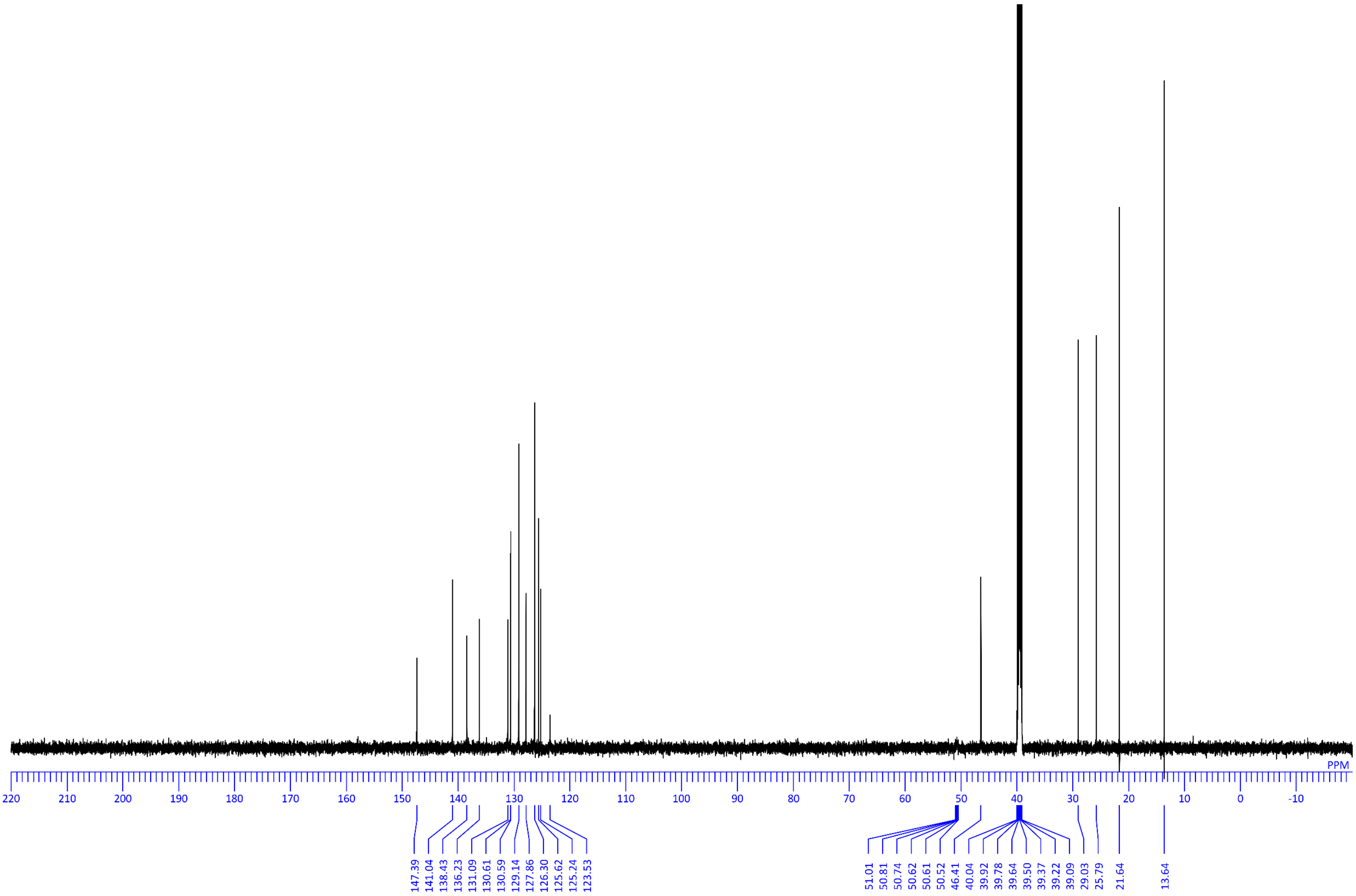
PPM
220
210
200
190
180
170
160
150
140
130
120
110
100
90
80
70
60
50
40
30
20
10
0
-10
147.39
141.04
138.43
136.23
131.09
130.61
130.59
129.14
127.86
126.30
125.62
125.24
123.53
51.01
50.81
50.74
50.62
50.61
50.52
46.41
40.04
39.92
39.78
39.64
39.50
39.37
39.22
39.09
29.03
25.79
21.64
13.64

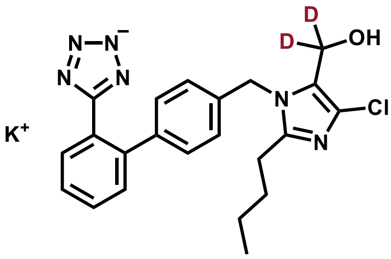
D
D
OH
N
N
N
N
N
N
Cl
K+

[^{2}H]**1**-K$^+$

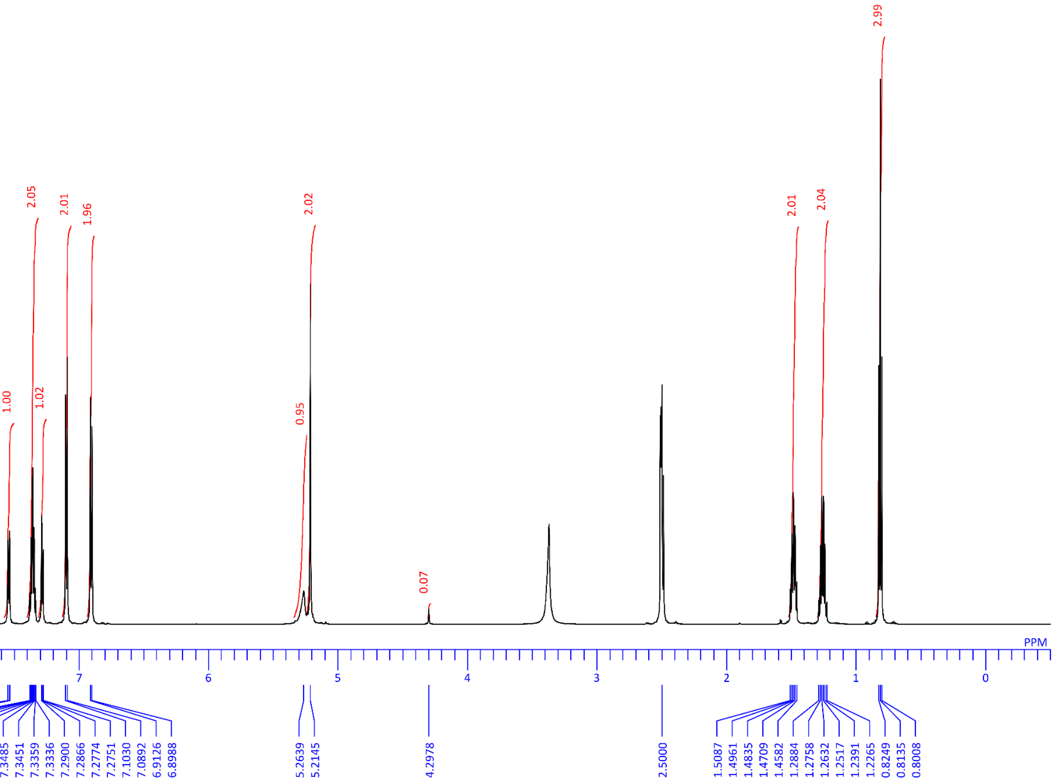
1.00
2.05
1.02
2.01
1.96
0.95
2.02
0.07
2.01
2.04
2.99
PPM
10
9
8
7
6
5
4
3
2
1
0
7.5493
7.5378
7.5344
7.3795
7.3703
7.3669
7.3589
7.3566
7.3485
7.3451
7.3359
7.3336
7.2900
7.2866
7.2774
7.2751
7.1030
7.0892
6.9126
6.8988
5.2639
5.2145
4.2978
2.5000
1.5087
1.4961
1.4835
1.4709
1.4582
1.2884
1.2758
1.2632
1.2517
1.2391
1.2265
0.8249
0.8135
0.8008

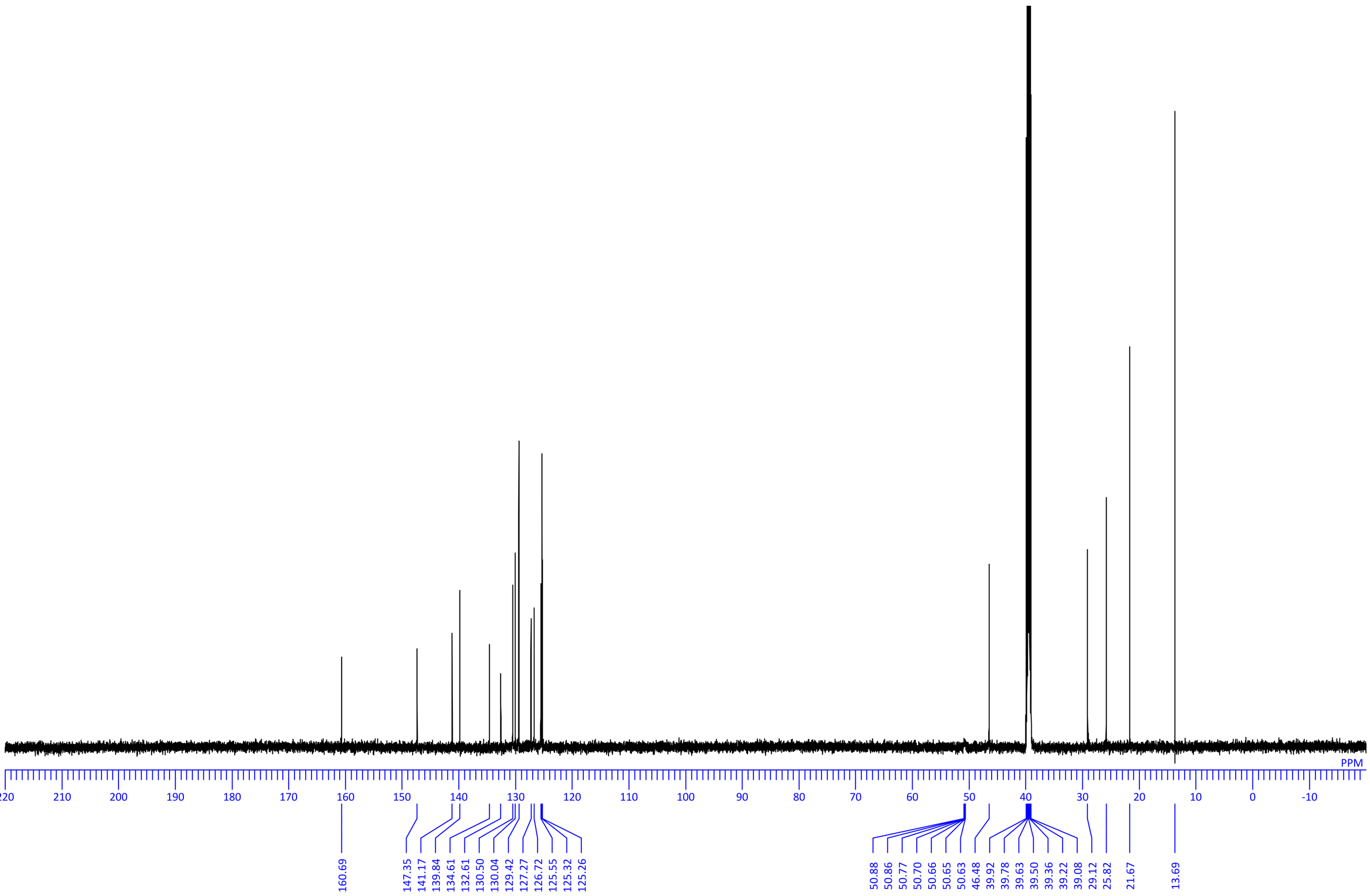
PPM
220
210
200
190
180
170
160
150
140
130
120
110
100
90
80
70
60
50
40
30
20
10
0
-10
160.69
147.35
141.17
139.84
134.61
132.61
130.50
130.04
129.42
127.27
126.72
125.55
125.32
125.26
50.88
50.86
50.77
50.70
50.66
50.65
50.63
46.48
39.92
39.78
39.63
39.50
39.36
39.22
39.08
29.12
25.82
21.67
13.69

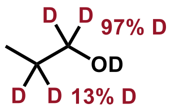
D D 97% D
OD
D D 13% D

[2H]2

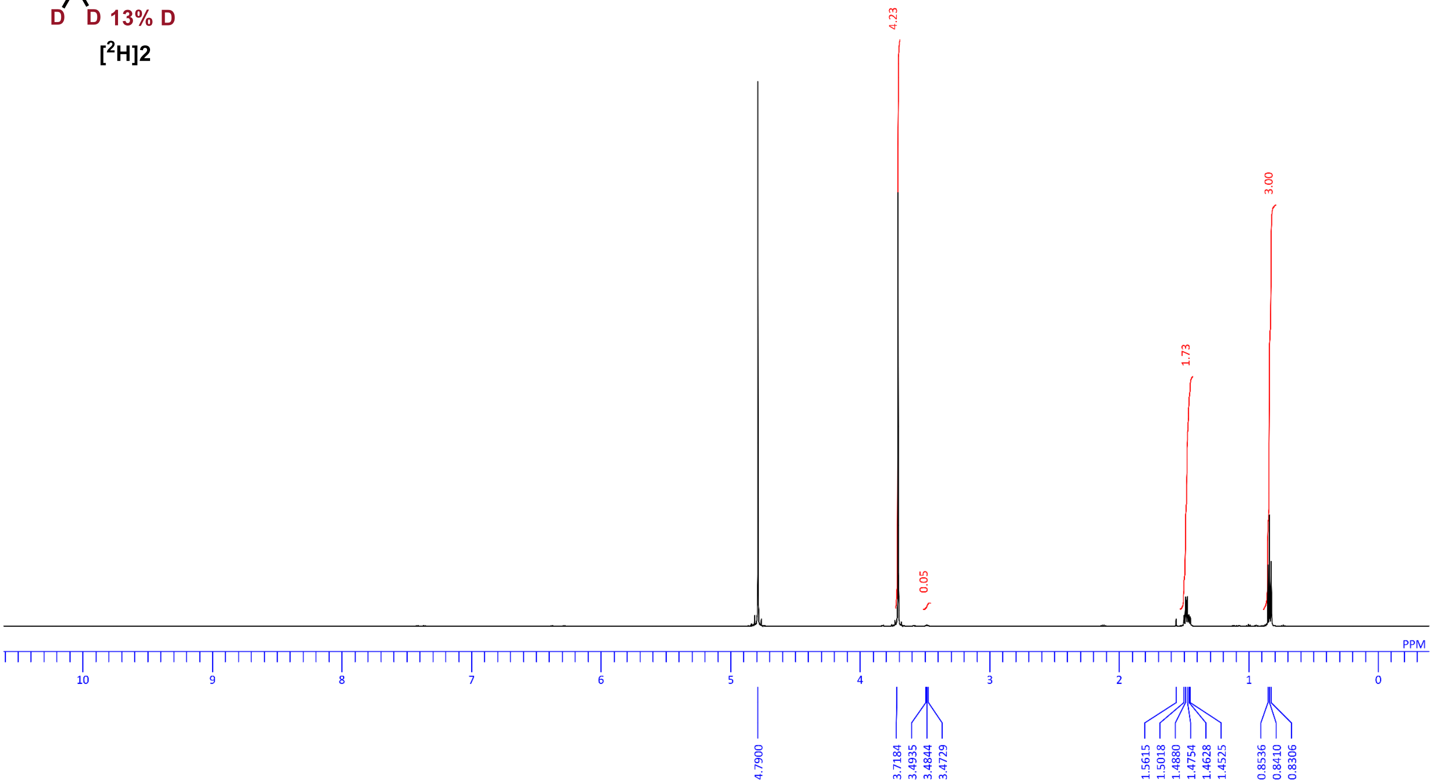
4.23
0.05
1.73
3.00
PPM
10
9
8
7
6
5
4
3
2
1
0
4.7900
3.7184
3.4935
3.4844
3.4729
1.5615
1.5018
1.4880
1.4754
1.4628
1.4525
0.8536
0.8410
0.8306

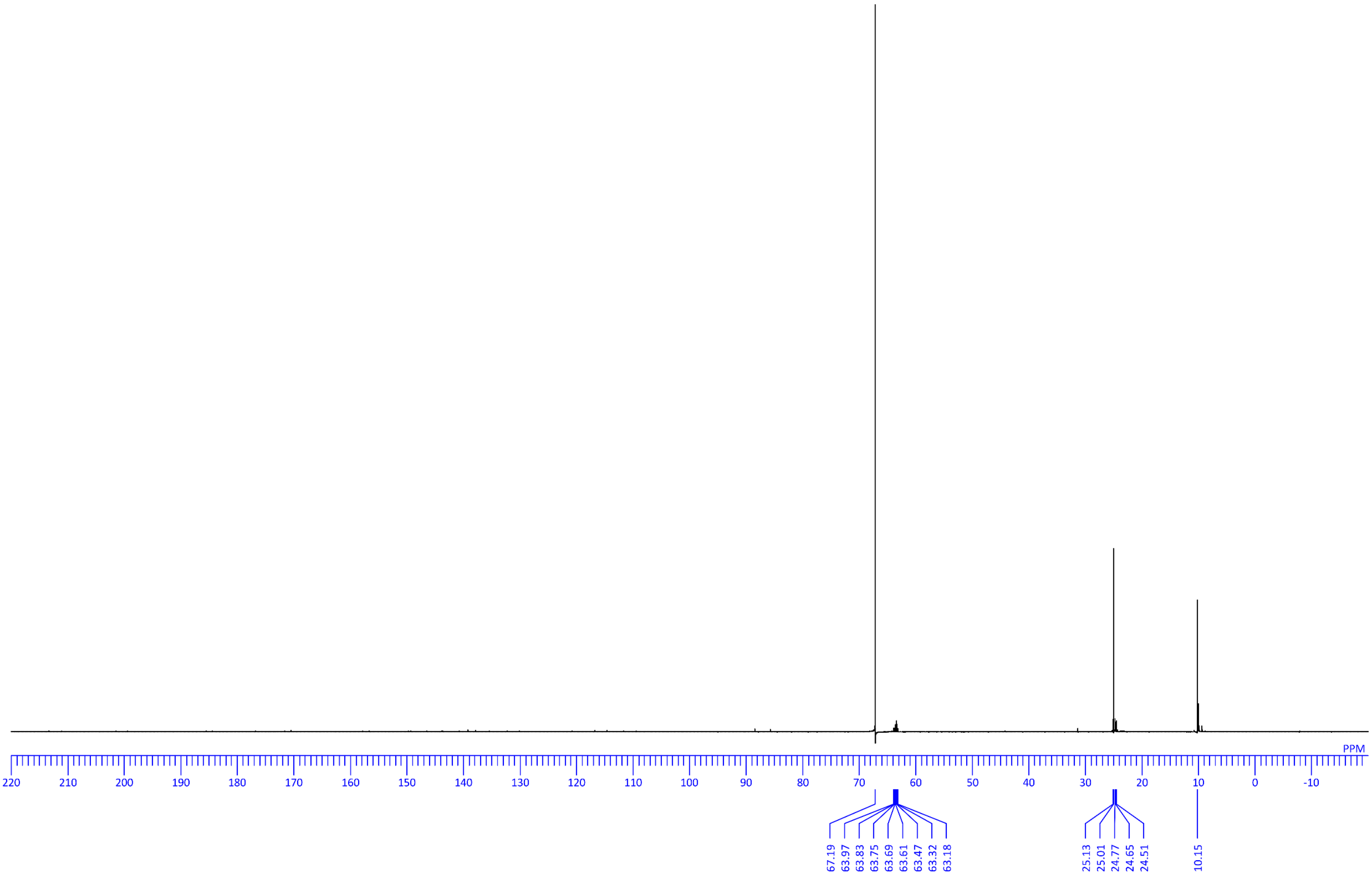
PPM
220
210
200
190
180
170
160
150
140
130
120
110
100
90
80
70
60
50
40
30
20
10
0
-10
67.19
63.97
63.83
63.75
63.69
63.61
63.47
63.32
63.18
25.13
25.01
24.77
24.65
24.51
10.15

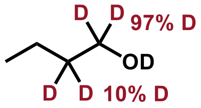
D D 97% D
OD
D D 10% D

[2H]3

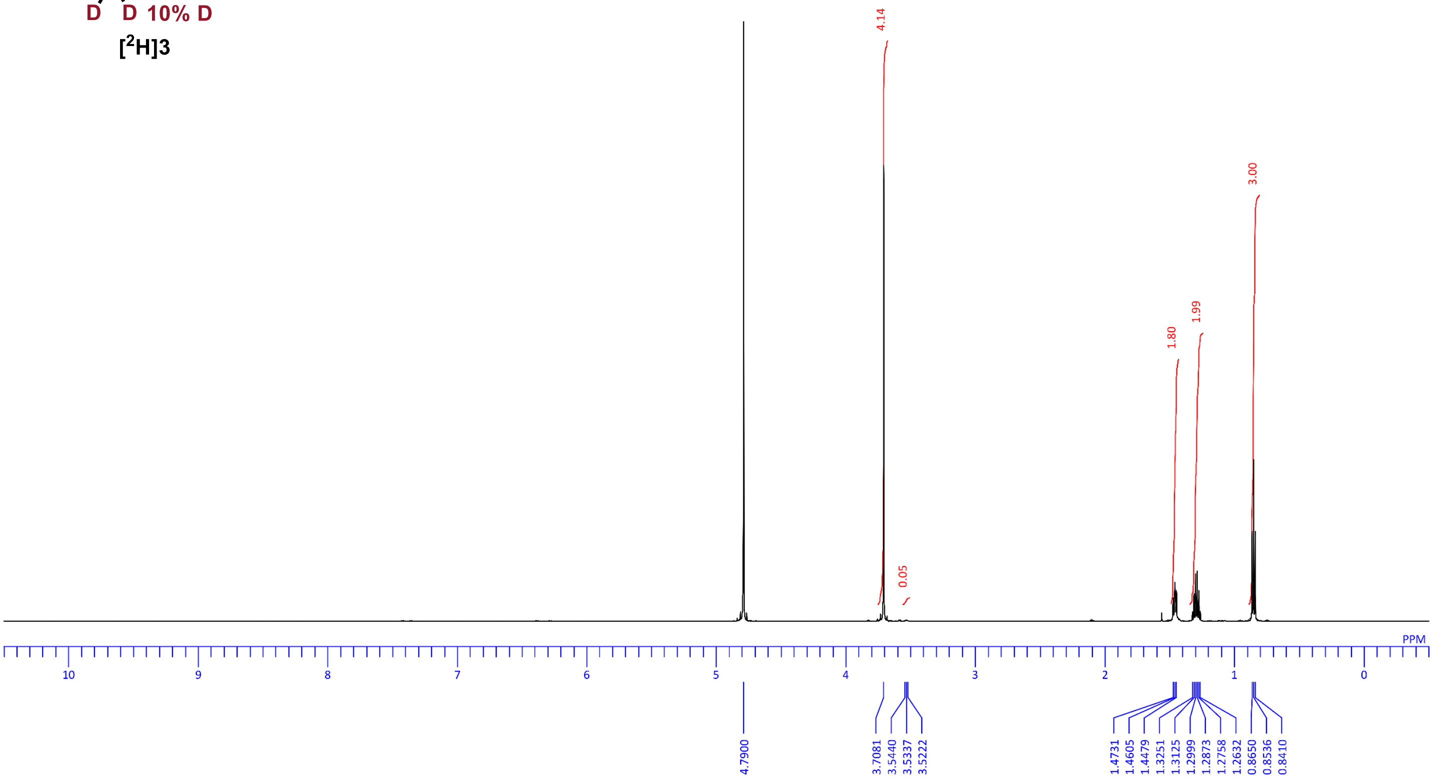
4.14
0.05
1.80
1.99
3.00
PPM
10 9 8 7 6 5 4 3 2 1 0
4.7900
3.7081
3.5440
3.5337
3.5222
1.4731
1.4605
1.4479
1.3251
1.3125
1.2999
1.2873
1.2758
1.2632
0.8650
0.8536
0.8410

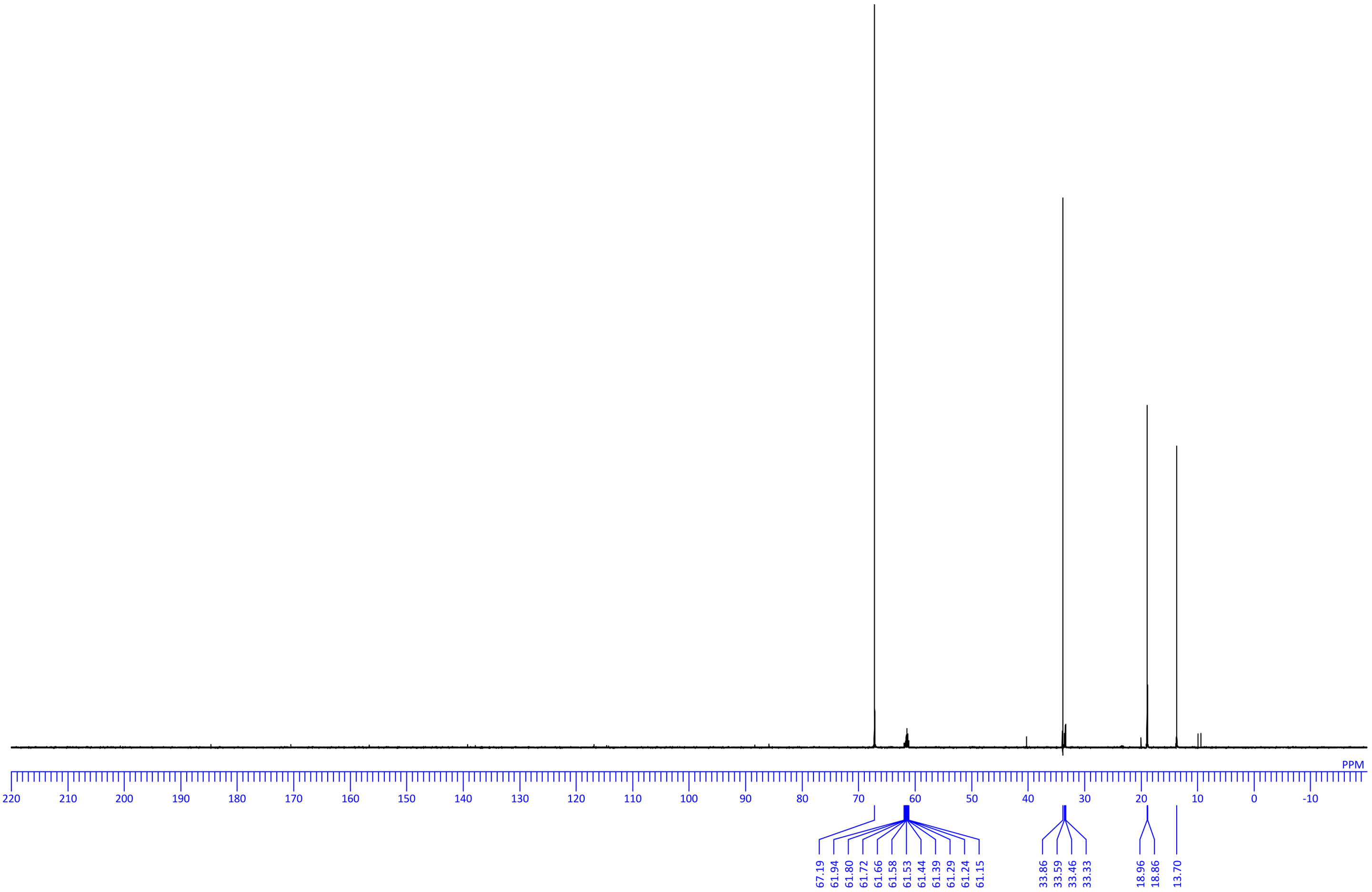
PPM
220 210 200 190 180 170 160 150 140 130 120 110 100 90 80 70 60 50 40 30 20 10 0 -10
67.19
61.94
61.80
61.72
61.66
61.58
61.53
61.44
61.39
61.29
61.24
61.15
33.86
33.59
33.46
33.33
18.96
18.86
13.70

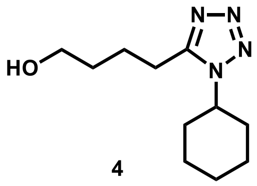
HO
N
N
N
N

4

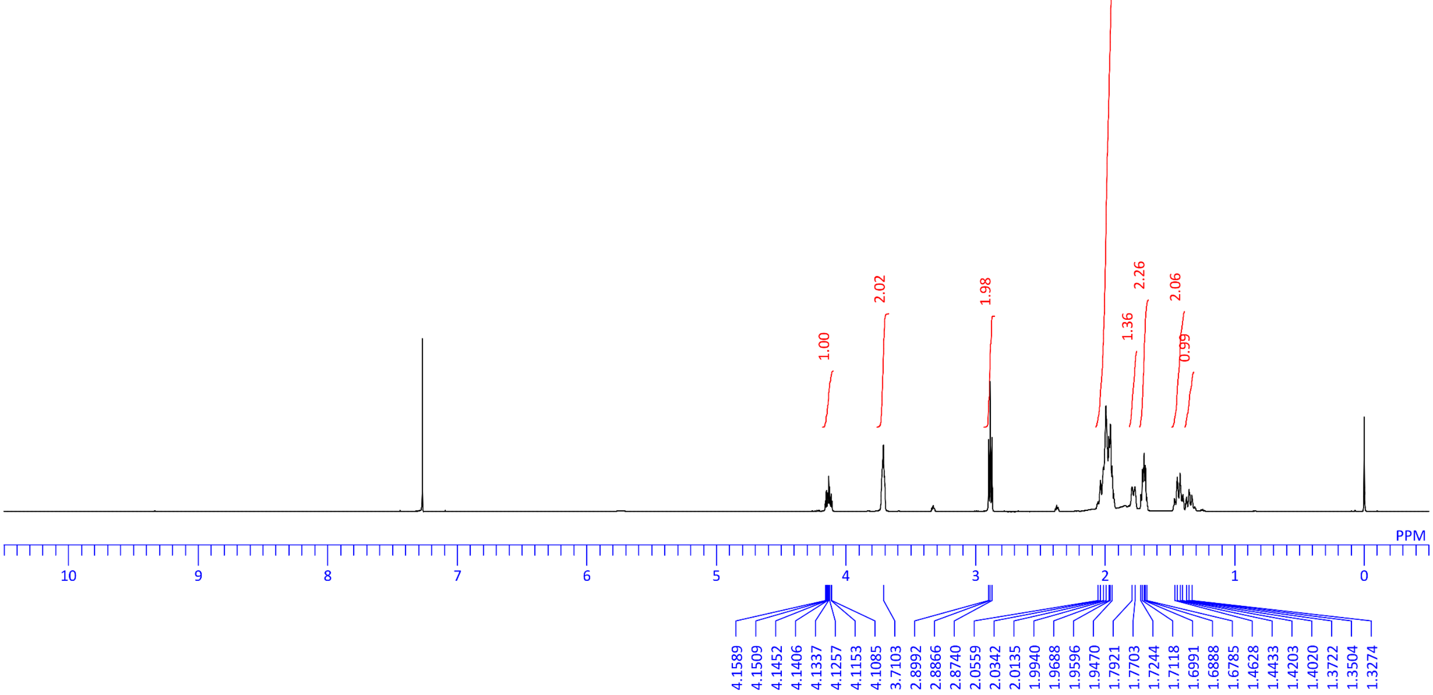
8.48
2.26
2.06
1.36
0.99
2.02
1.98
1.00
PPM
10
9
8
7
6
5
4
3
2
1
0
4.1589
4.1509
4.1452
4.1406
4.1337
4.1257
4.1153
4.1085
3.7103
2.8992
2.8866
2.8740
2.0559
2.0342
2.0135
1.9940
1.9688
1.9596
1.9470
1.7921
1.7703
1.7244
1.7118
1.6991
1.6888
1.6785
1.4628
1.4433
1.4203
1.4020
1.3722
1.3504
1.3274

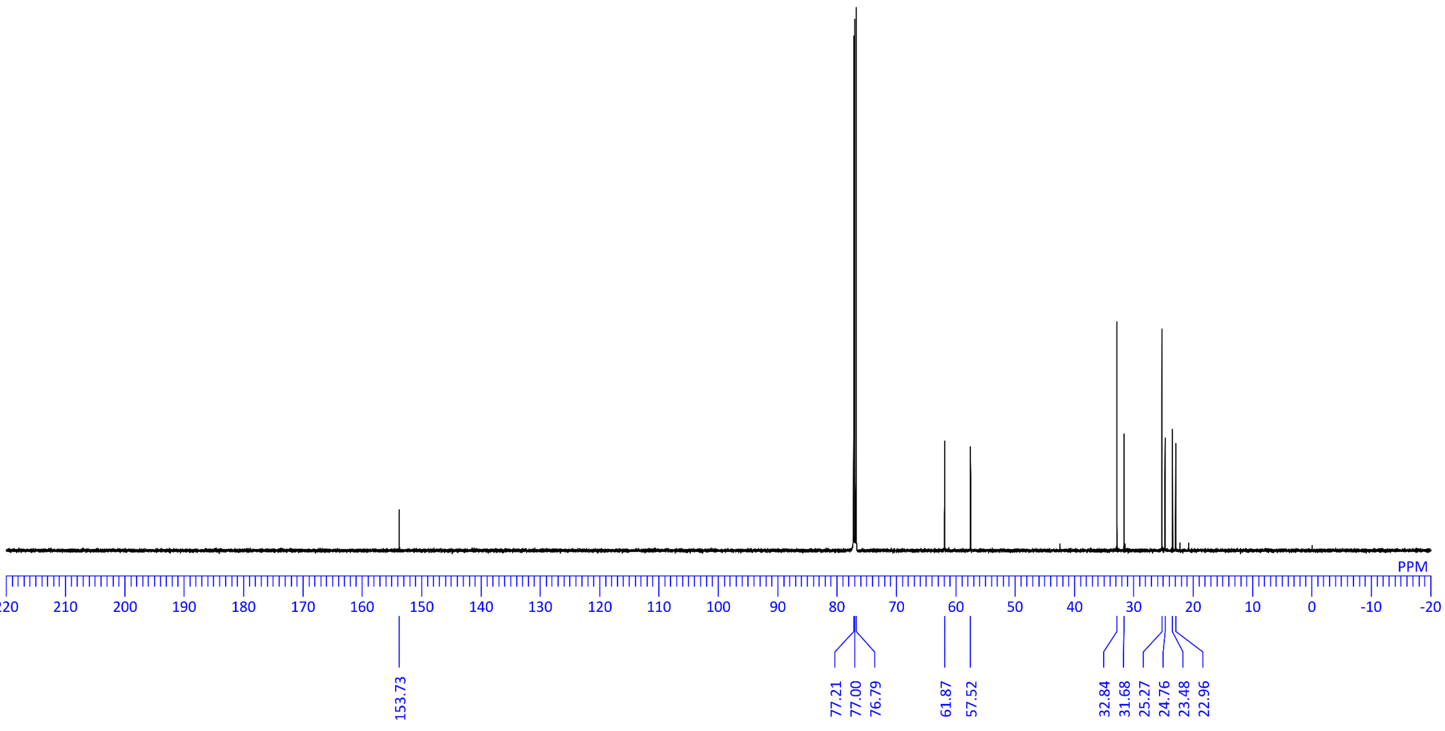
PPM
220
210
200
190
180
170
160
150
140
130
120
110
100
90
80
70
60
50
40
30
20
10
0
-10
-20
153.73
77.21
77.00
76.79
61.87
57.52
32.84
31.68
25.27
24.76
23.48
22.96

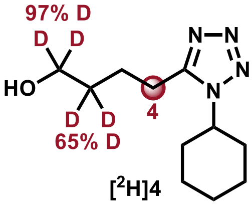
97% D
D D
HO
N–N
N
N
N
4
D D
65% D

$[^2H]4$

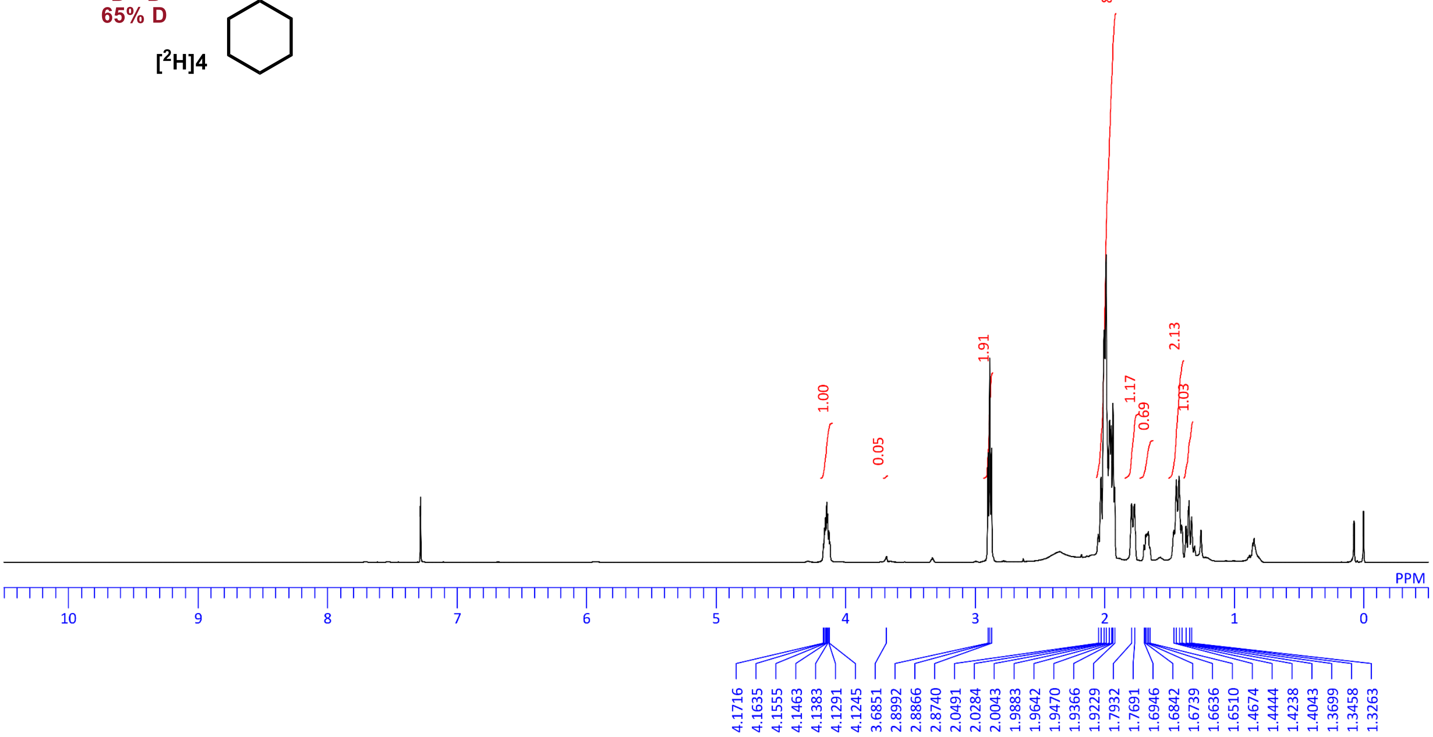
8.41
1.91
1.00
0.05
1.17
0.69
2.13
1.03
PPM
10 9 8 7 6 5 4 3 2 1 0
4.1716 4.1635 4.1555 4.1463 4.1383 4.1291 4.1245 3.6851 2.8992 2.8866 2.8740 2.0491 2.0284 2.0043 1.9883 1.9642 1.9470 1.9366 1.9229 1.7932 1.7691 1.6946 1.6842 1.6739 1.6636 1.6510 1.4674 1.4444 1.4238 1.4043 1.3699 1.3458 1.3263

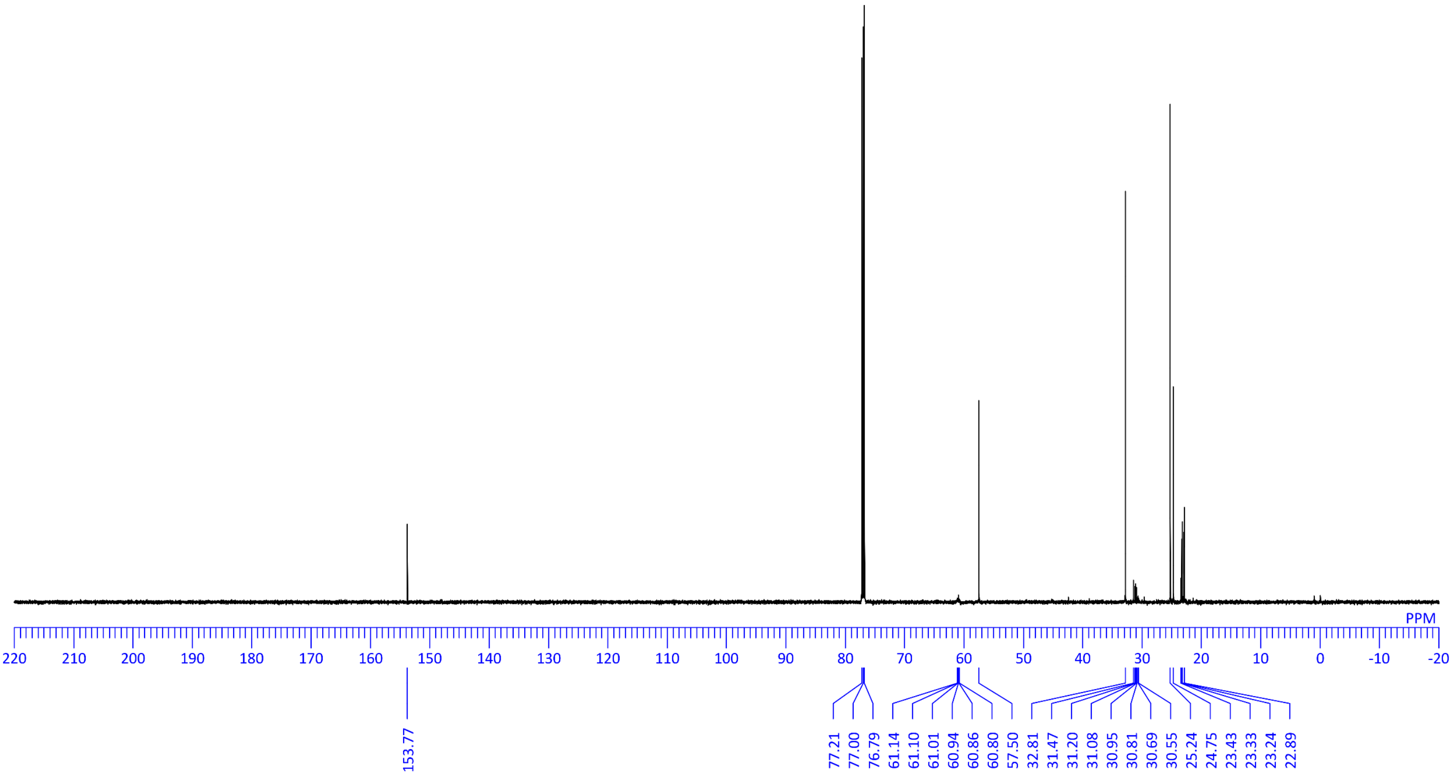
PPM
220 210 200 190 180 170 160 150 140 130 120 110 100 90 80 70 60 50 40 30 20 10 0 -10 -20
153.77
77.21 77.00 76.79 61.14 61.10 61.01 60.94 60.86 60.80 57.50 32.81 31.47 31.20 31.08 30.95 30.81 30.69 30.55 25.24 24.75 23.43 23.33 23.24 22.89

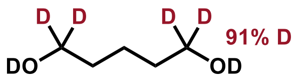

D D D D
91% D
DO
OD

[2H]5

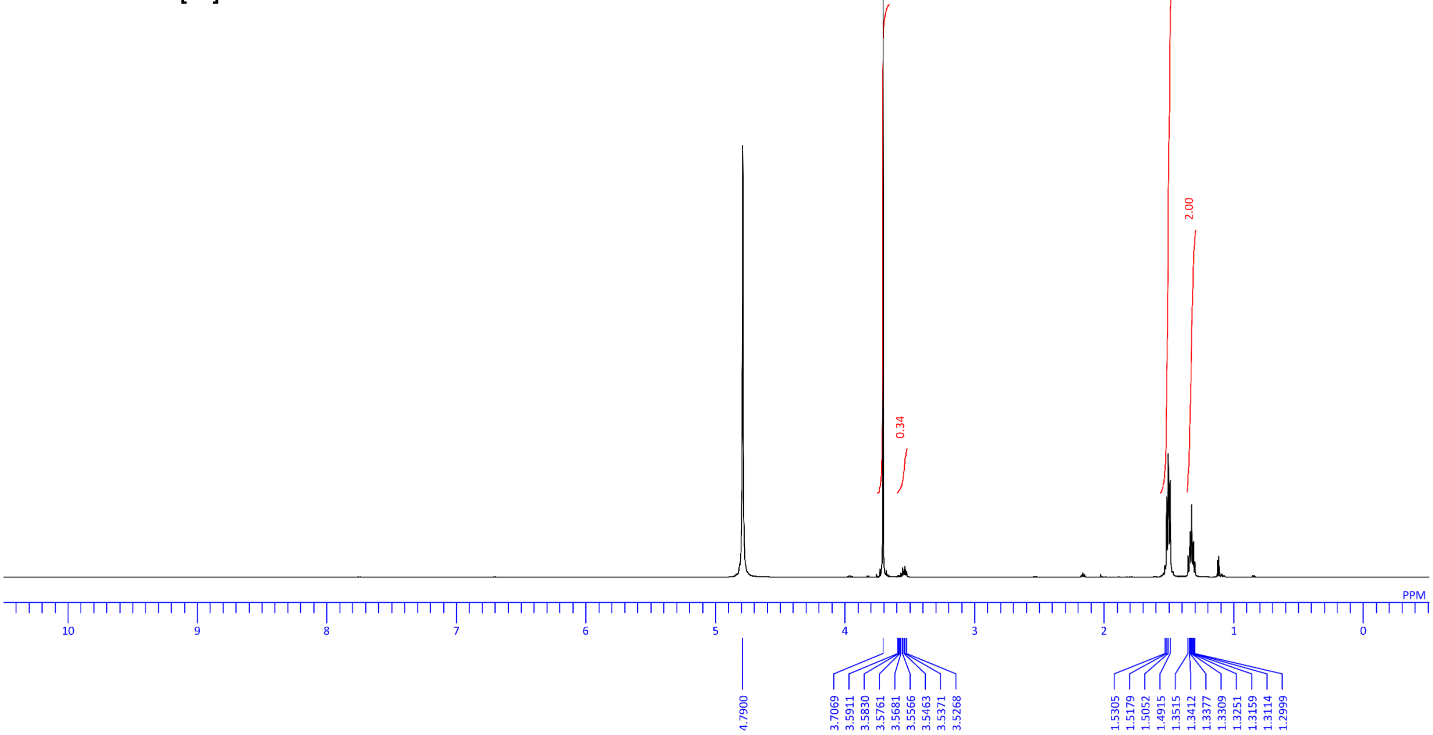

3.72
0.34
3.99
2.00
PPM
10
9
8
7
6
5
4
3
2
1
0
4.7900
3.7069
3.5911
3.5830
3.5761
3.5681
3.5566
3.5463
3.5371
3.5268
1.5305
1.5179
1.5052
1.4915
1.3515
1.3412
1.3377
1.3309
1.3251
1.3159
1.3114
1.2999

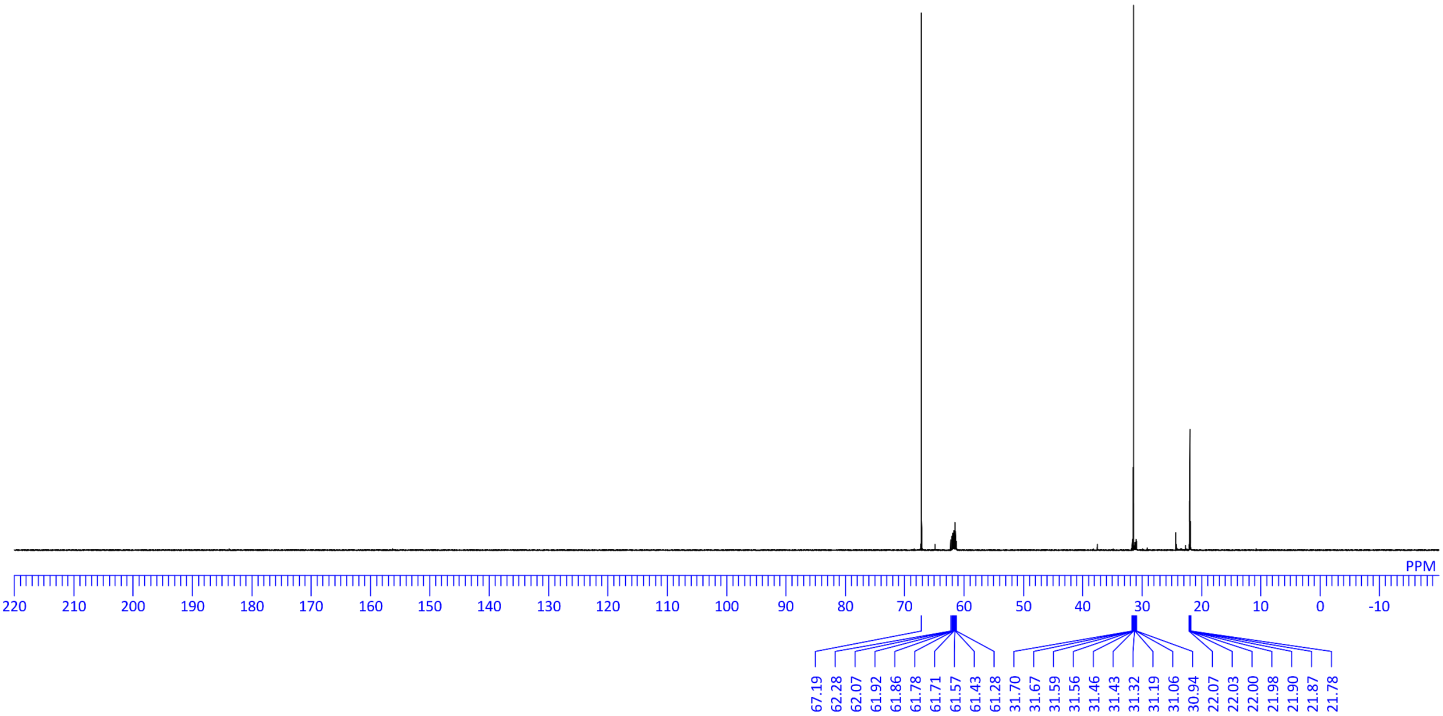

PPM
220
210
200
190
180
170
160
150
140
130
120
110
100
90
80
70
60
50
40
30
20
10
0
-10
67.19
62.28
62.07
61.92
61.86
61.78
61.71
61.57
61.43
61.28
31.70
31.67
31.59
31.56
31.46
31.43
31.32
31.19
31.06
30.94
22.07
22.03
22.00
21.98
21.90
21.87
21.78

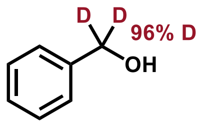
D D
96% D
OH

[^{2}H]6

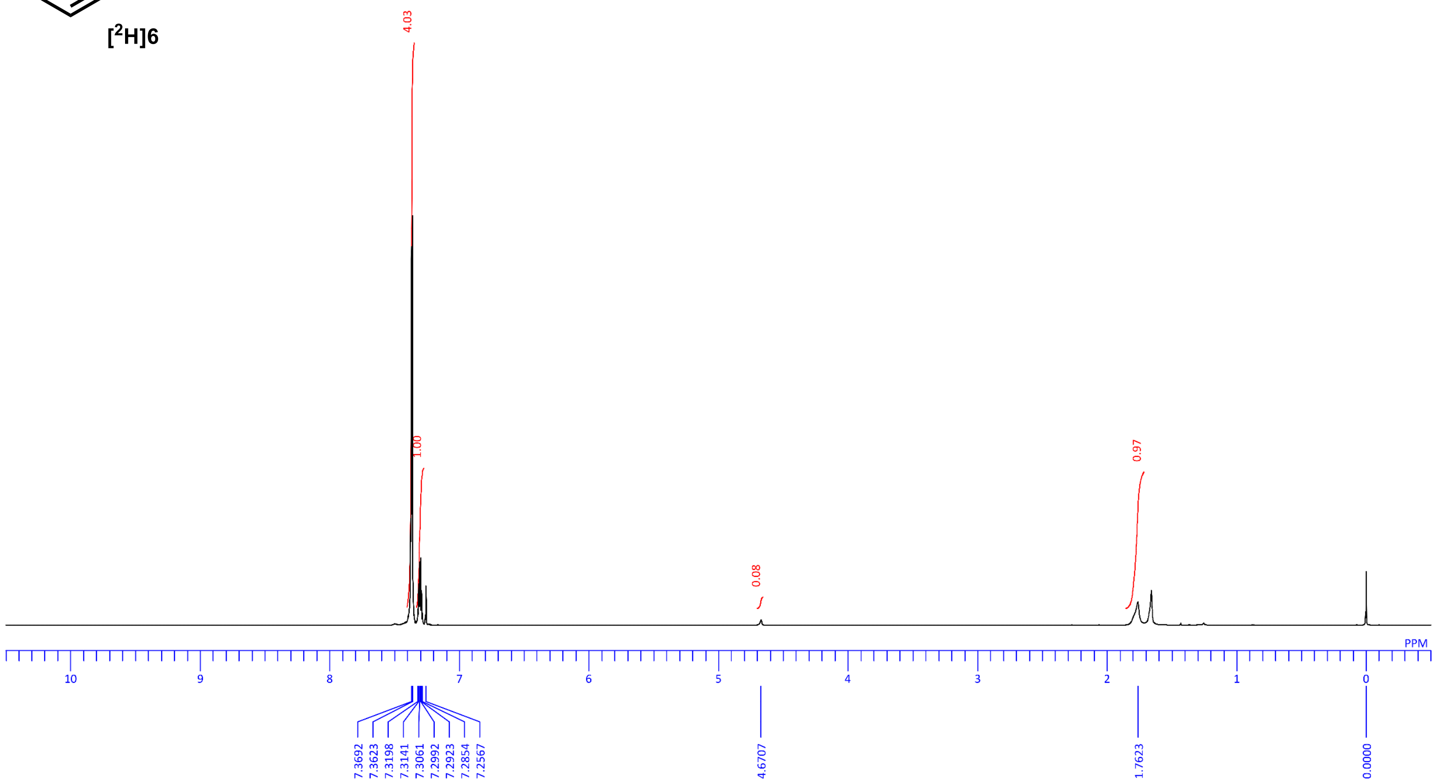
4.03
1.00
0.08
0.97
PPM
10
9
8
7
6
5
4
3
2
1
0
7.3692
7.3623
7.3198
7.3141
7.3061
7.2992
7.2923
7.2854
7.2567
4.6707
1.7623
0.0000

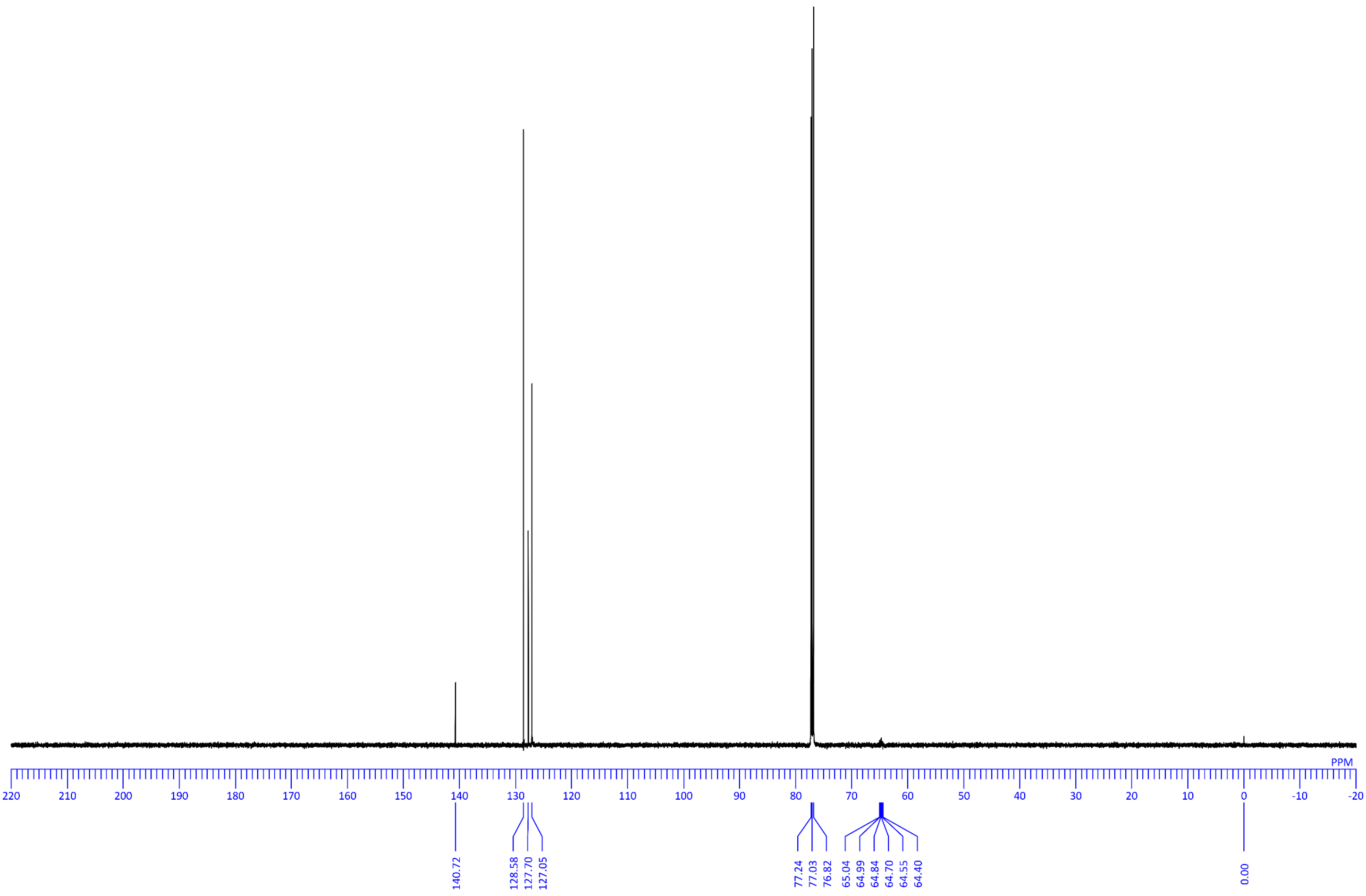
PPM
220
210
200
190
180
170
160
150
140
130
120
110
100
90
80
70
60
50
40
30
20
10
0
-10
-20
140.72
128.58
127.70
127.05
77.24
77.03
76.82
65.04
64.99
64.84
64.70
64.55
64.40
0.00

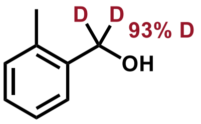
D D
93% D
OH

[2H]7

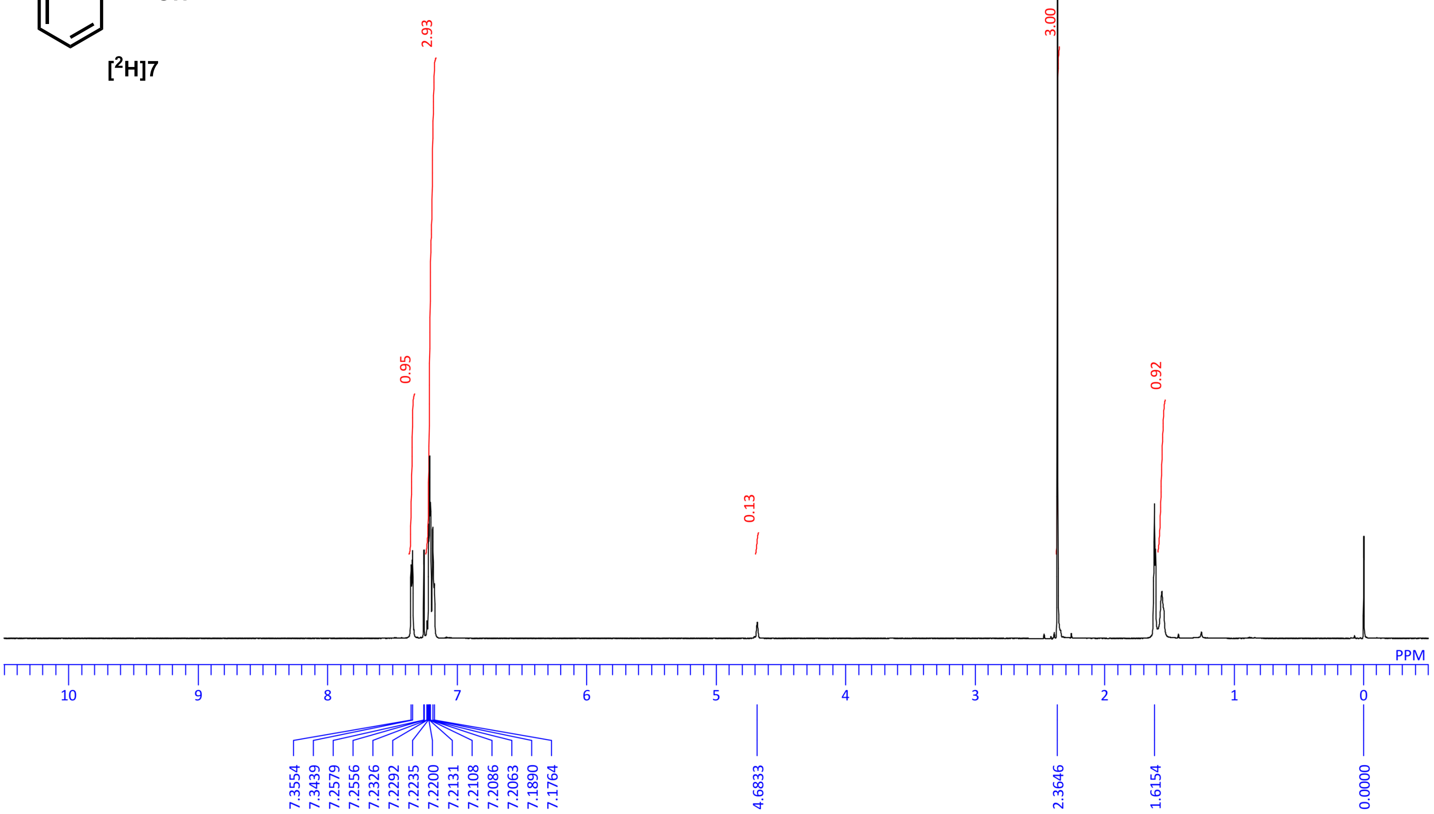
0.95
2.93
0.13
3.00
0.92
PPM
10 9 8 7 6 5 4 3 2 1 0
7.3554
7.3439
7.2579
7.2556
7.2326
7.2292
7.2235
7.2200
7.2131
7.2108
7.2086
7.2063
7.1890
7.1764
4.6833
2.3646
1.6154
0.0000

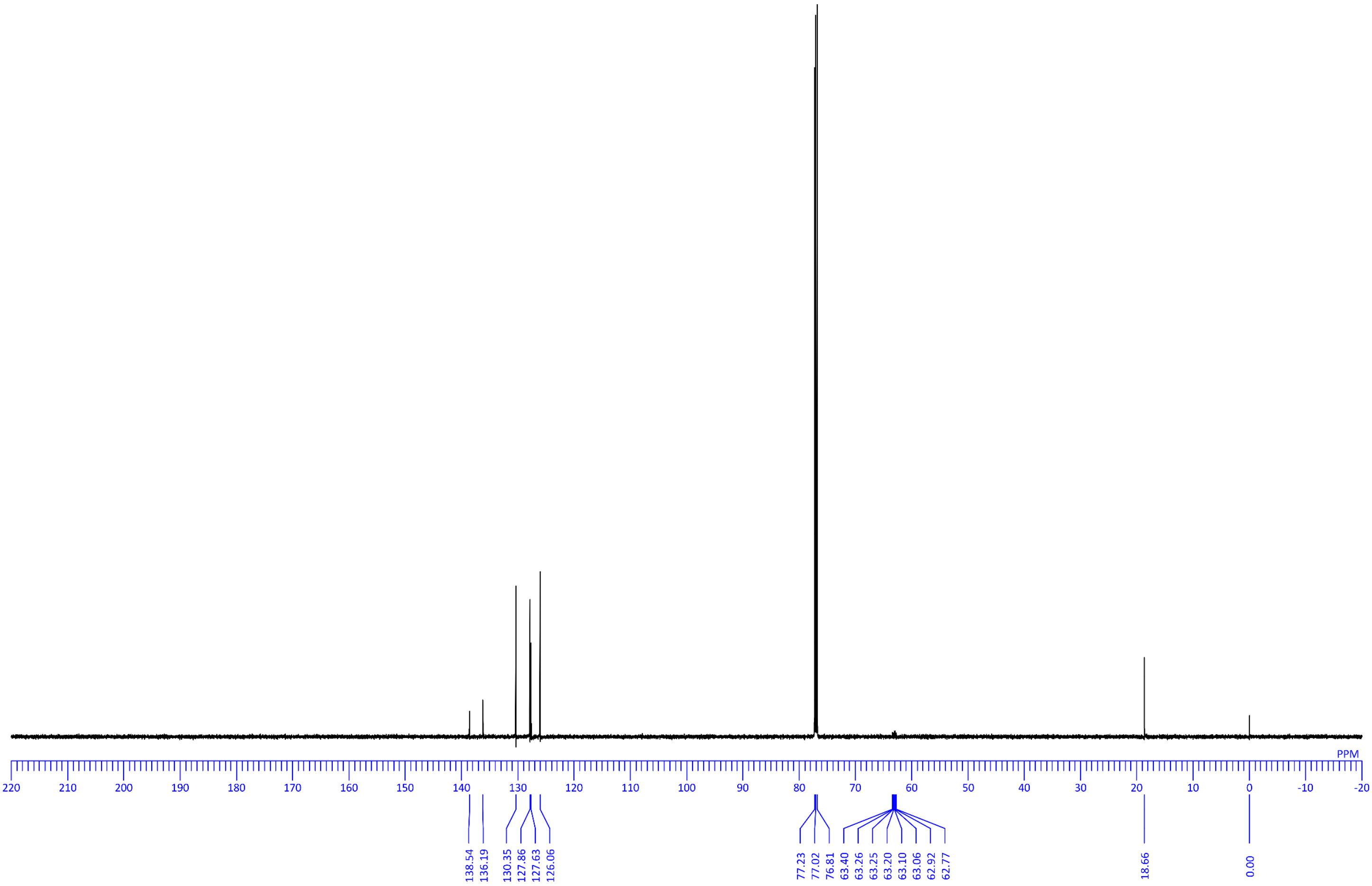
PPM
220 210 200 190 180 170 160 150 140 130 120 110 100 90 80 70 60 50 40 30 20 10 0 -10 -20
138.54
136.19
130.35
127.86
127.63
126.06
77.23
77.02
76.81
63.40
63.26
63.25
63.20
63.10
63.06
62.92
62.77
18.66
0.00

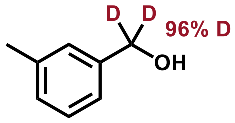
D
D
96% D
OH

[²H]8

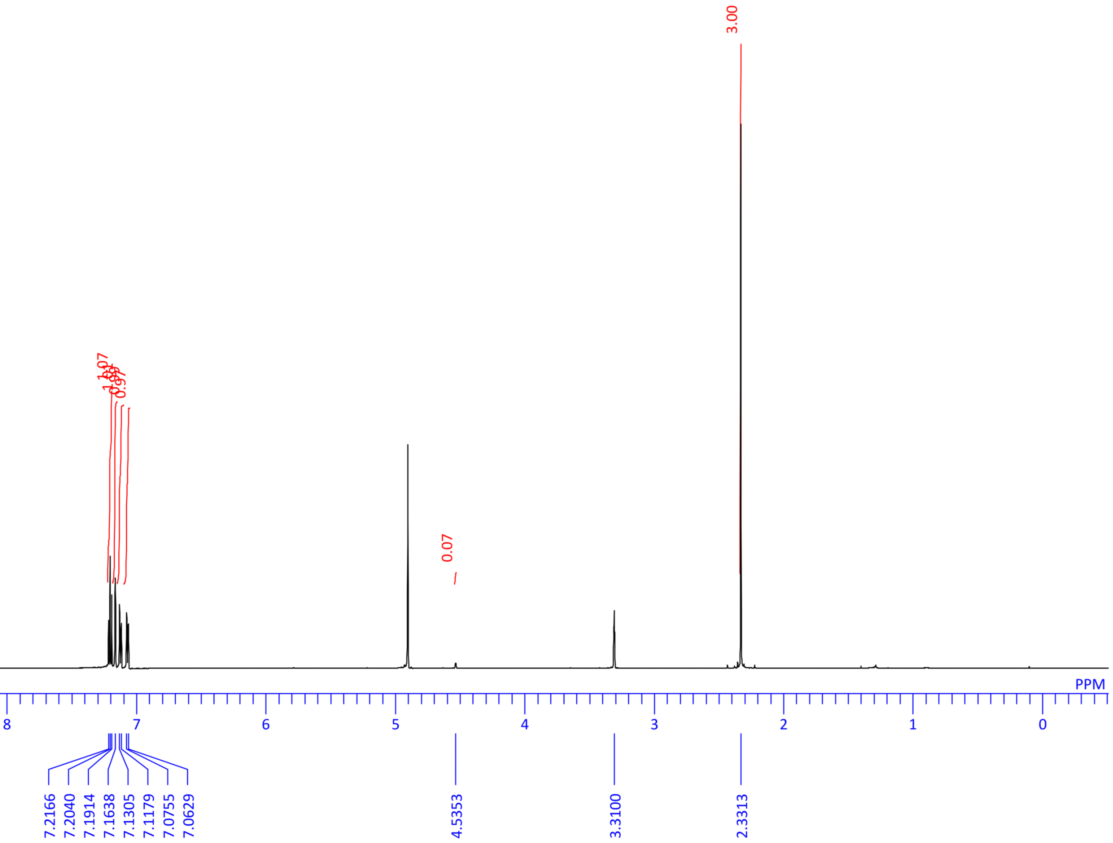
3.00
1.07
0.07
PPM
10
9
8
7
6
5
4
3
2
1
0
7.2166
7.2040
7.1914
7.1638
7.1305
7.1179
7.0755
7.0629
4.5353
3.3100
2.3313

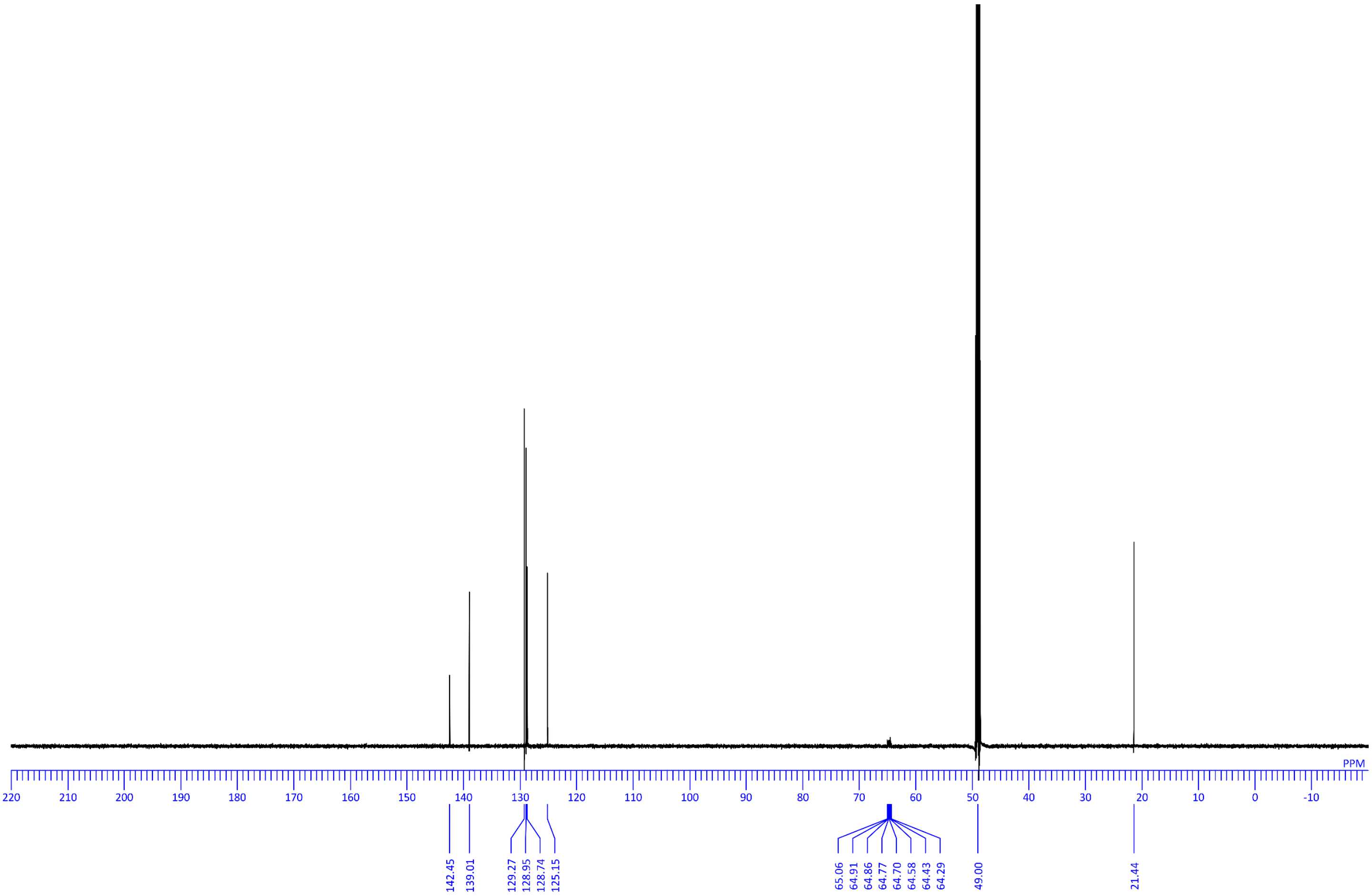
PPM
220
210
200
190
180
170
160
150
140
130
120
110
100
90
80
70
60
50
40
30
20
10
0
-10
142.45
139.01
129.27
128.95
128.74
125.15
65.06
64.91
64.86
64.77
64.70
64.58
64.43
64.29
49.00
21.44

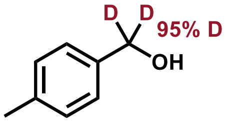
D D
95% D
OH

[^{2}H]9

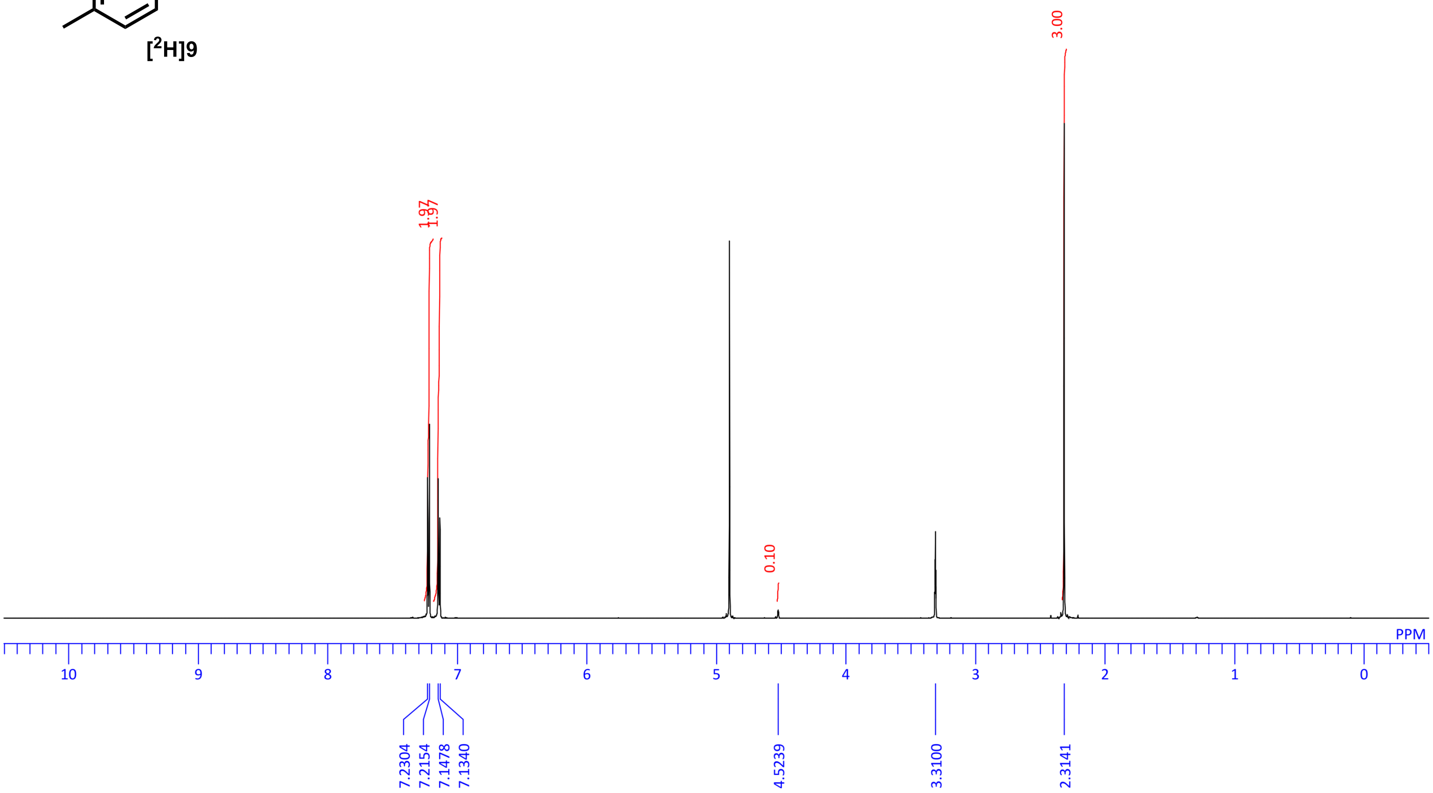
1.97
1.97
0.10
3.00
PPM
10
9
8
7
6
5
4
3
2
1
0
7.2304
7.2154
7.1478
7.1340
4.5239
3.3100
2.3141

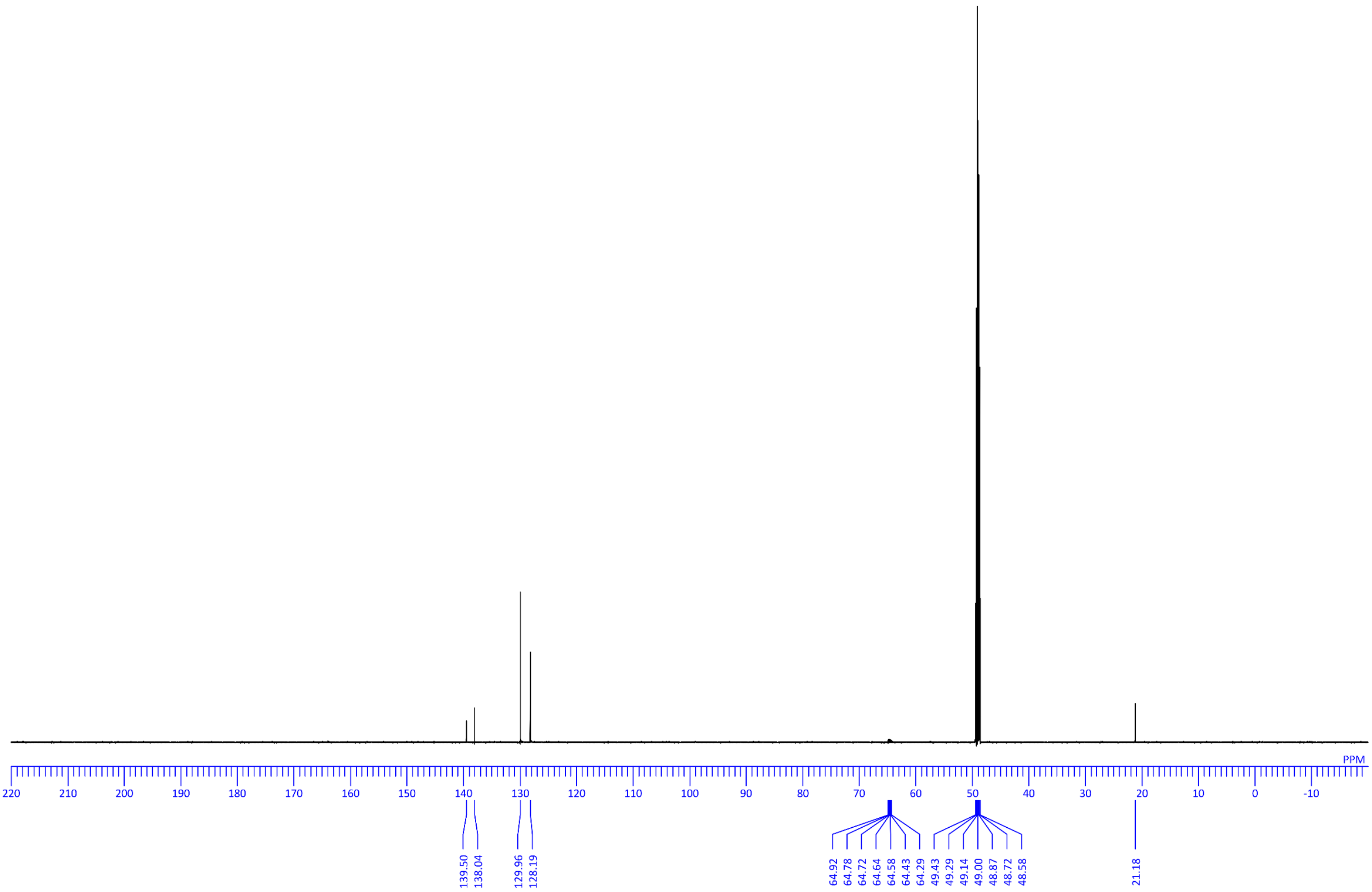
PPM
220
210
200
190
180
170
160
150
140
130
120
110
100
90
80
70
60
50
40
30
20
10
0
-10
139.50
138.04
129.96
128.19
64.92
64.78
64.72
64.64
64.58
64.43
64.29
49.43
49.29
49.14
49.00
48.87
48.72
48.58
21.18

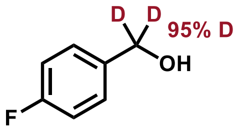

D
D
95% D
OH
F

[^{2}H]10

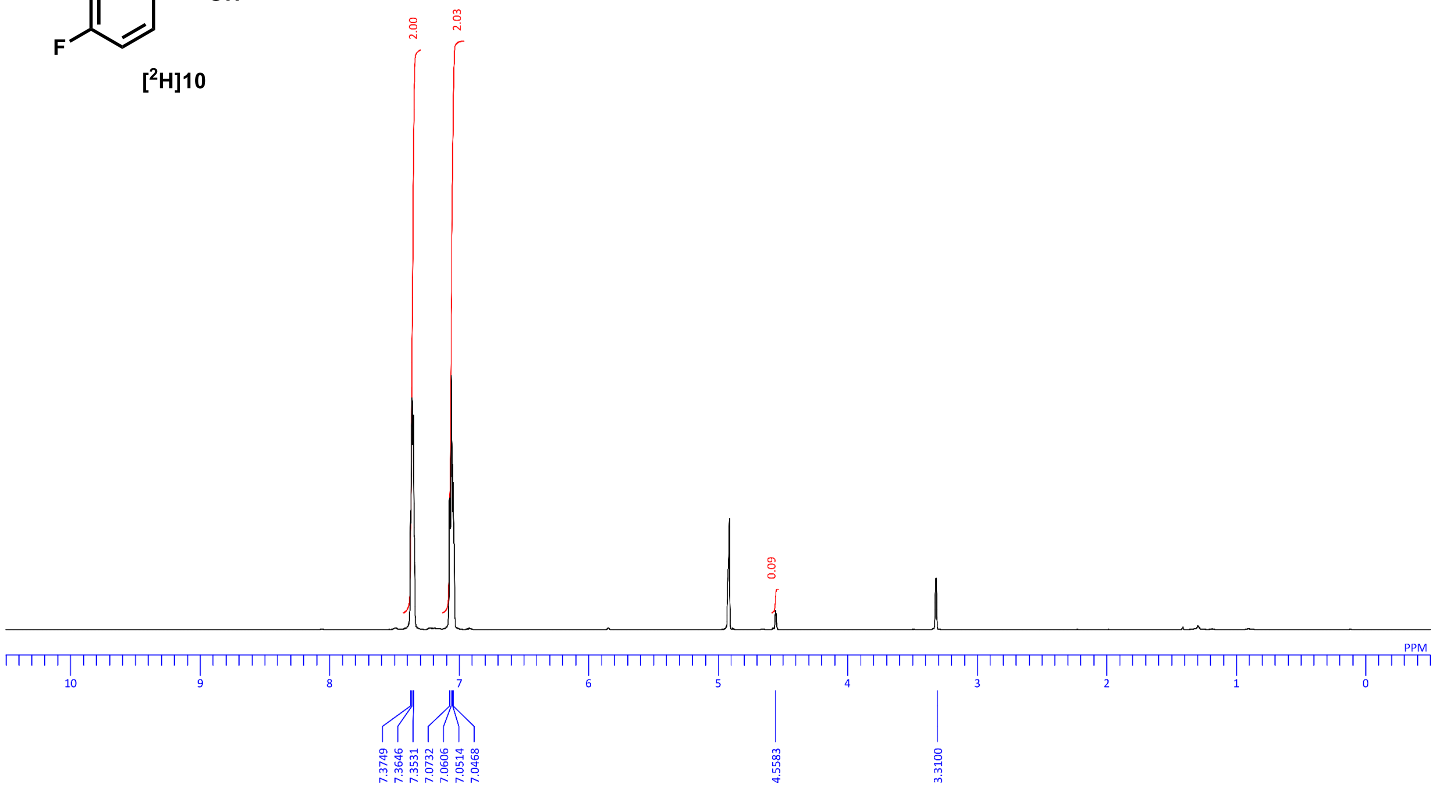

2.00
2.03
0.09
PPM
10
9
8
7
6
5
4
3
2
1
0
7.3749
7.3646
7.3531
7.0732
7.0606
7.0514
7.0468
4.5583
3.3100

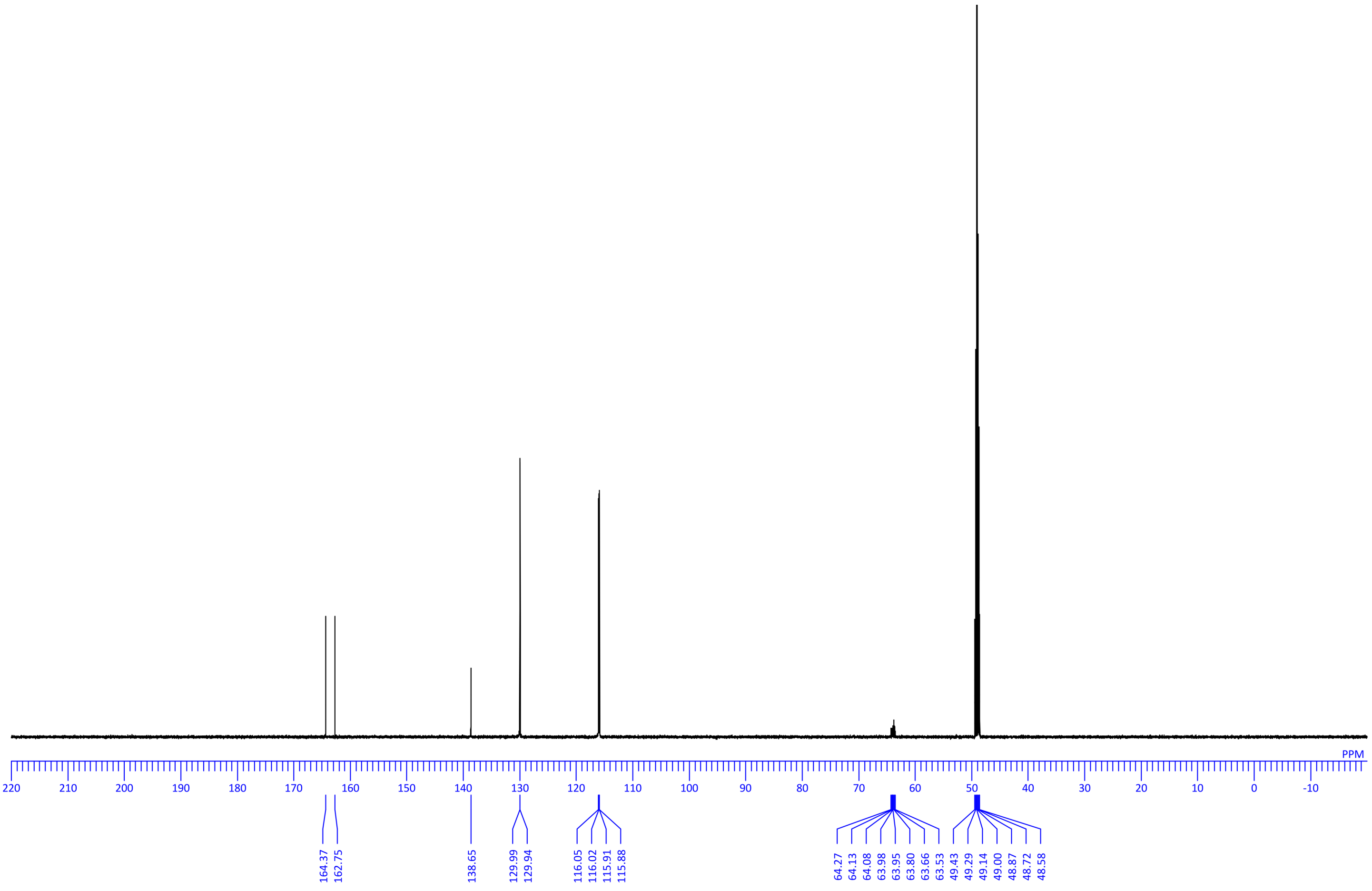

PPM
220
210
200
190
180
170
160
150
140
130
120
110
100
90
80
70
60
50
40
30
20
10
0
-10
164.37
162.75
138.65
129.99
129.94
116.05
116.02
115.91
115.88
64.27
64.13
64.08
63.98
63.95
63.80
63.66
63.53
49.43
49.29
49.14
49.00
48.87
48.72
48.58

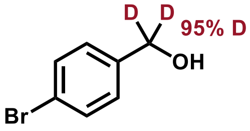
D
D
95% D
OH
Br

[^{2}H]11

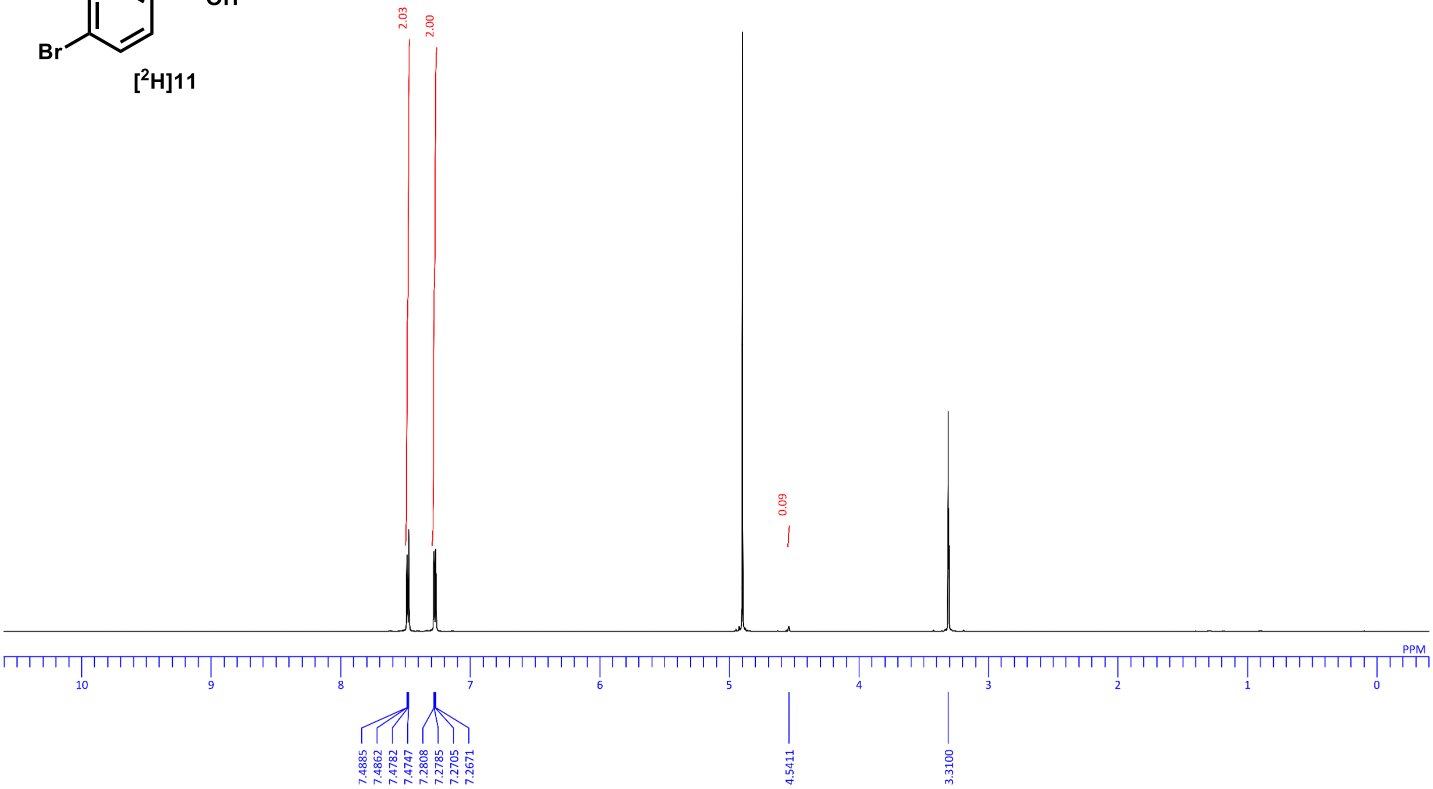
2.03
2.00
0.09
PPM
10
9
8
7
6
5
4
3
2
1
0
7.4885
7.4862
7.4782
7.4747
7.2808
7.2785
7.2705
7.2671
4.5411
3.3100

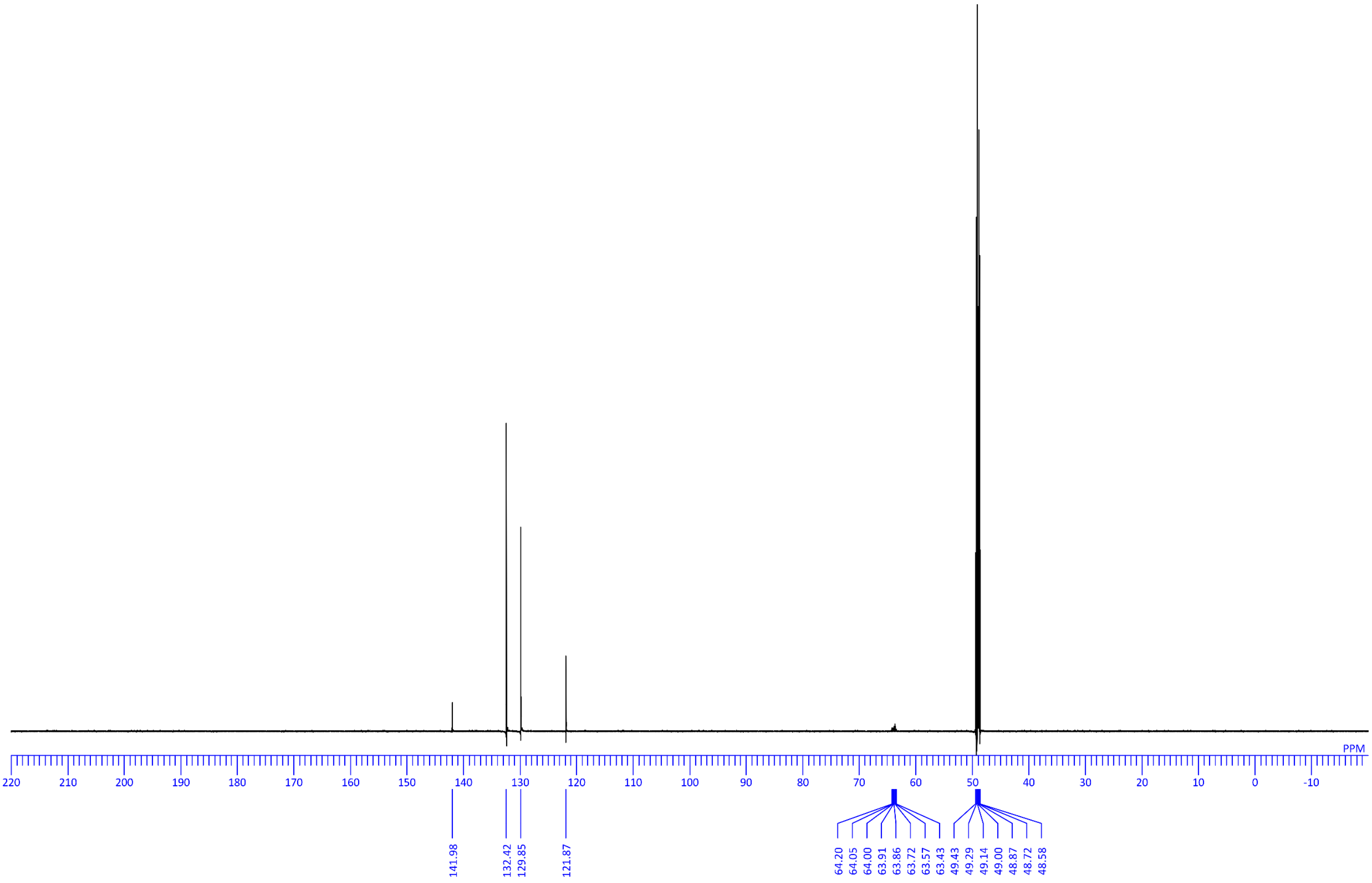
PPM
220
210
200
190
180
170
160
150
140
130
120
110
100
90
80
70
60
50
40
30
20
10
0
-10
141.98
132.42
129.85
121.87
64.20
64.05
64.00
63.91
63.86
63.72
63.57
63.43
49.43
49.29
49.14
49.00
48.87
48.72
48.58

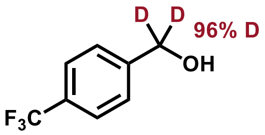
D D
96% D
OH
F3C

[2H]12

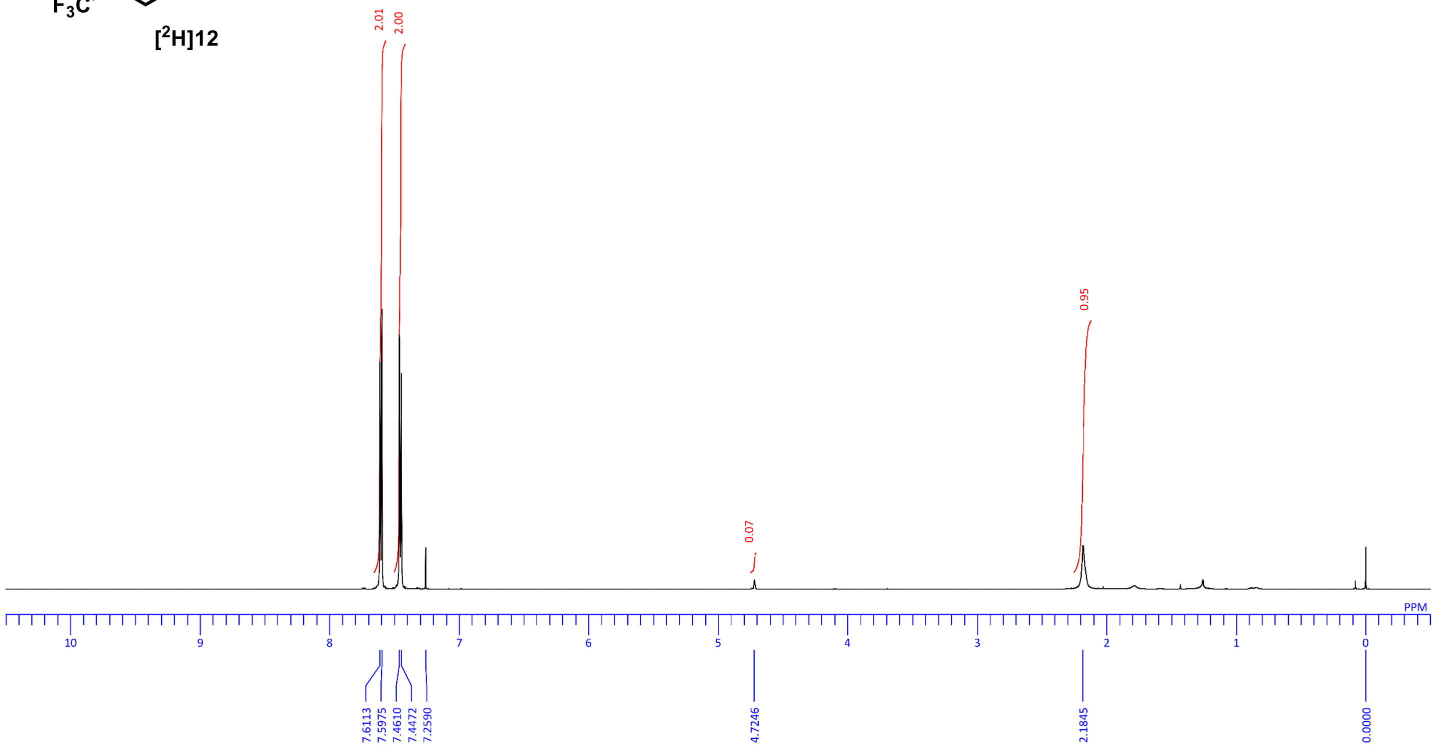
2.01
2.00
0.07
0.95
PPM
10 9 8 7 6 5 4 3 2 1 0
7.6113
7.5975
7.4610
7.4472
7.2590
4.7246
2.1845
0.0000

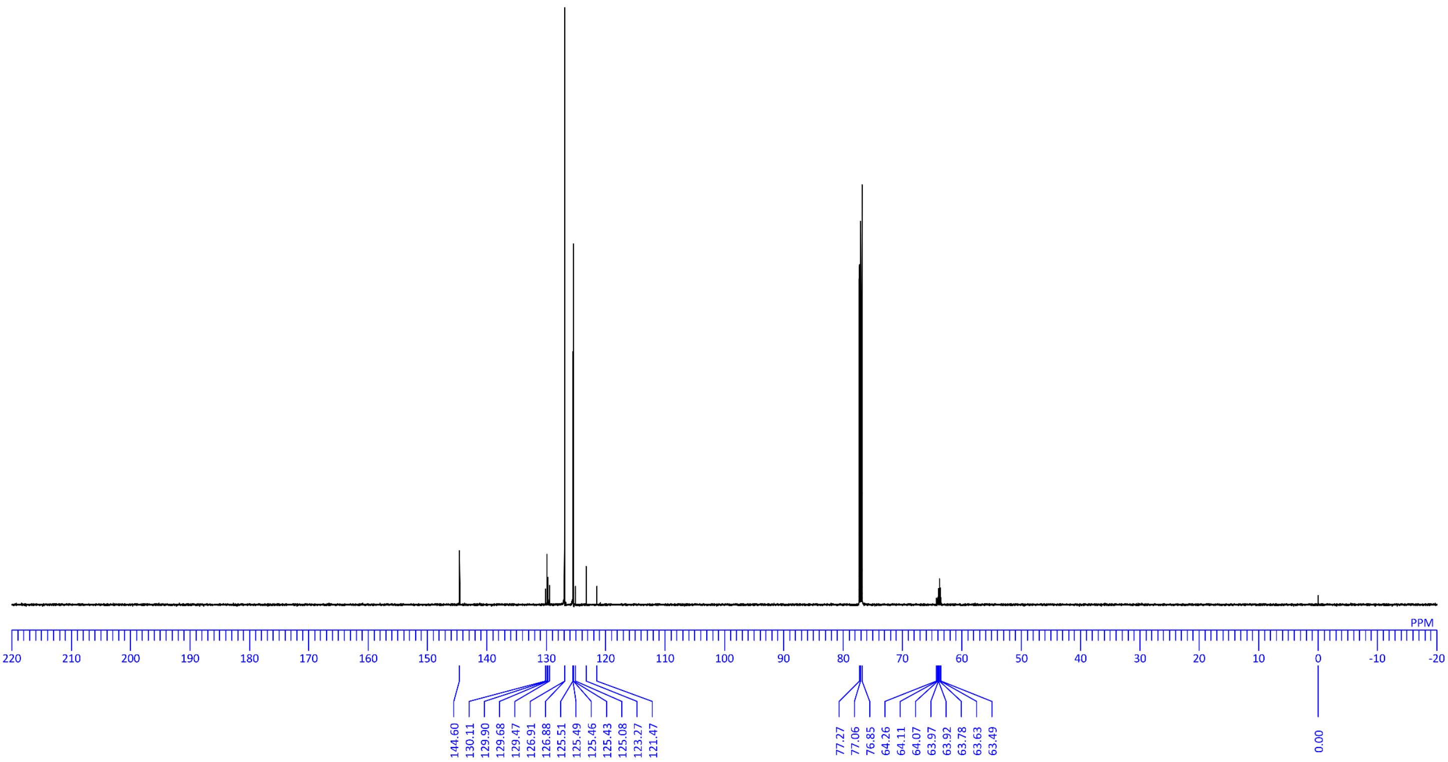
PPM
220 210 200 190 180 170 160 150 140 130 120 110 100 90 80 70 60 50 40 30 20 10 0 -10 -20
144.60
130.11
129.90
129.68
129.47
126.91
126.88
125.51
125.49
125.46
125.43
125.08
123.27
121.47
77.27
77.06
76.85
64.26
64.11
64.07
63.97
63.92
63.78
63.63
63.49
0.00

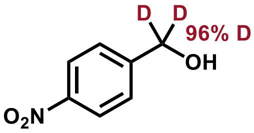
D D
96% D
OH
O2N

[^{2}H]13

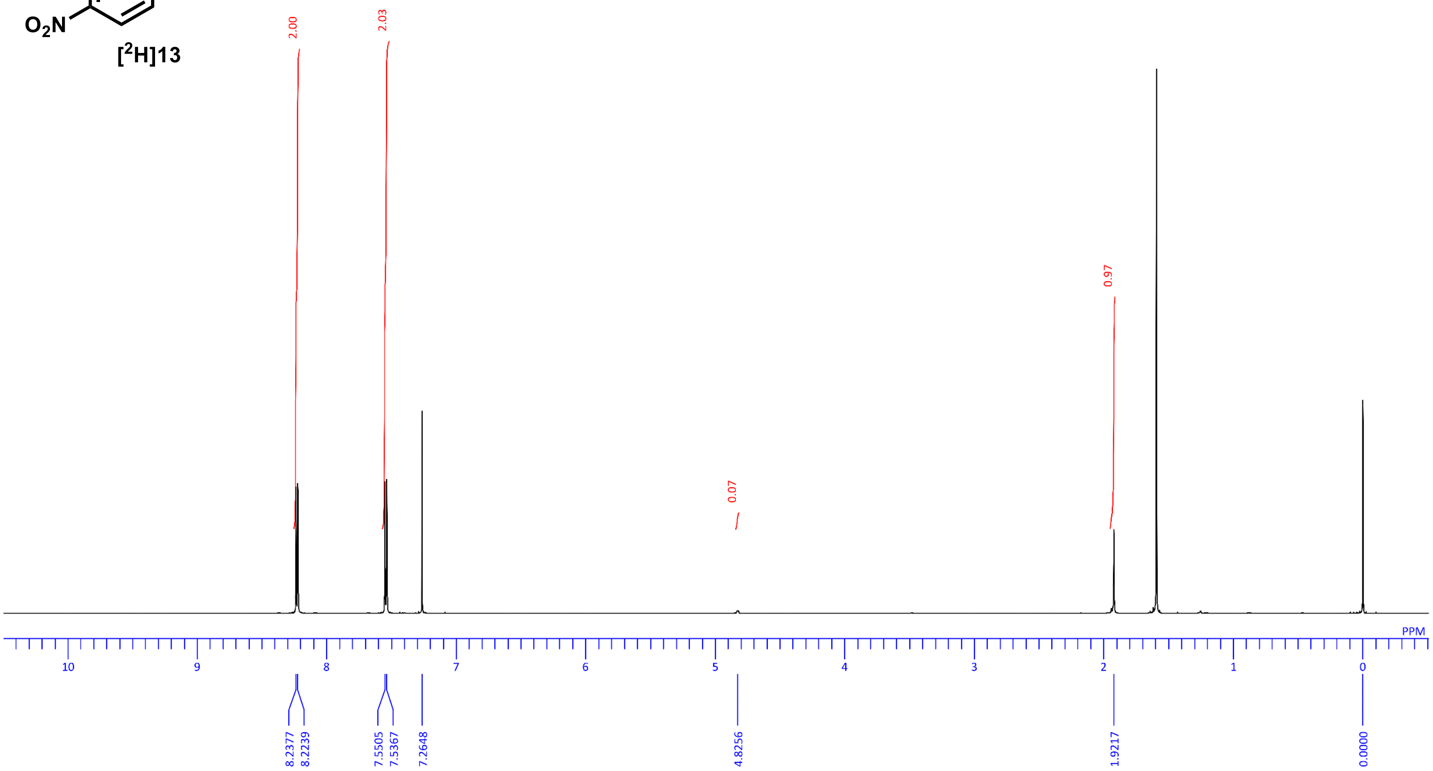
2.00
2.03
0.07
0.97
PPM
10
9
8
7
6
5
4
3
2
1
0
8.2377
8.2239
7.5505
7.5367
7.2648
4.8256
1.9217
0.0000

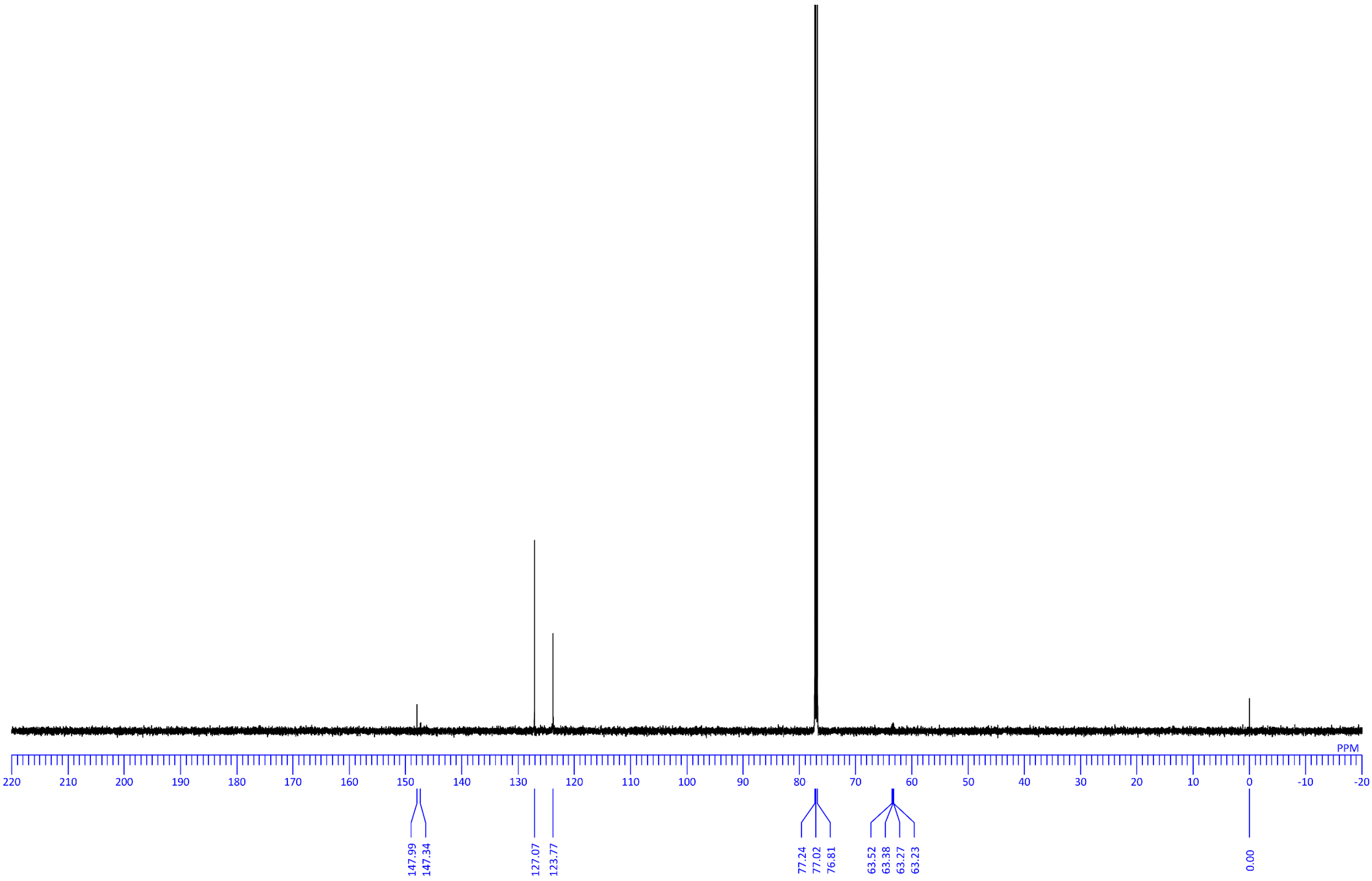
PPM
220
210
200
190
180
170
160
150
140
130
120
110
100
90
80
70
60
50
40
30
20
10
0
-10
-20
147.99
147.34
127.07
123.77
77.24
77.02
76.81
63.52
63.38
63.27
63.23
0.00

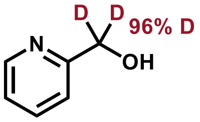
D D
96% D
N
OH

[^{2}H]14

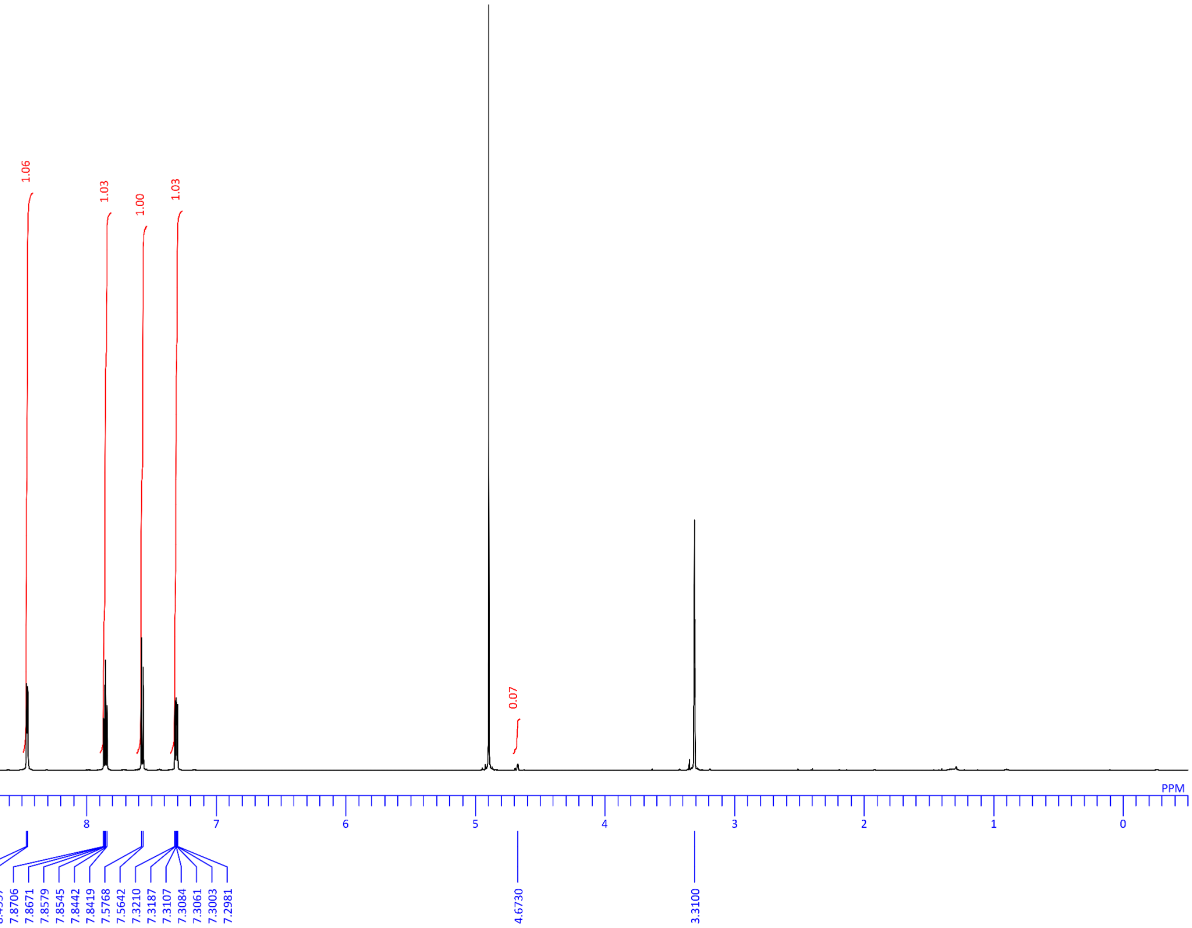
1.06
1.03
1.00
1.03
0.07
PPM
10
9
8
7
6
5
4
3
2
1
0
8.4637
8.4557
7.8706
7.8671
7.8579
7.8545
7.8442
7.8419
7.5768
7.5642
7.3210
7.3187
7.3107
7.3084
7.3061
7.3003
7.2981
4.6730
3.3100

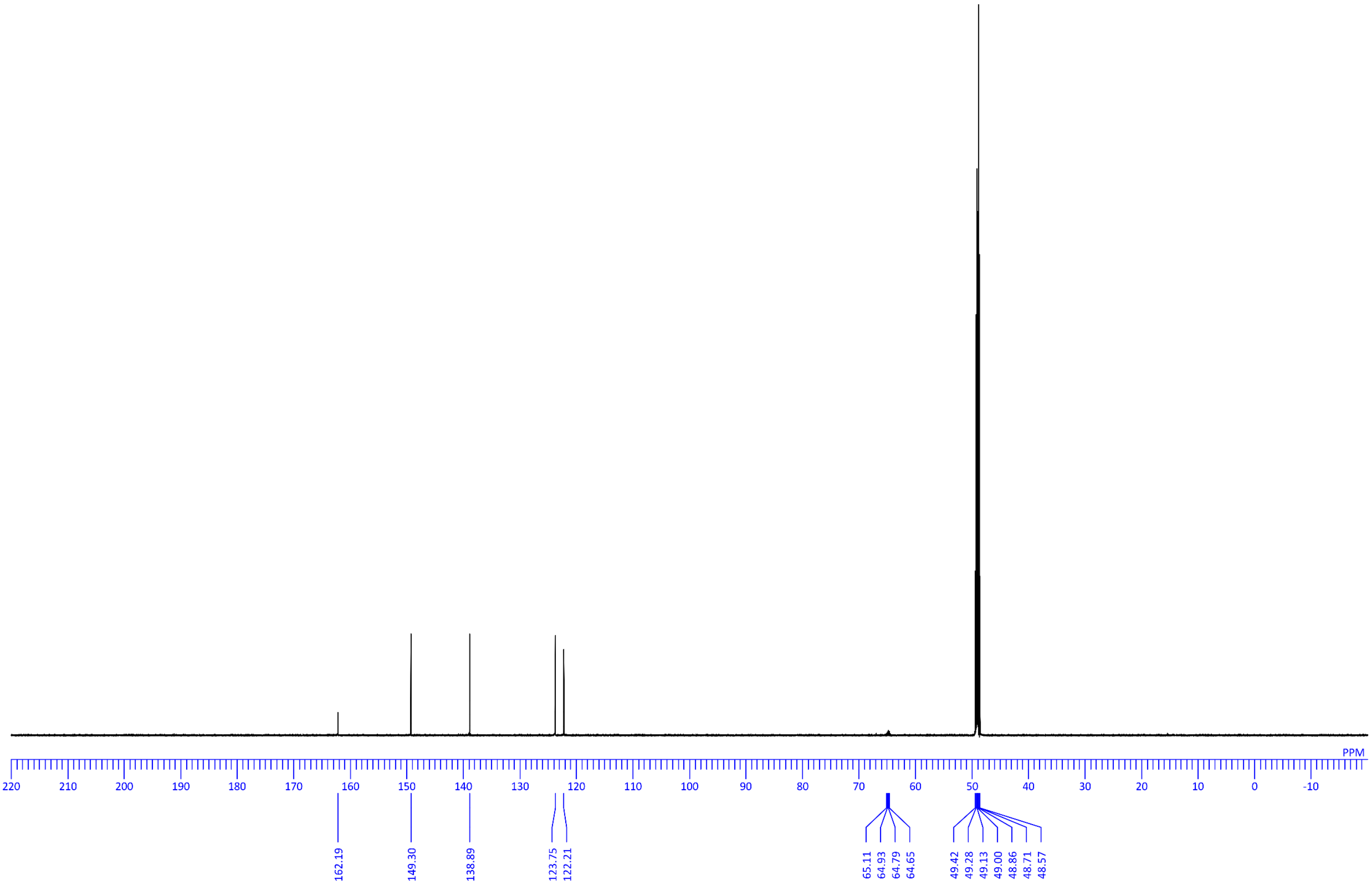
PPM
220
210
200
190
180
170
160
150
140
130
120
110
100
90
80
70
60
50
40
30
20
10
0
-10
162.19
149.30
138.89
123.75
122.21
65.11
64.93
64.79
64.65
49.42
49.28
49.13
49.00
48.86
48.71
48.57

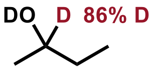
DO
D
86% D

[^{2}H]15

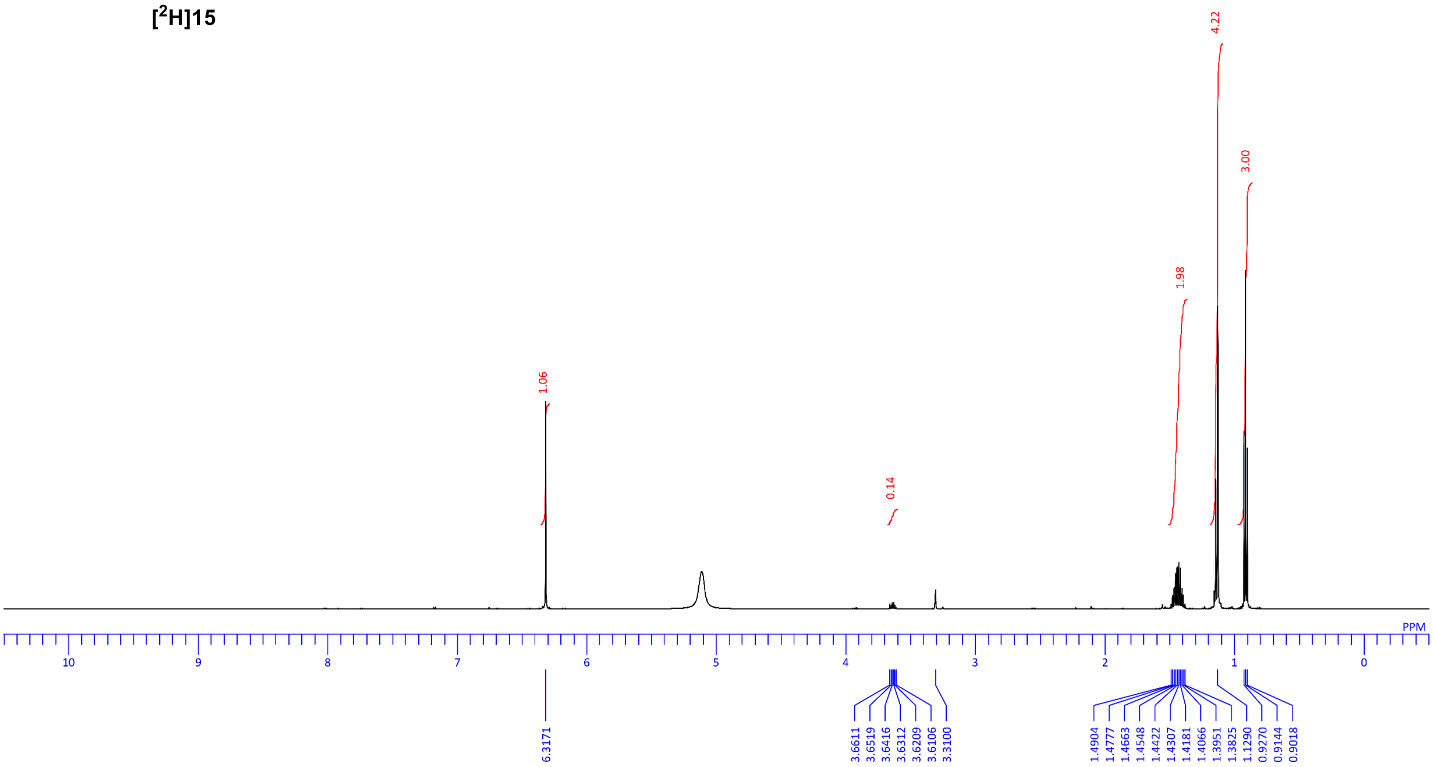
1.06
0.14
1.98
4.22
3.00
PPM
6.3171
3.6611
3.6519
3.6416
3.6312
3.6209
3.6106
3.3100
1.4904
1.4777
1.4663
1.4548
1.4422
1.4307
1.4181
1.4066
1.3951
1.3825
1.1290
0.9270
0.9144
0.9018

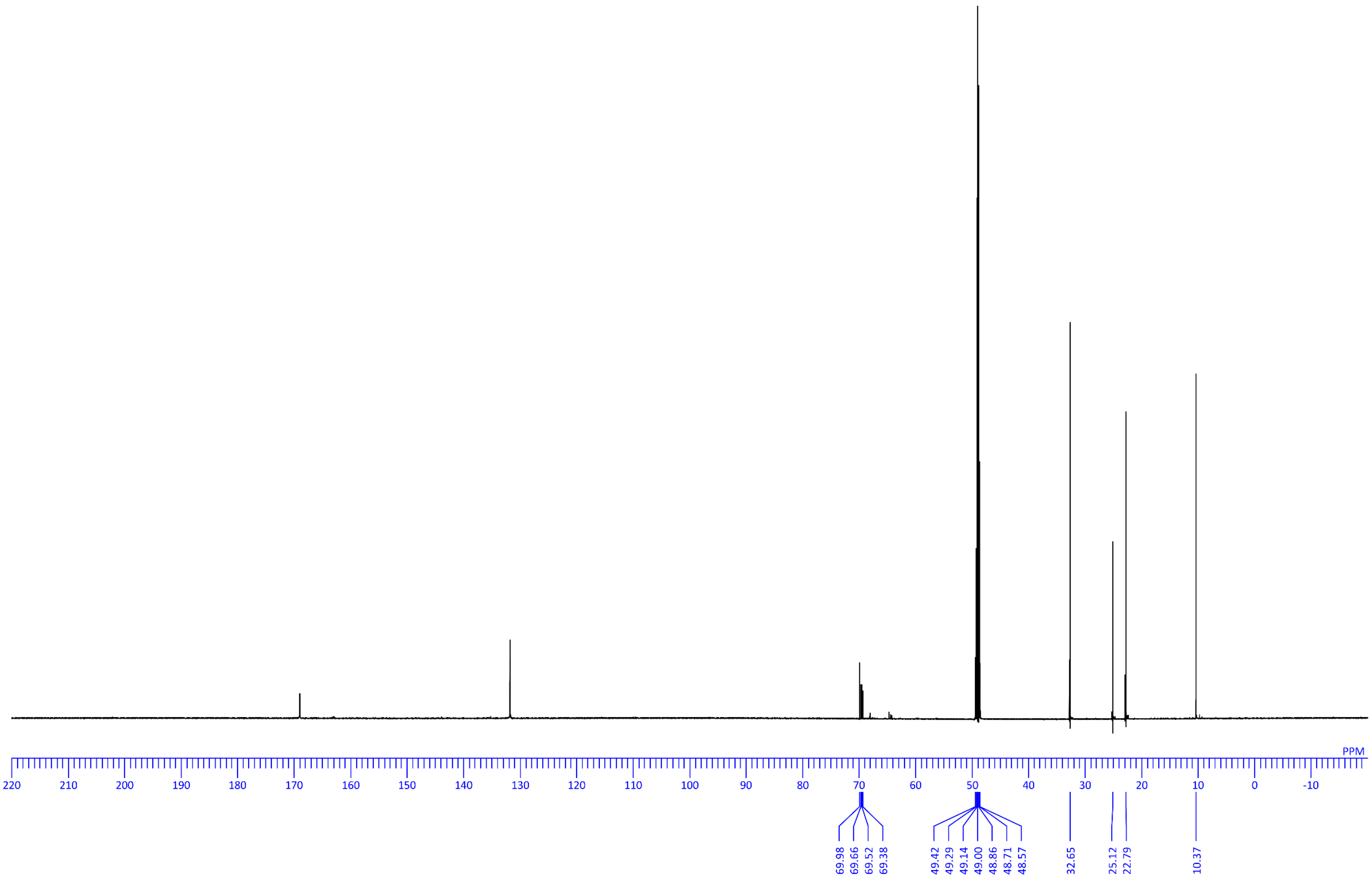
PPM
69.98
69.66
69.52
69.38
49.42
49.29
49.14
49.00
48.86
48.71
48.57
32.65
25.12
22.79
10.37

[^{2}H]16

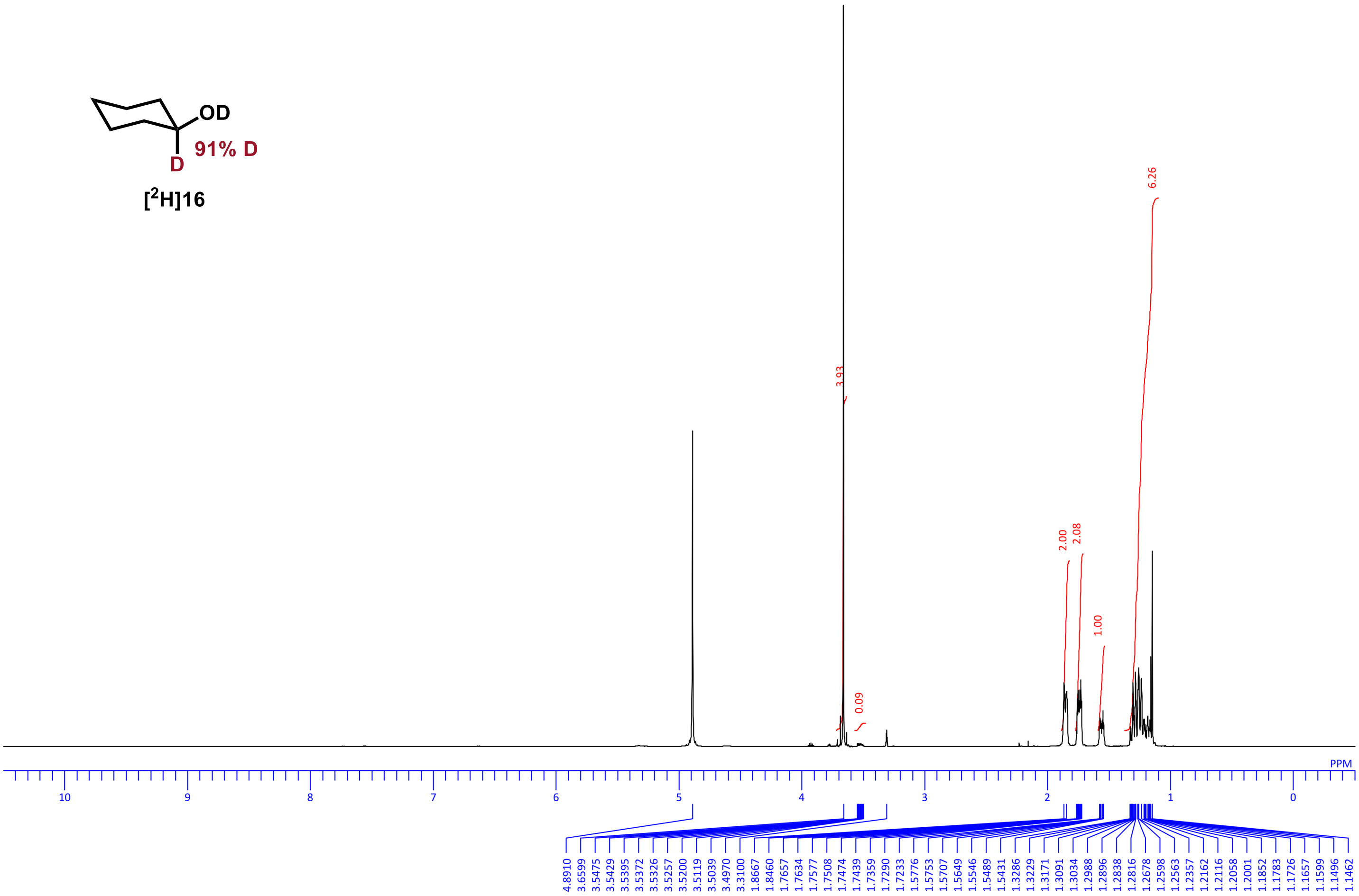
OD
91% D
D
3.93
0.09
2.00
2.08
1.00
6.26
PPM
4.8910
3.6599
3.5475
3.5429
3.5395
3.5372
3.5326
3.5257
3.5200
3.5119
3.5039
3.4970
3.3100
1.8667
1.8460
1.7657
1.7634
1.7577
1.7508
1.7474
1.7439
1.7359
1.7290
1.7233
1.5776
1.5753
1.5707
1.5649
1.5546
1.5489
1.5431
1.3286
1.3229
1.3171
1.3091
1.3034
1.2988
1.2896
1.2838
1.2816
1.2678
1.2598
1.2563
1.2357
1.2162
1.2116
1.2058
1.2001
1.1852
1.1783
1.1726
1.1657
1.1599
1.1496
1.1462

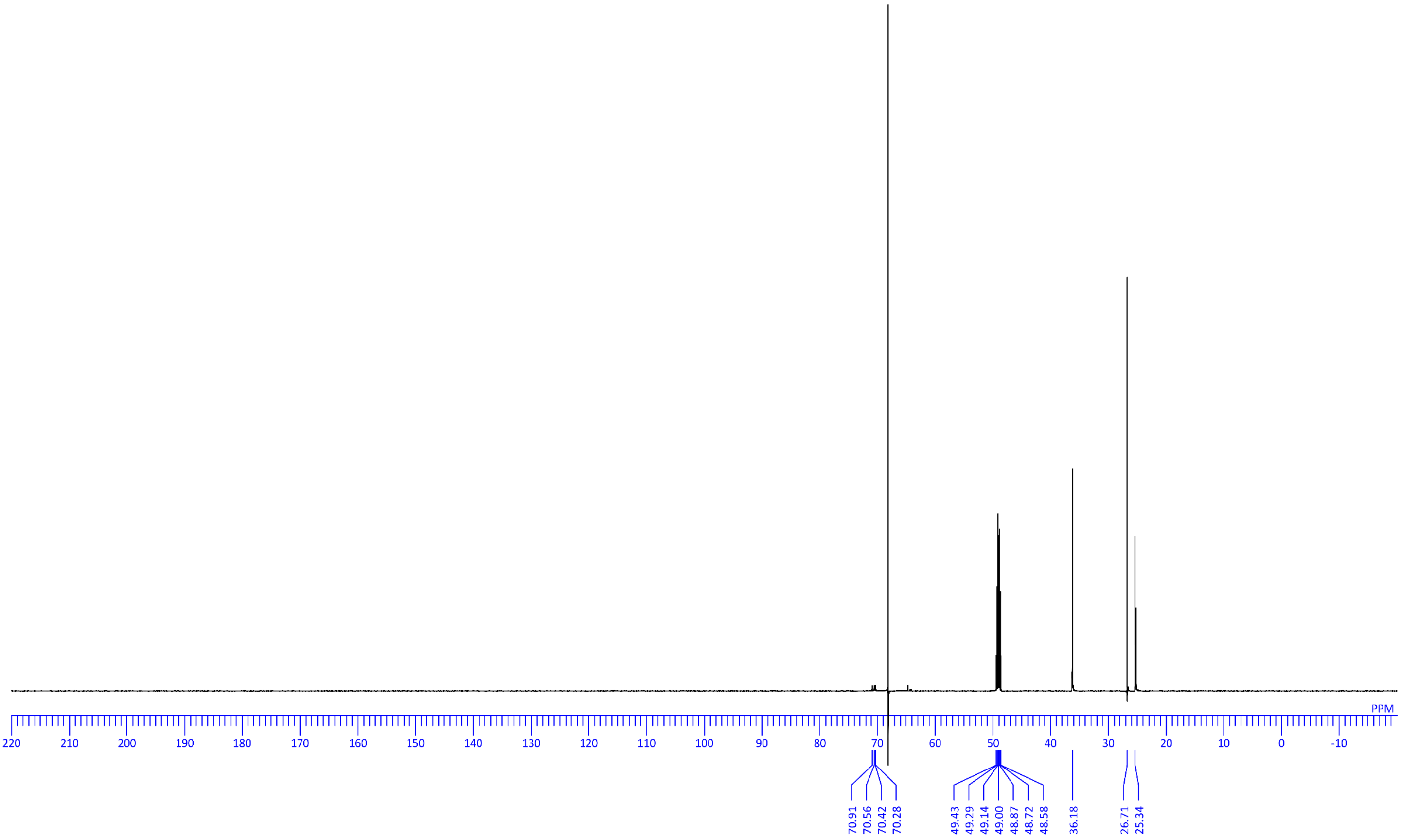
PPM
70.91
70.56
70.42
70.28
49.43
49.29
49.14
49.00
48.87
48.72
48.58
36.18
26.71
25.34

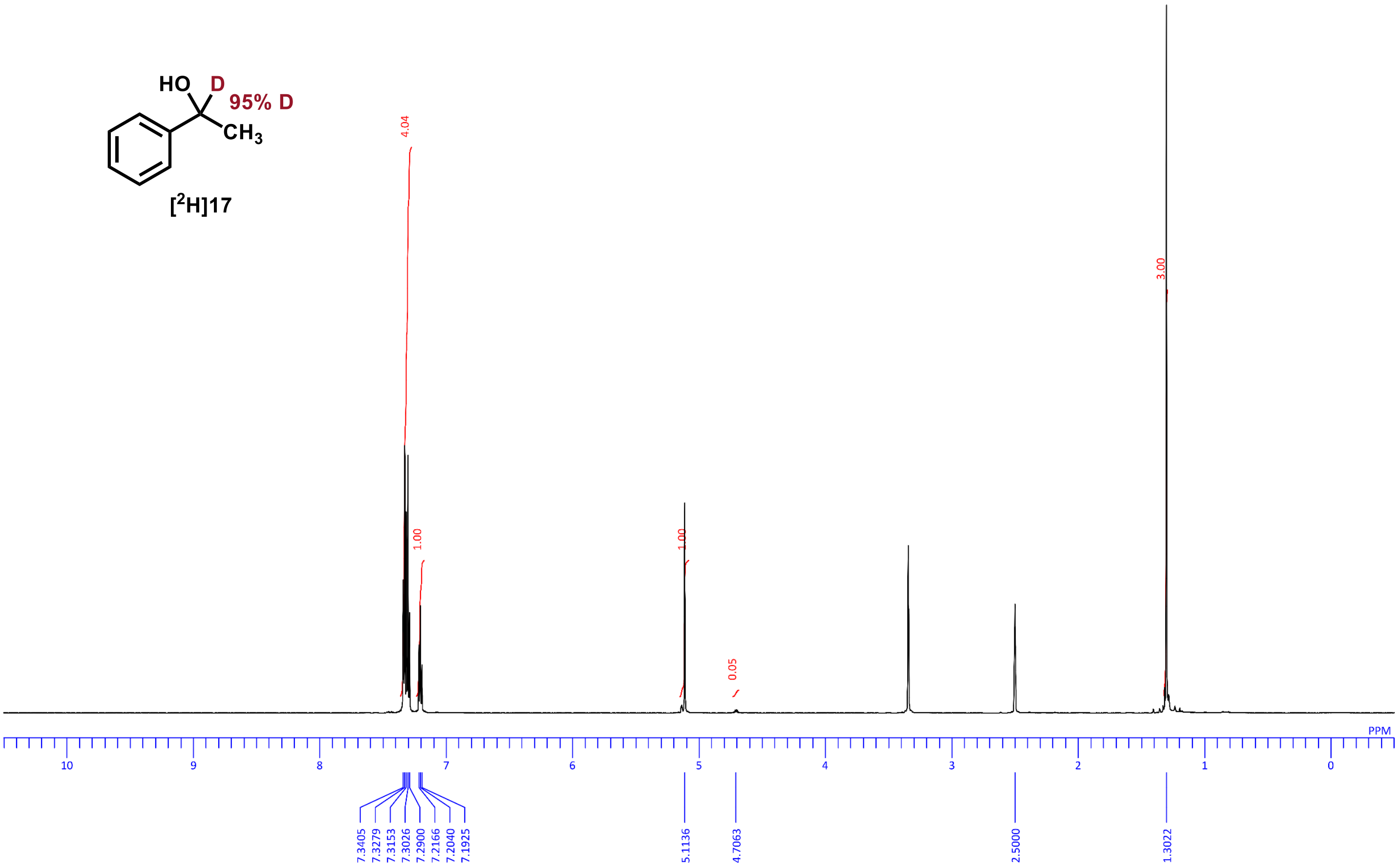
HO D
95% D
CH3
[2H]17
4.04
1.00
1.00
0.05
3.00
PPM
10 9 8 7 6 5 4 3 2 1 0
7.3405 7.3279 7.3153 7.3026 7.2900 7.2166 7.2040 7.1925
5.1136
4.7063
2.5000
1.3022

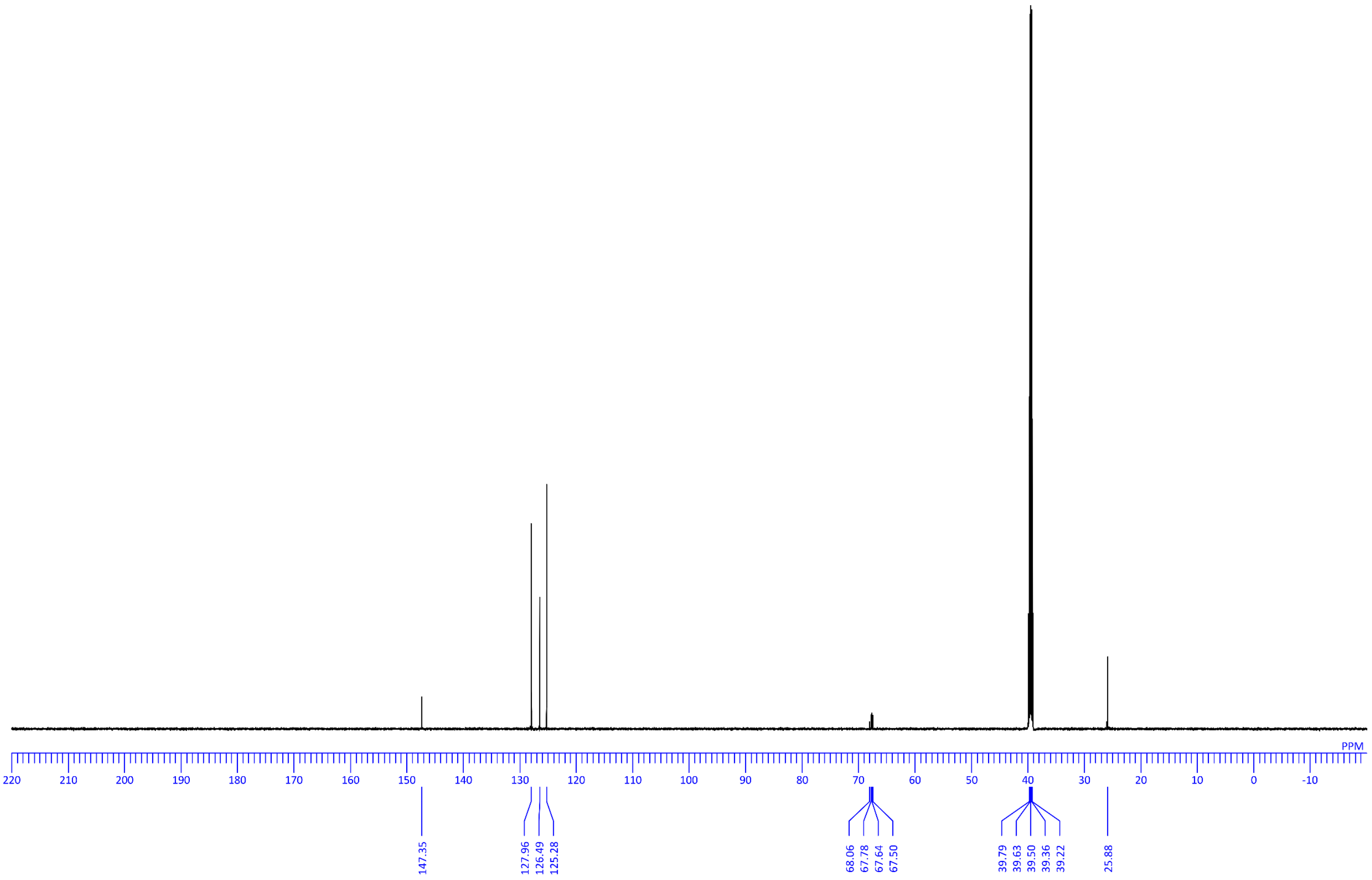
PPM
220 210 200 190 180 170 160 150 140 130 120 110 100 90 80 70 60 50 40 30 20 10 0 -10
147.35
127.96 126.49 125.28
68.06 67.78 67.64 67.50
39.79 39.63 39.50 39.36 39.22
25.88

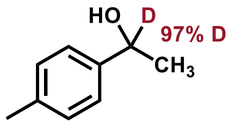
HO
D
97% D
CH3

[^{2}H]18

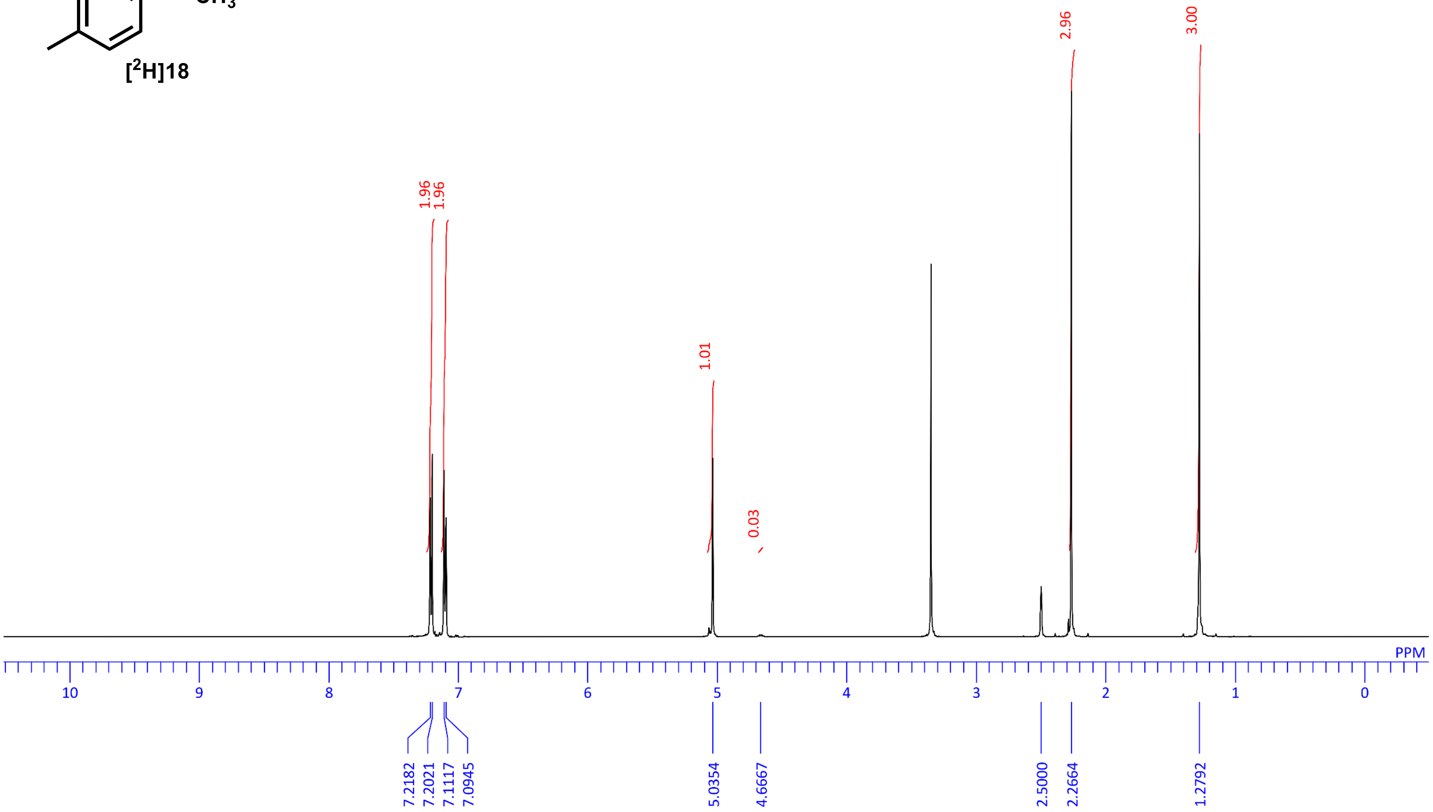
1.96
1.96
1.01
0.03
2.96
3.00
PPM
10
9
8
7
6
5
4
3
2
1
0
7.2182
7.2021
7.1117
7.0945
5.0354
4.6667
2.5000
2.2664
1.2792

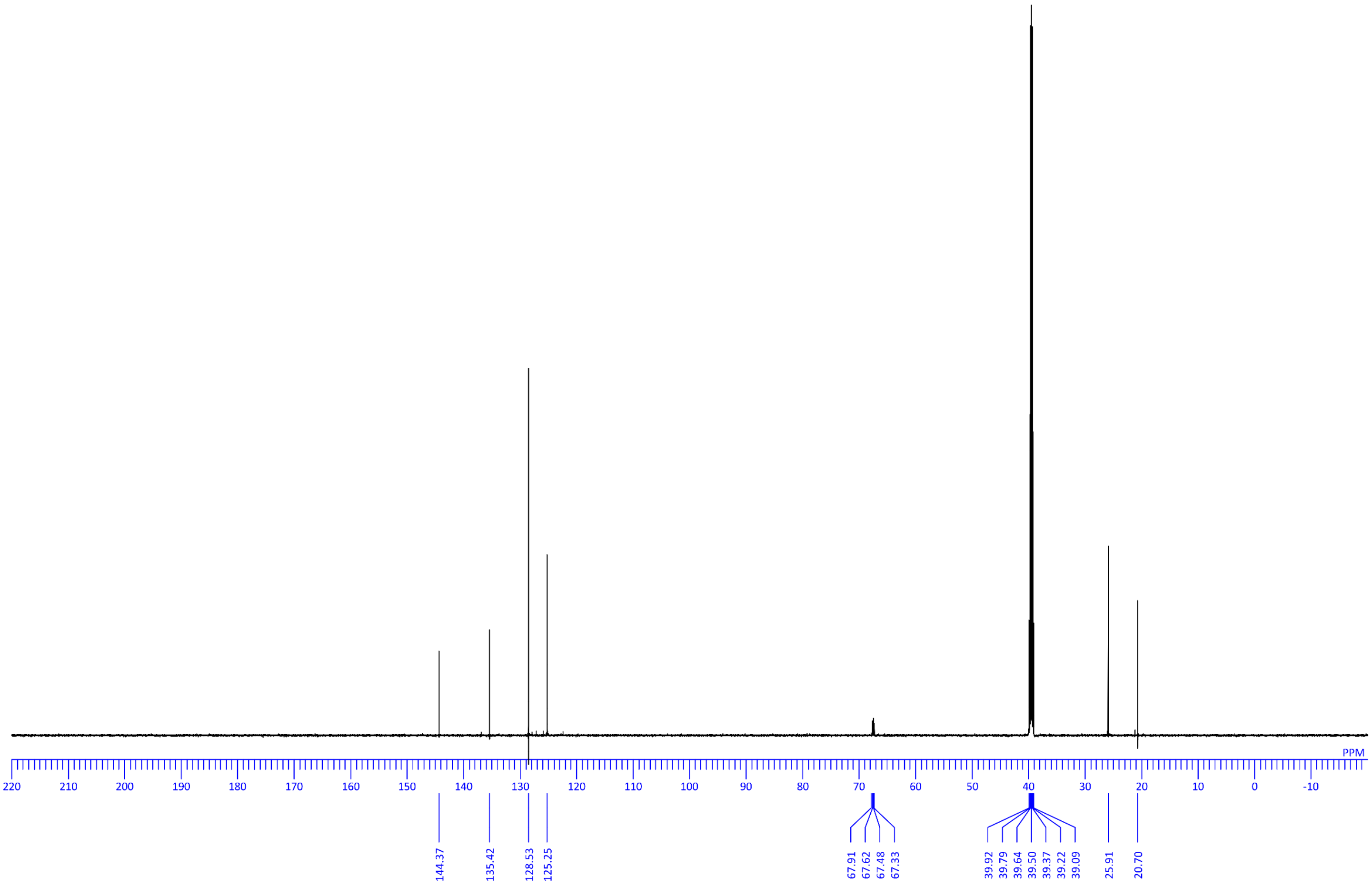
PPM
220
210
200
190
180
170
160
150
140
130
120
110
100
90
80
70
60
50
40
30
20
10
0
-10
144.37
135.42
128.53
125.25
67.91
67.62
67.48
67.33
39.92
39.79
39.64
39.50
39.37
39.22
39.09
25.91
20.70

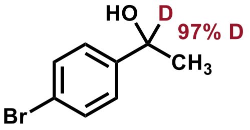
HO
D
97% D
CH3
Br

[^{2}H]19

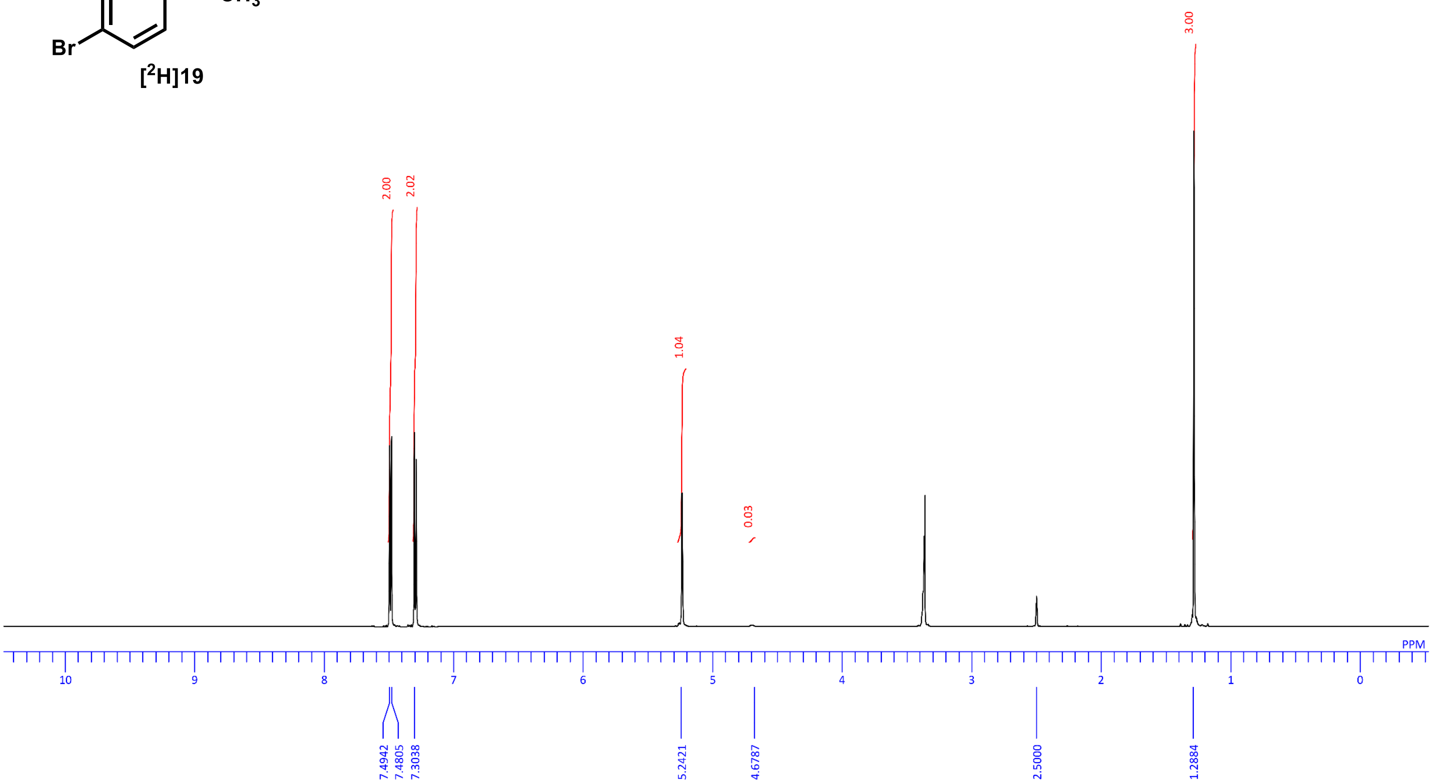
2.00
2.02
1.04
0.03
3.00
PPM
10
9
8
7
6
5
4
3
2
1
0
7.4942
7.4805
7.3038
5.2421
4.6787
2.5000
1.2884

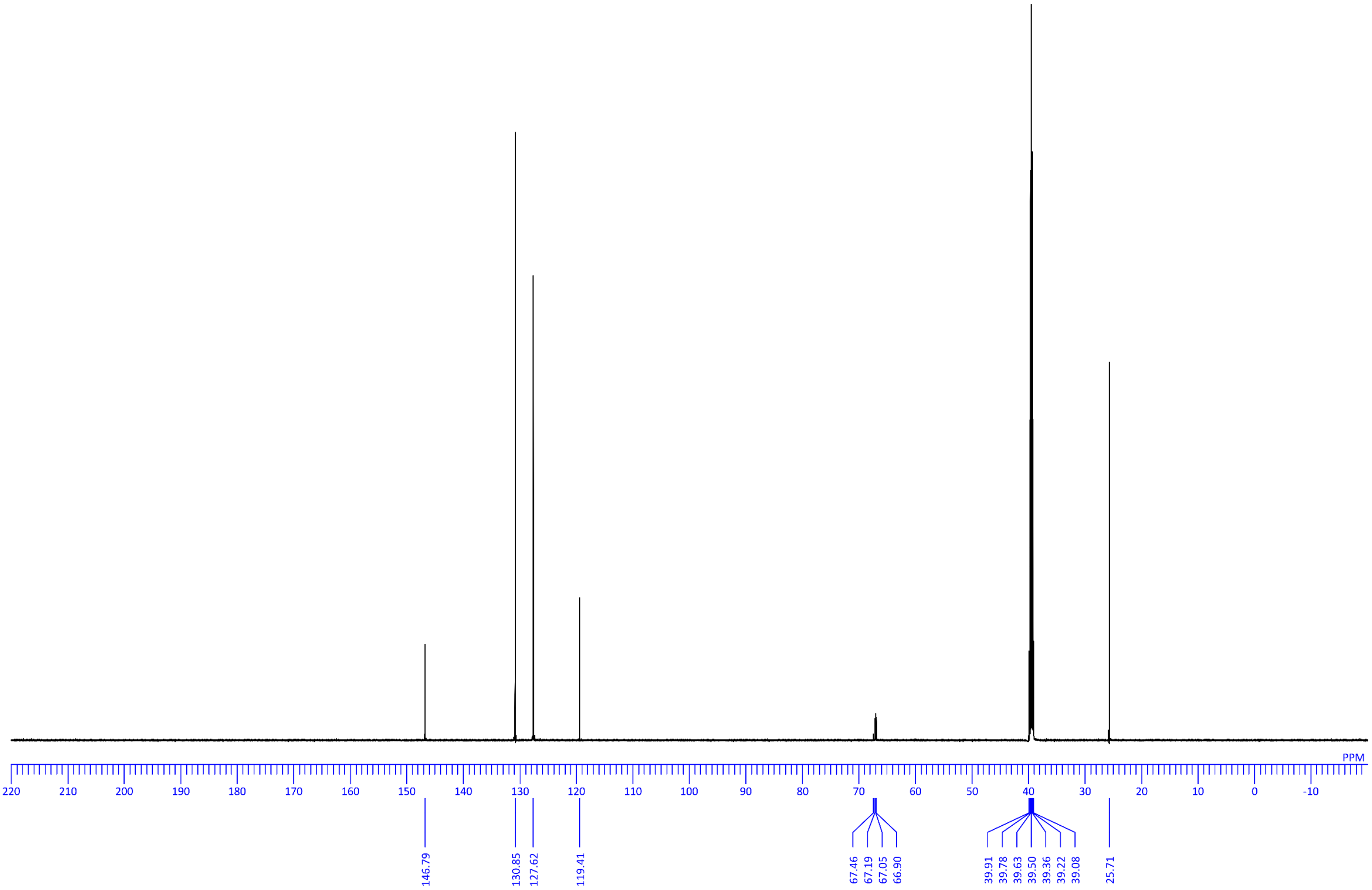
PPM
220
210
200
190
180
170
160
150
140
130
120
110
100
90
80
70
60
50
40
30
20
10
0
-10
146.79
130.85
127.62
119.41
67.46
67.19
67.05
66.90
39.91
39.78
39.63
39.50
39.36
39.22
39.08
25.71

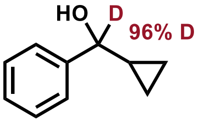
HO
D
96% D

[^{2}H]20

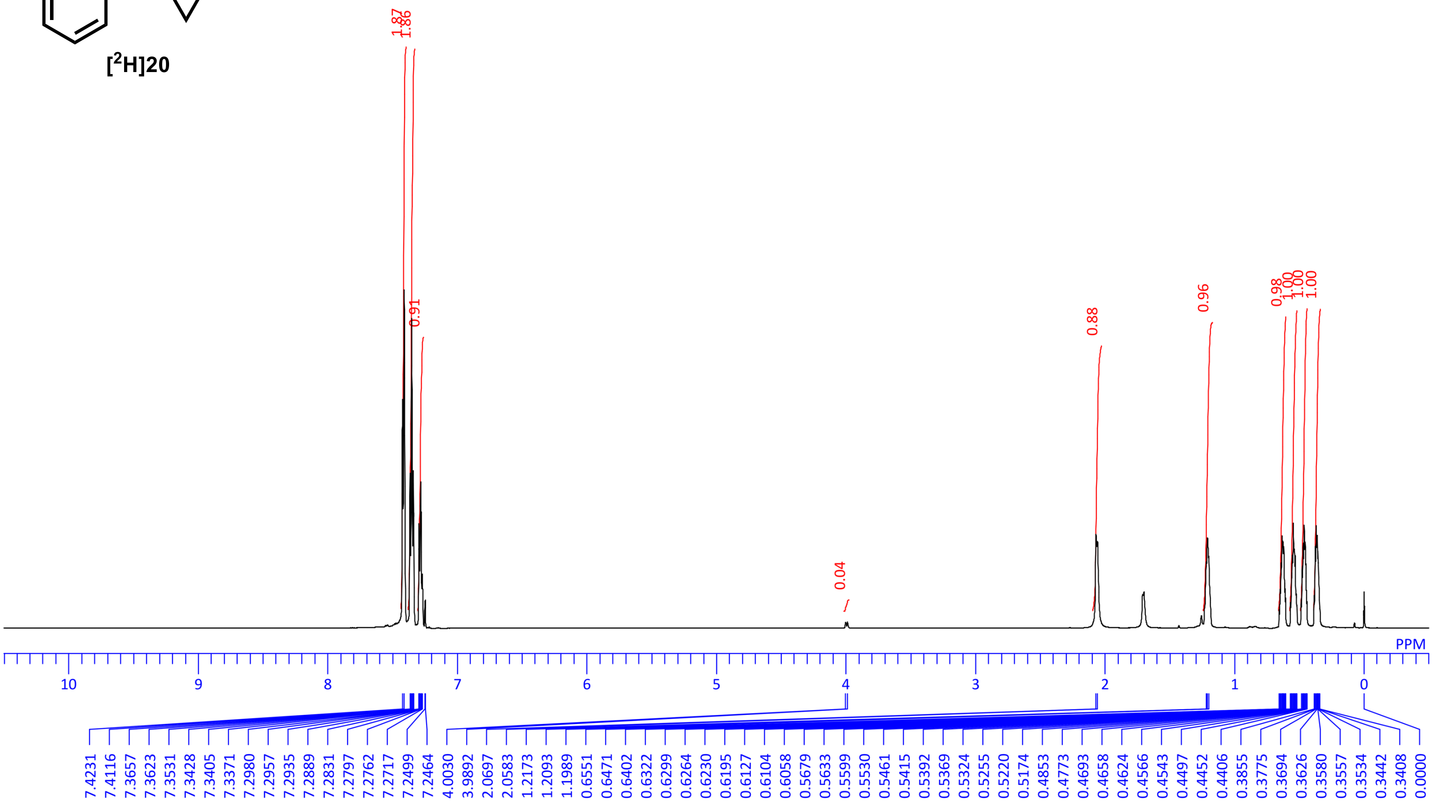
1.87
1.86
0.91
0.04
0.88
0.96
0.98
1.00
1.00
1.00
PPM
10
9
8
7
6
5
4
3
2
1
0
7.4231
7.4116
7.3657
7.3623
7.3531
7.3428
7.3405
7.3371
7.2980
7.2957
7.2935
7.2889
7.2831
7.2797
7.2762
7.2717
7.2499
7.2464
4.0030
3.9892
2.0697
2.0583
1.2173
1.2093
1.1989
0.6551
0.6471
0.6402
0.6322
0.6299
0.6264
0.6230
0.6195
0.6127
0.6104
0.6058
0.5679
0.5633
0.5599
0.5530
0.5461
0.5415
0.5392
0.5369
0.5324
0.5255
0.5220
0.5174
0.4853
0.4773
0.4693
0.4658
0.4624
0.4566
0.4543
0.4497
0.4452
0.4406
0.3855
0.3775
0.3694
0.3626
0.3580
0.3557
0.3534
0.3442
0.3408
0.0000

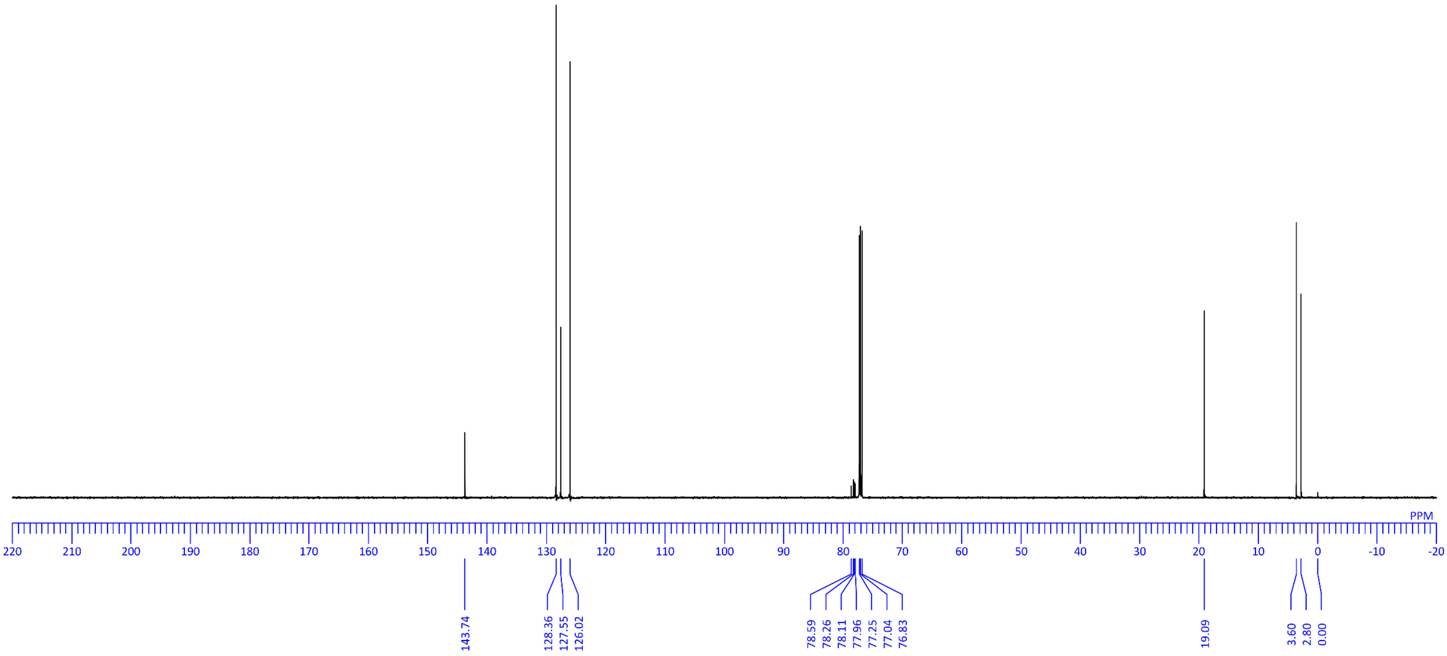
PPM
220
210
200
190
180
170
160
150
140
130
120
110
100
90
80
70
60
50
40
30
20
10
0
-10
-20
143.74
128.36
127.55
126.02
78.59
78.26
78.11
77.96
77.25
77.04
76.83
19.09
3.60
2.80
0.00

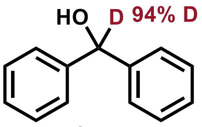
HO
D 94% D

[^{2}H]21

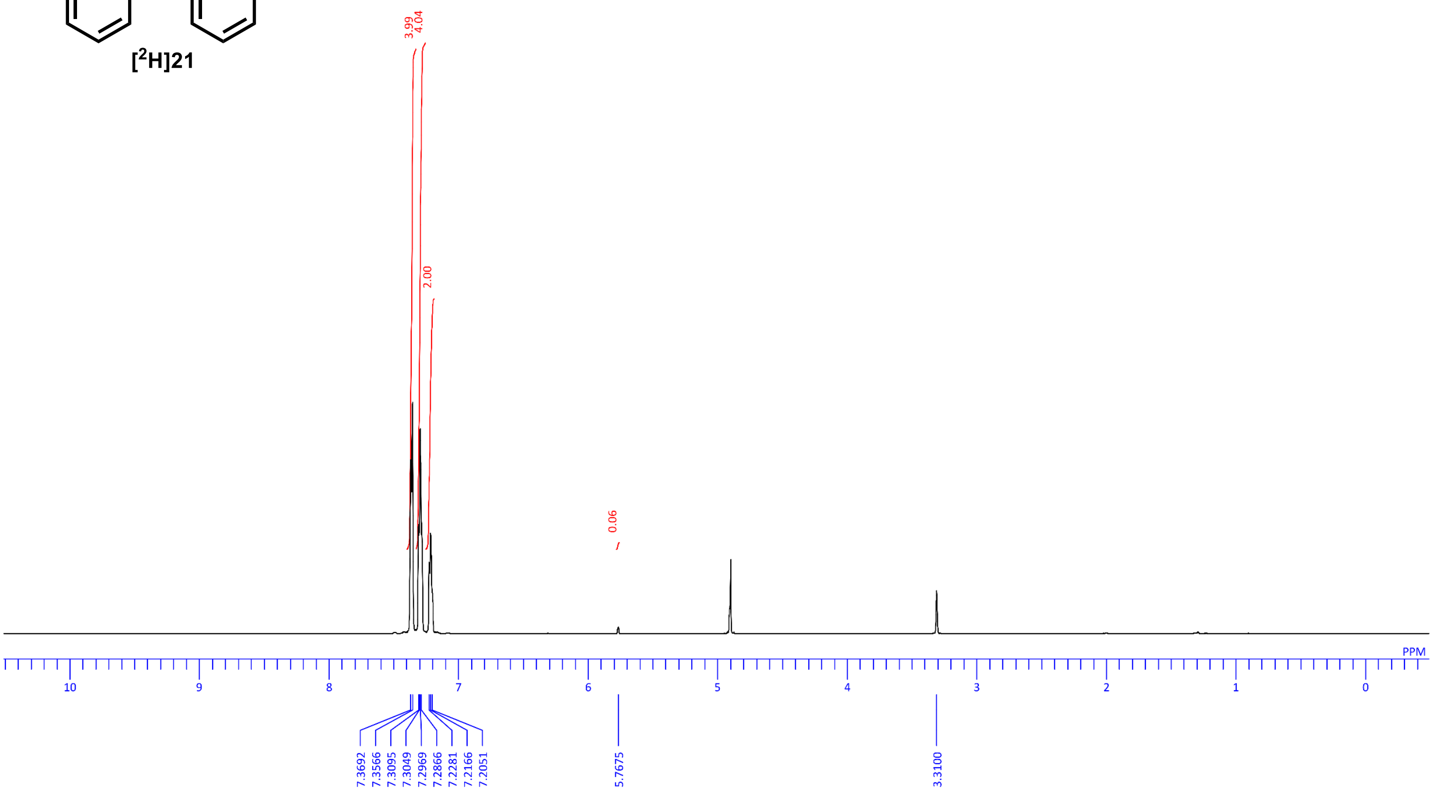
3.99
4.04
2.00
0.06
PPM
7.3692
7.3566
7.3095
7.3049
7.2969
7.2866
7.2281
7.2166
7.2051
5.7675
3.3100

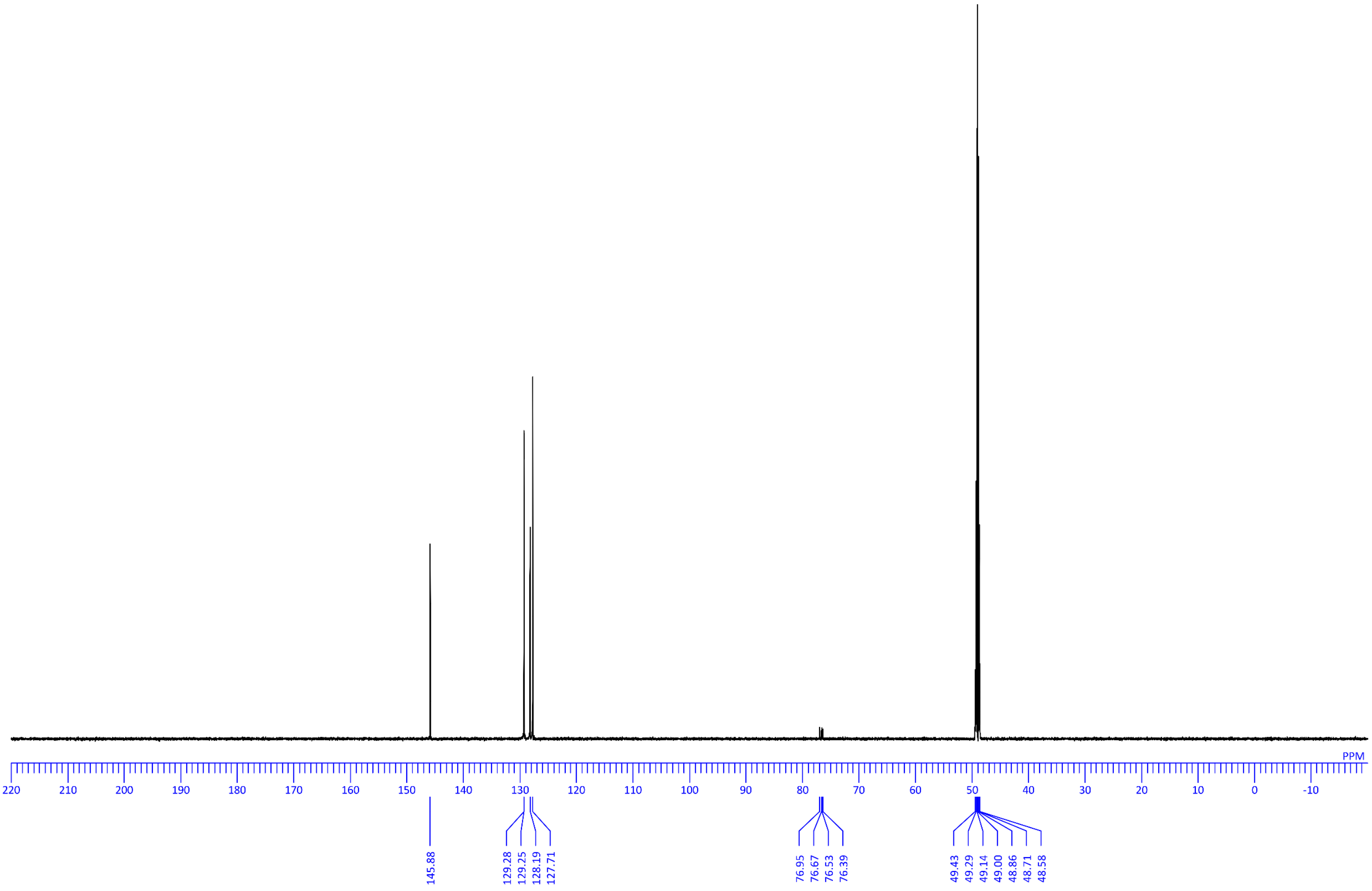
PPM
145.88
129.28
129.25
128.19
127.71
76.95
76.67
76.53
76.39
49.43
49.29
49.14
49.00
48.86
48.71
48.58

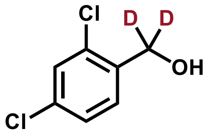
Cl
D
D
OH
Cl

[^{2}H]22

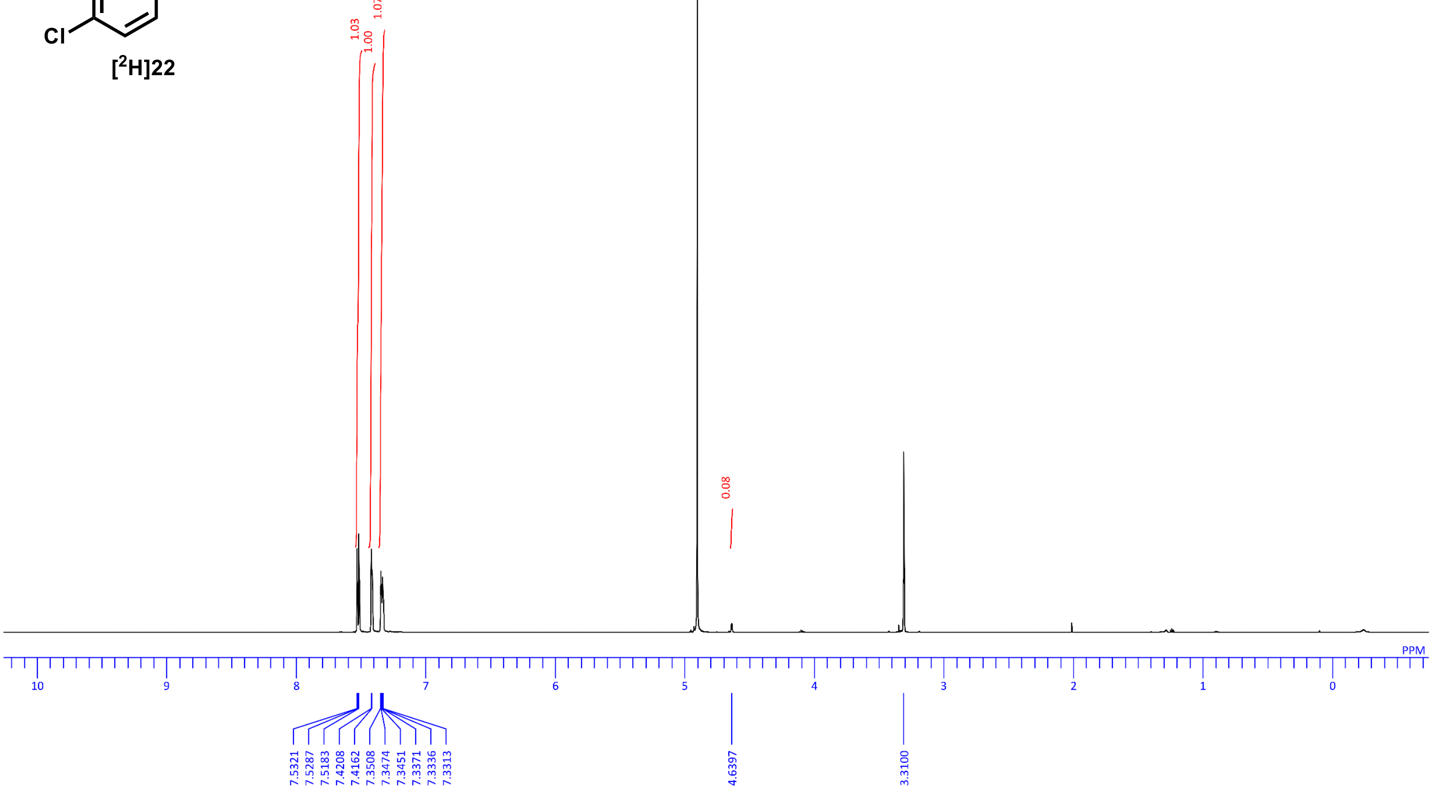
1.03
1.00
1.07
0.08
PPM
10
9
8
7
6
5
4
3
2
1
0
7.5321
7.5287
7.5183
7.4208
7.4162
7.3508
7.3474
7.3451
7.3371
7.3336
7.3313
4.6397
3.3100

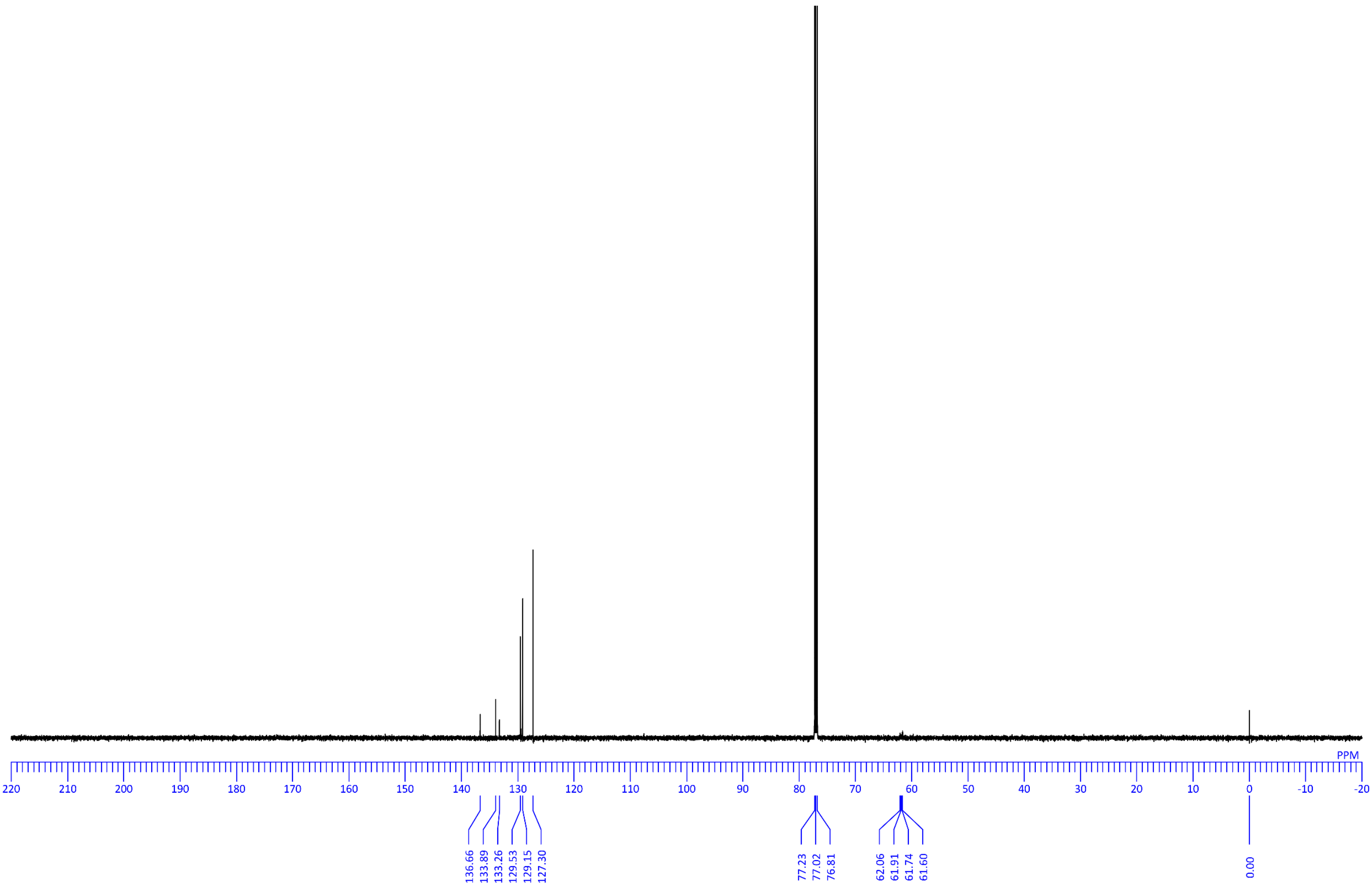
PPM
220
210
200
190
180
170
160
150
140
130
120
110
100
90
80
70
60
50
40
30
20
10
0
-10
-20
136.66
133.89
133.26
129.53
129.15
127.30
77.23
77.02
76.81
62.06
61.91
61.74
61.60
0.00

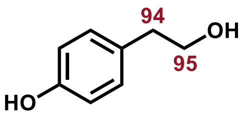
94
OH
95
HO

[^{2}H]23

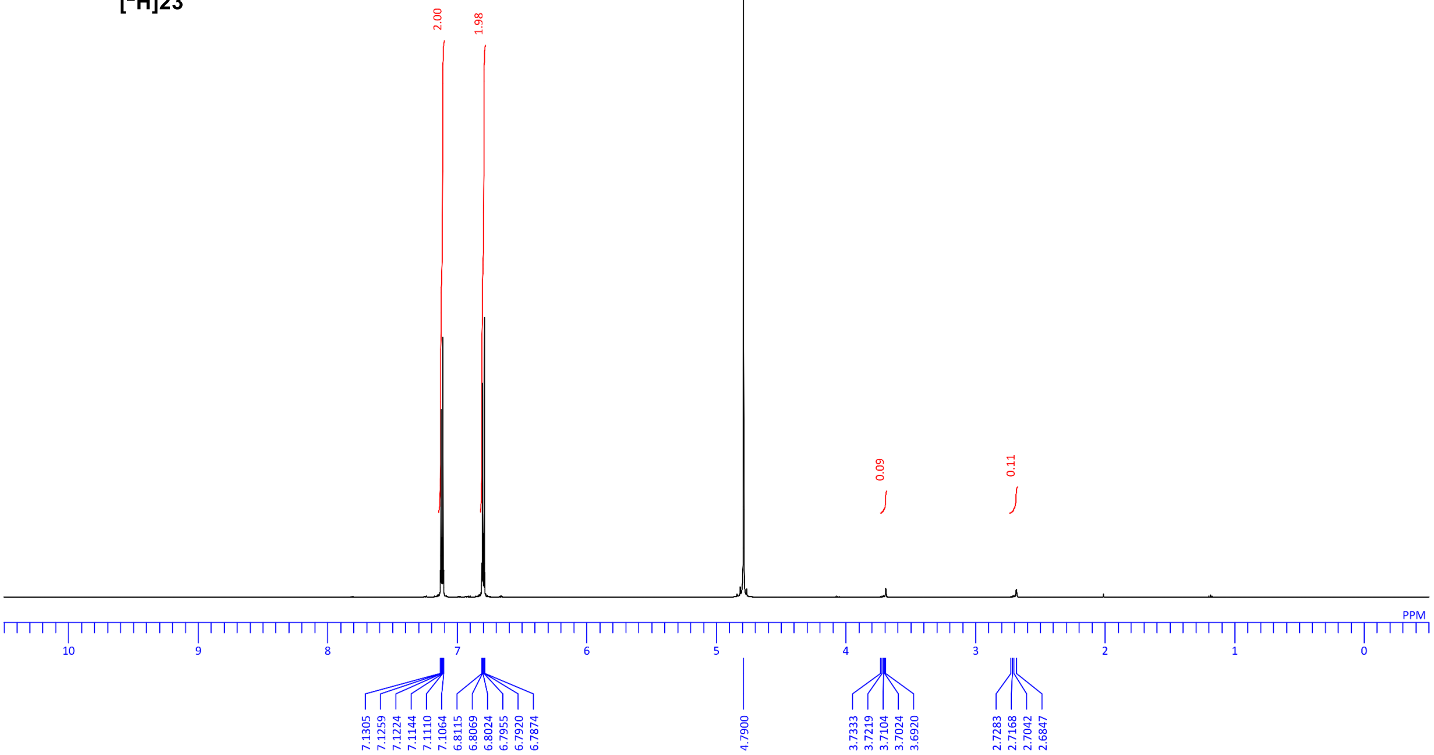
2.00
1.98
0.09
0.11
PPM
10
9
8
7
6
5
4
3
2
1
0
7.1305
7.1259
7.1224
7.1144
7.1110
7.1064
6.8115
6.8069
6.8024
6.7955
6.7920
6.7874
4.7900
3.7333
3.7219
3.7104
3.7024
3.6920
2.7283
2.7168
2.7042
2.6847

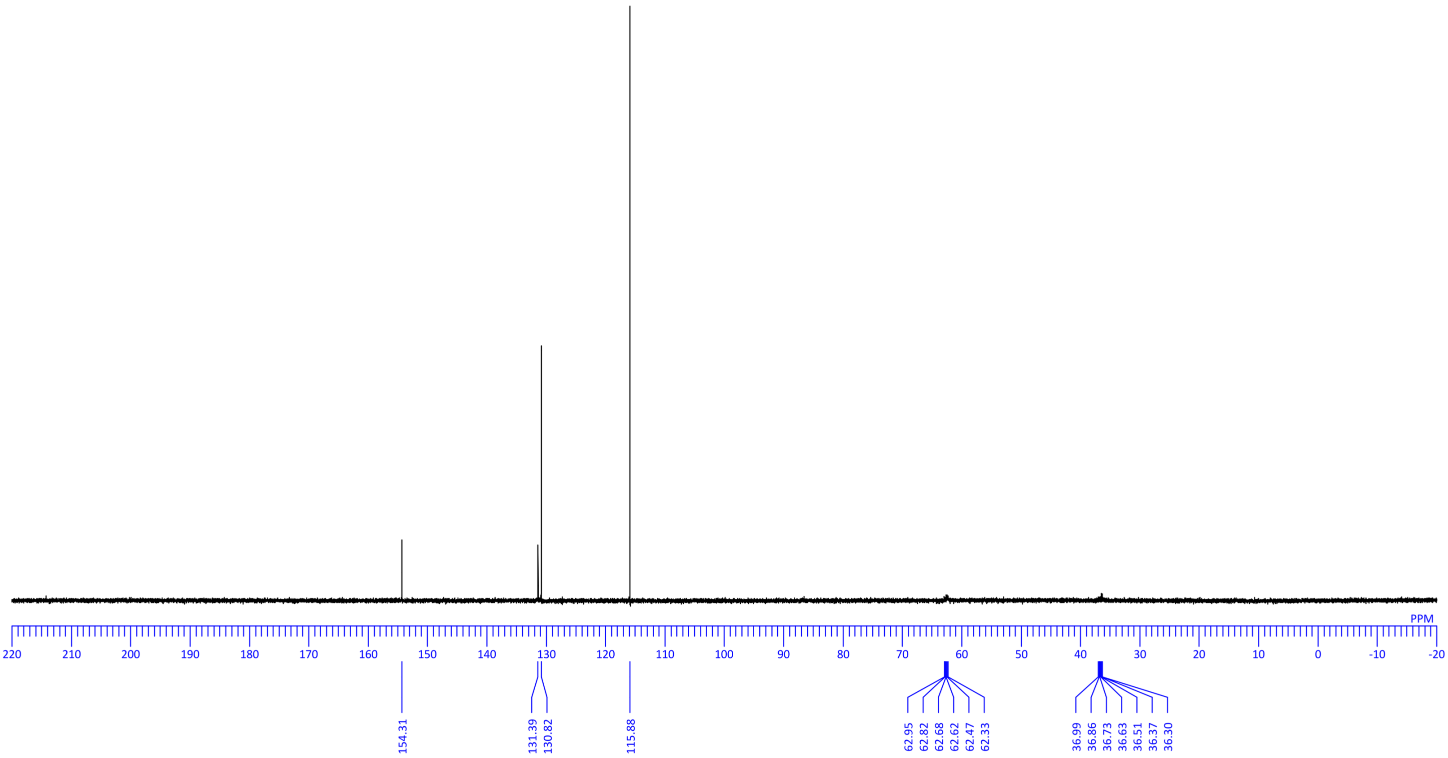
PPM
220
210
200
190
180
170
160
150
140
130
120
110
100
90
80
70
60
50
40
30
20
10
0
-10
-20
154.31
131.39
130.82
115.88
62.95
62.82
62.68
62.62
62.47
62.33
36.99
36.86
36.73
36.63
36.51
36.37
36.30

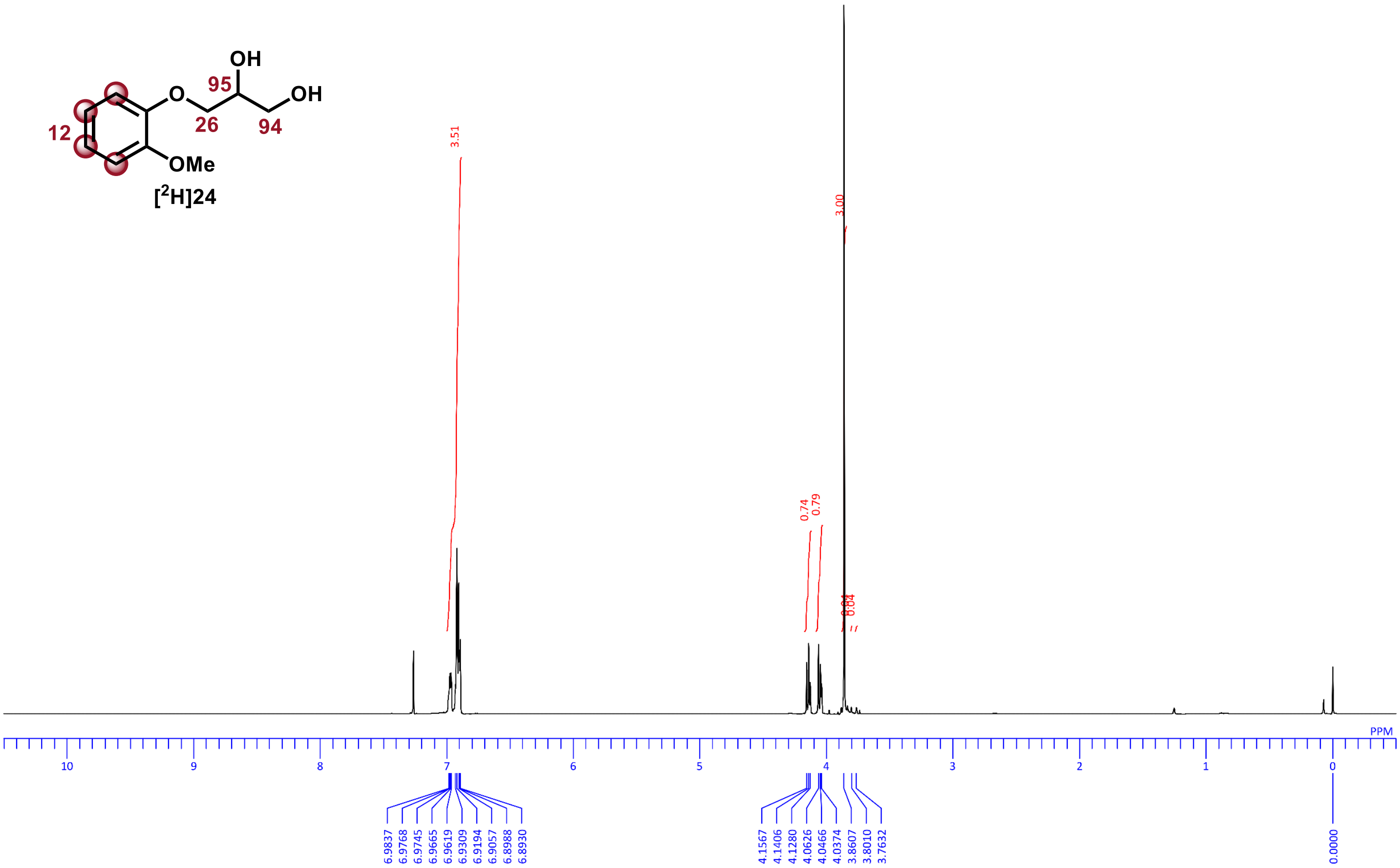
OH
95
O
OH
12
26
94
OMe
[2H]24
3.51
3.00
0.74
0.79
0.04
0.04
PPM
10
9
8
7
6
5
4
3
2
1
0
6.9837
6.9768
6.9745
6.9665
6.9619
6.9309
6.9194
6.9057
6.8988
6.8930
4.1567
4.1406
4.1280
4.0626
4.0466
4.0374
3.8607
3.8010
3.7632
0.0000

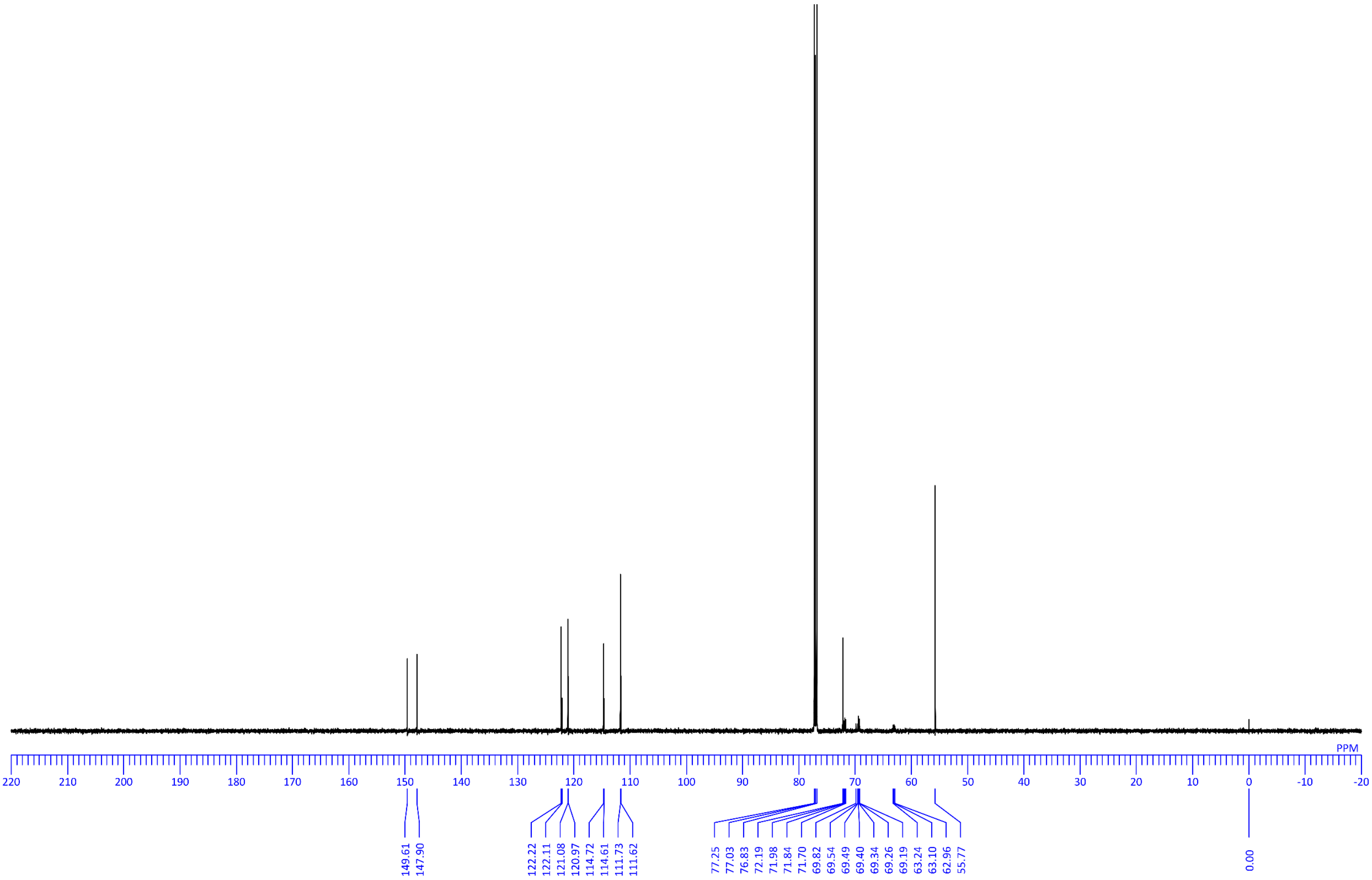
PPM
220
210
200
190
180
170
160
150
140
130
120
110
100
90
80
70
60
50
40
30
20
10
0
-10
-20
149.61
147.90
122.22
122.11
121.08
120.97
114.72
114.61
111.73
111.62
77.25
77.03
76.83
72.19
71.98
71.84
71.70
69.82
69.54
69.49
69.40
69.34
69.26
69.19
63.24
63.10
62.96
55.77
0.00

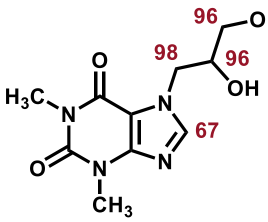
96
OH
98
96
OH
O
H_3C
N
N
67
O
N
N
CH_3

[^{2}H]25

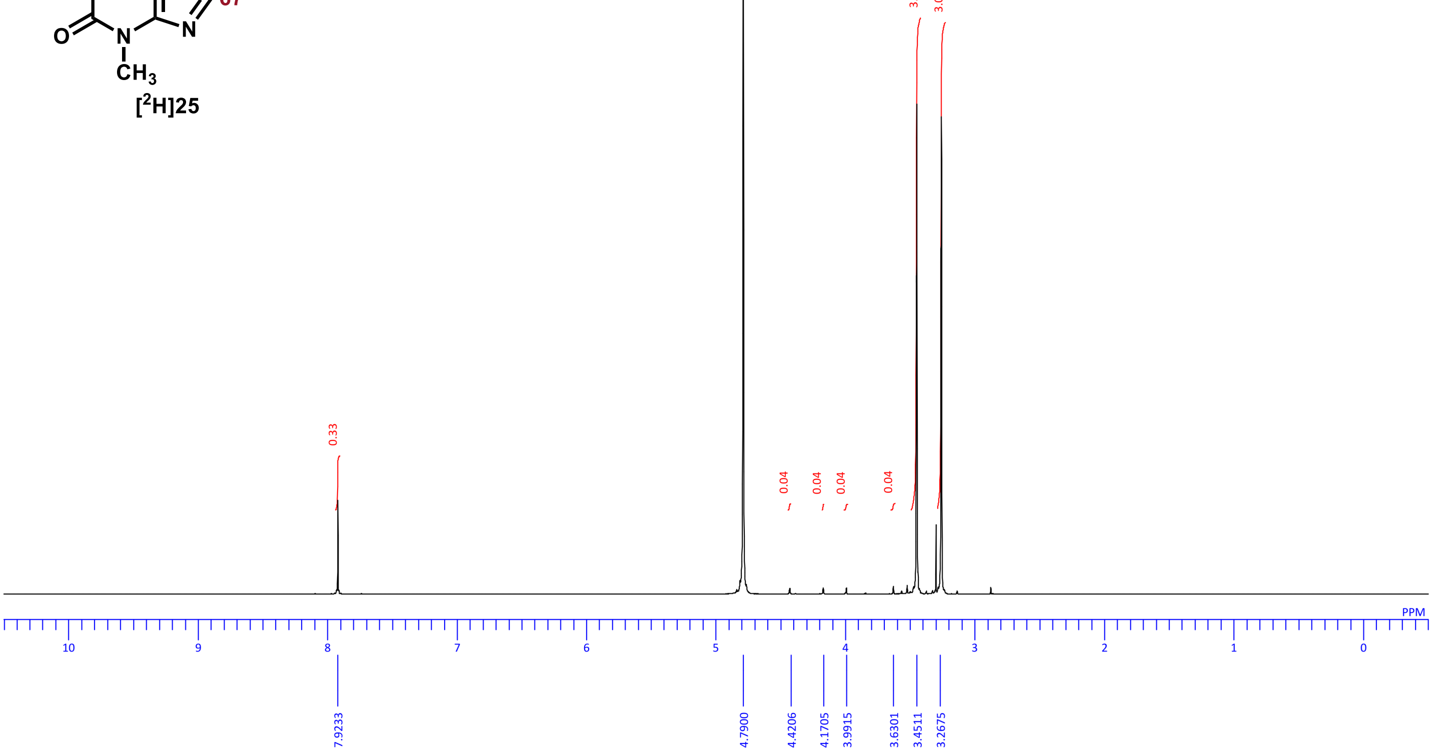
0.33
0.04
0.04
0.04
0.04
3.03
3.00
PPM
10
9
8
7
6
5
4
3
2
1
0
7.9233
4.7900
4.4206
4.1705
3.9915
3.6301
3.4511
3.2675

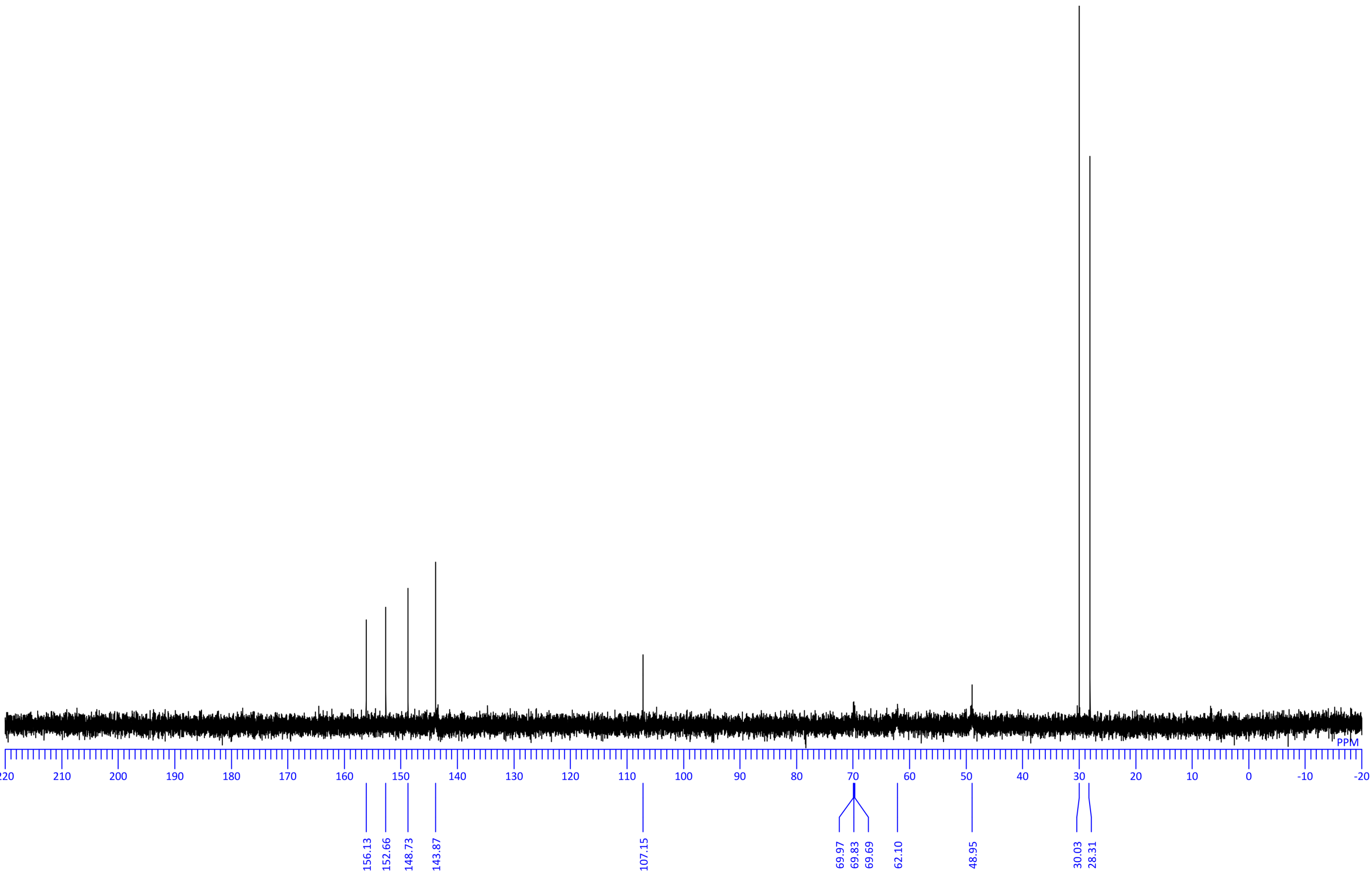
PPM
220
210
200
190
180
170
160
150
140
130
120
110
100
90
80
70
60
50
40
30
20
10
0
-10
-20
156.13
152.66
148.73
143.87
107.15
69.97
69.83
69.69
62.10
48.95
30.03
28.31

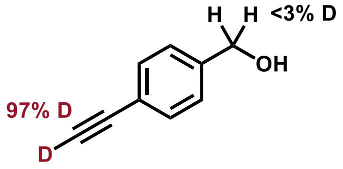
H H <3% D
OH
97% D
D

[^{2}H]26

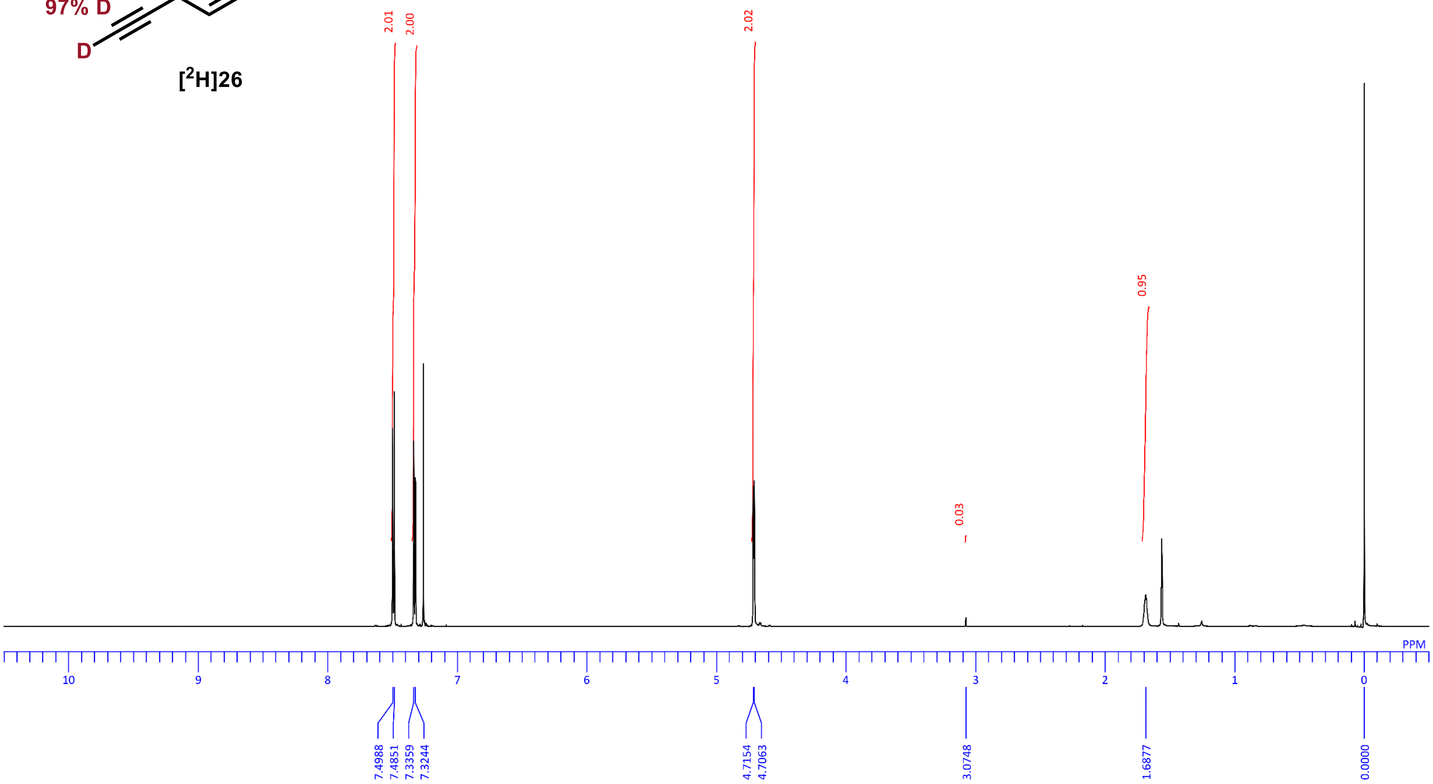
2.01
2.00
2.02
0.03
0.95
PPM
10 9 8 7 6 5 4 3 2 1 0
7.4988
7.4851
7.3359
7.3244
4.7154
4.7063
3.0748
1.6877
0.0000

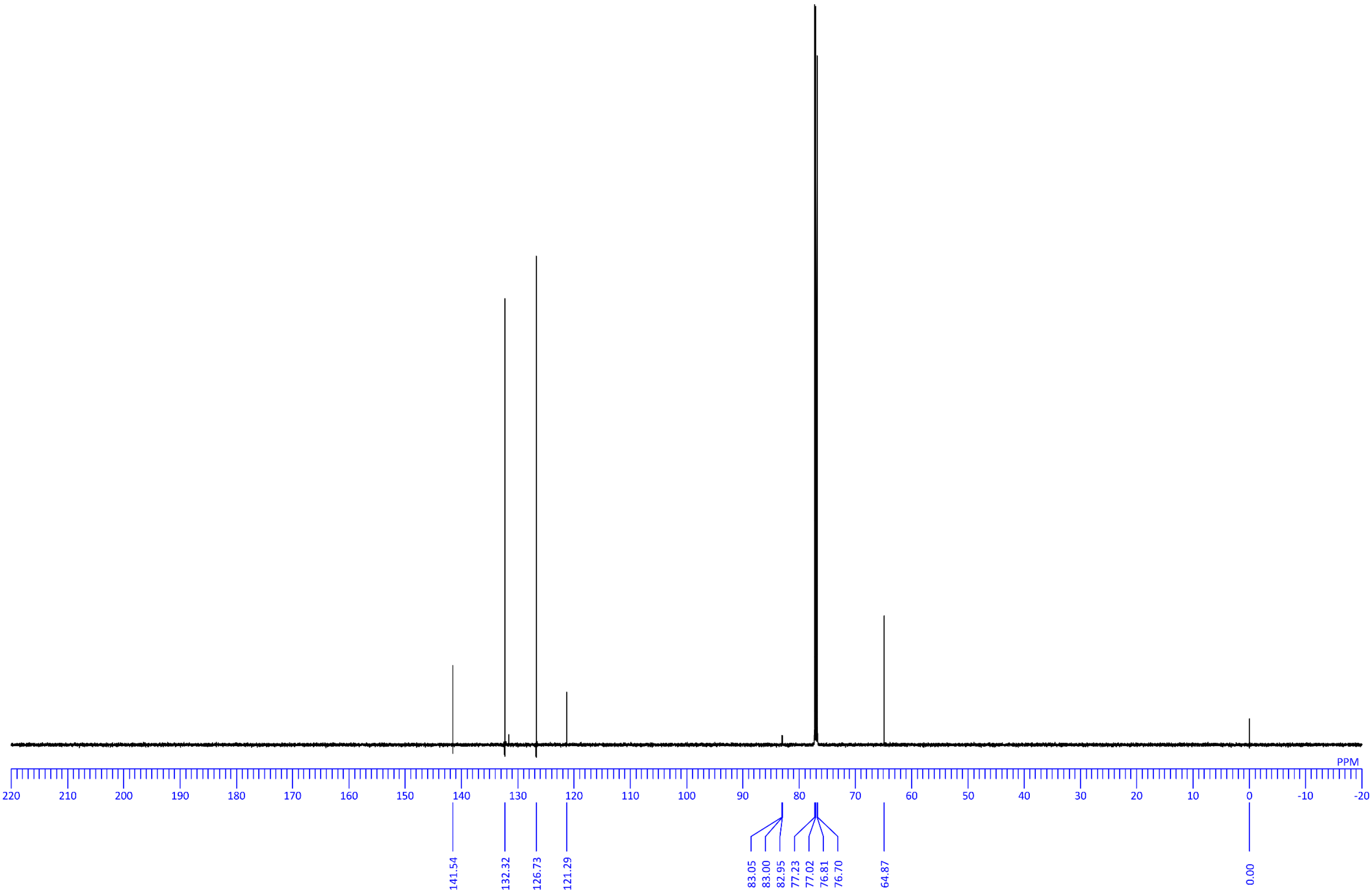
PPM
220 210 200 190 180 170 160 150 140 130 120 110 100 90 80 70 60 50 40 30 20 10 0 -10 -20
141.54
132.32
126.73
121.29
83.05
83.00
82.95
77.23
77.02
76.81
76.70
64.87
0.00

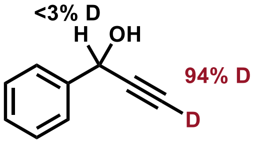
<3% D
H OH
94% D
D

[^{2}H]27

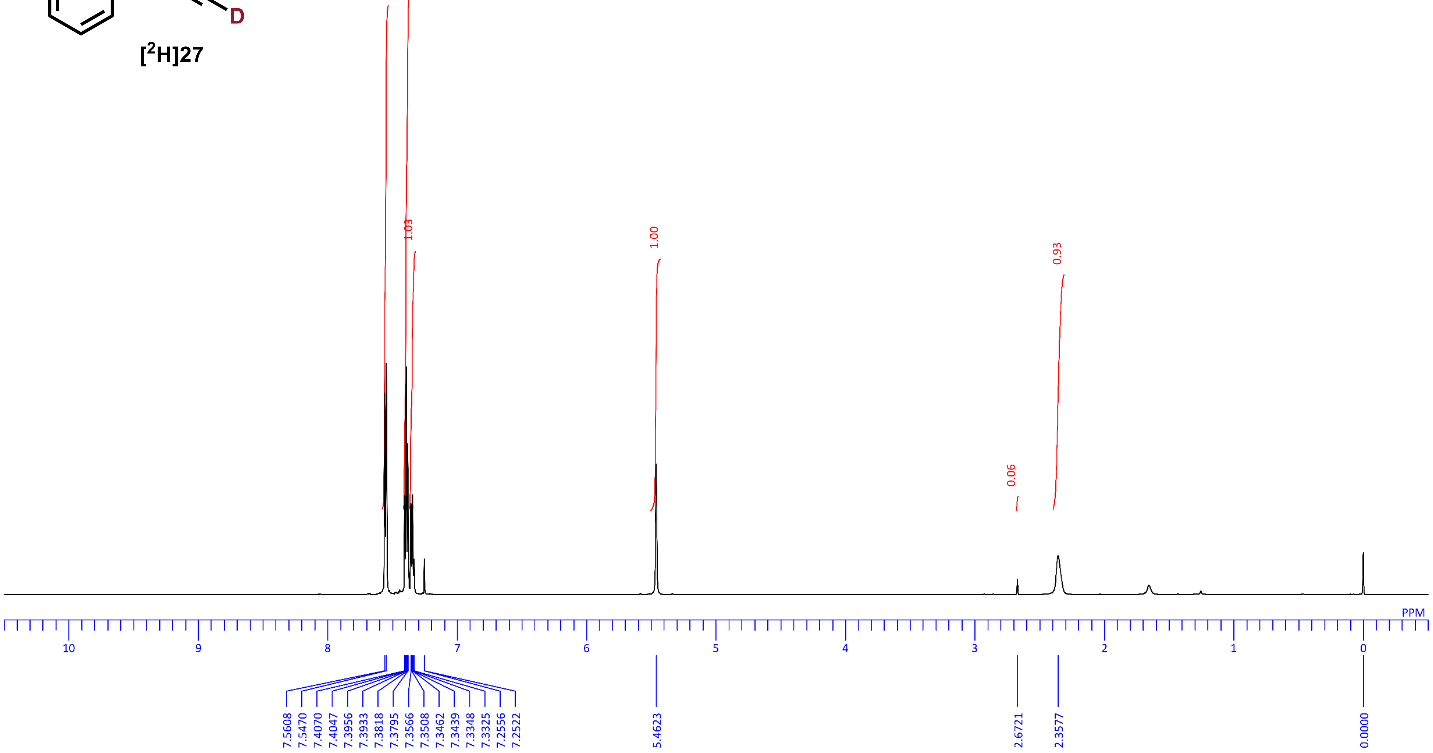
2.00
2.04
1.03
1.00
0.06
0.93
PPM
10
9
8
7
6
5
4
3
2
1
0
7.5608
7.5470
7.4070
7.4047
7.3956
7.3933
7.3818
7.3795
7.3566
7.3508
7.3462
7.3439
7.3348
7.3325
7.2556
7.2522
5.4623
2.6721
2.3577
0.0000

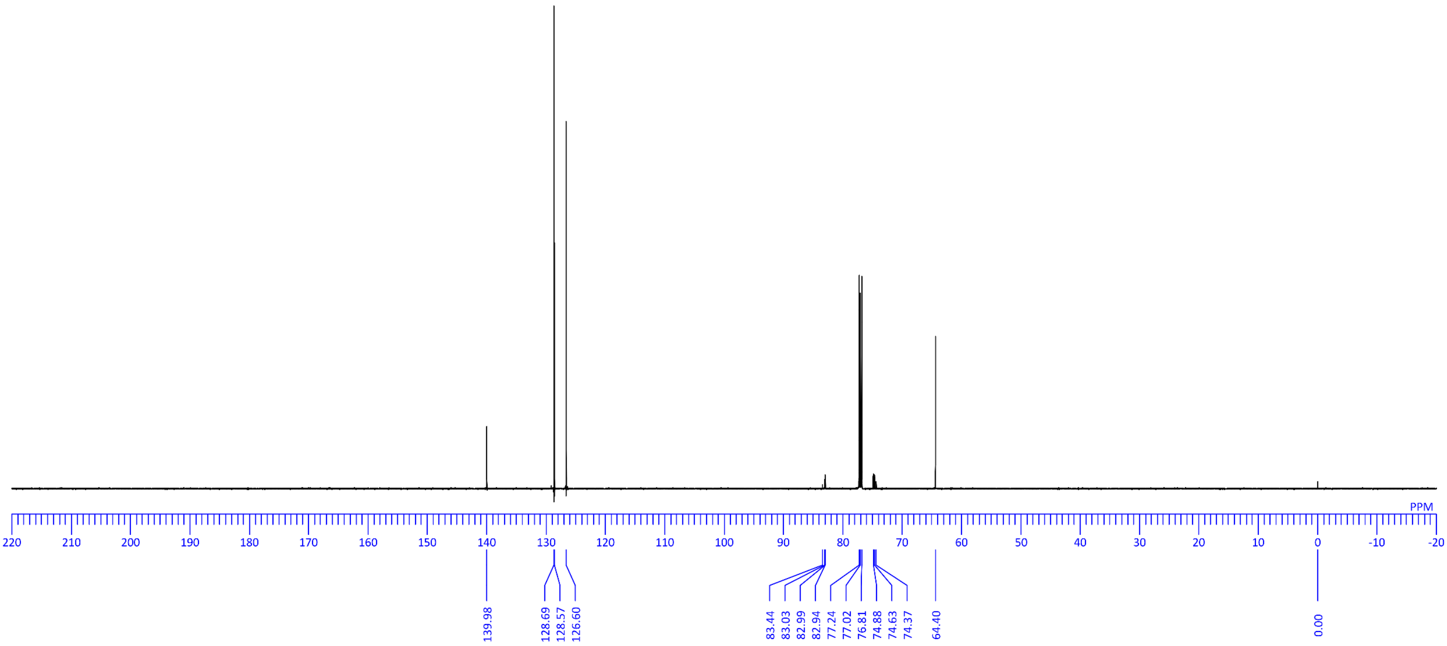
PPM
220
210
200
190
180
170
160
150
140
130
120
110
100
90
80
70
60
50
40
30
20
10
0
-10
-20
139.98
128.69
128.57
126.60
83.44
83.03
82.99
82.94
77.24
77.02
76.81
74.88
74.63
74.37
64.40
0.00
